# Light-Driven Purification of Progesterone from Steroid Mixtures Using a Photoresponsive Metal–Organic Capsule

**DOI:** 10.1021/jacs.3c11005

**Published:** 2024-01-17

**Authors:** Amit Ghosh, Jiratheep Pruchyathamkorn, Carles Fuertes Espinosa, Jonathan R. Nitschke

**Affiliations:** Yusuf Hamied Department of Chemistry, University of Cambridge, Cambridge CB2 1EW, U.K.

## Abstract

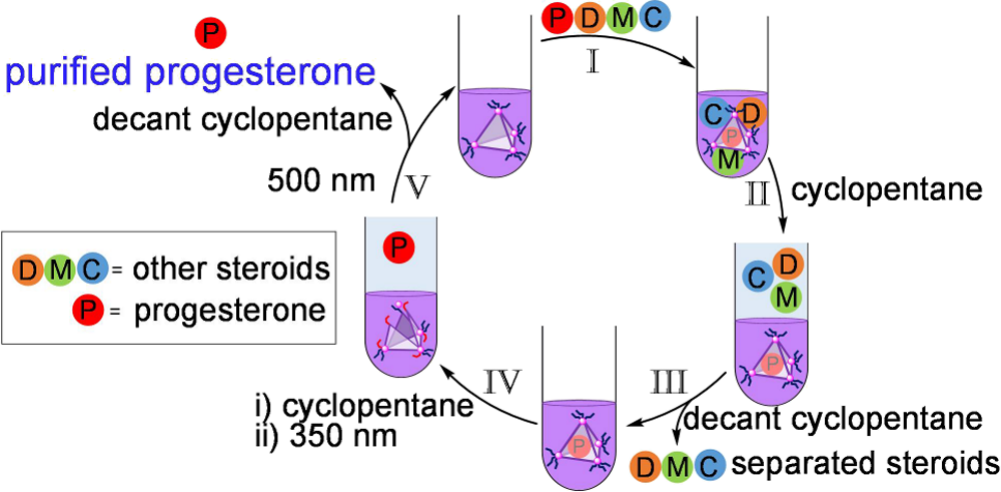

Chemical separations are expensive, consuming 10–15% of humanity’s global energy budget. Many current separation methods employ thermal energy for distillation, often through the combustion of carbon-containing fuels, or extractions and crystallizations from organic solvents, which must then be discarded or redistilled, with a substantial energetic cost. The direct use of renewable energy sources, such as light, could enable the development of novel separations processes, as is required for the transition away from fossil fuel use. Metal–organic capsules, which can selectively bind molecules from mixtures, can provide the foundation for these novel separations processes. Here we report a tetrahedral metal–organic capsule bearing light-responsive diazo moieties around its metal-ion vertices. This capsule can be used to selectively separate progesterone from a mixture of steroids in a process driven by visible light energy. Our process combines biphasic extraction and selective binding of progesterone with the light-driven release of this molecule in purified form. Ultimately, our process might be adapted to the purifications of the many other fine chemical products that are bound selectively by capsules.

## Introduction

In recent years, metal–organic coordination capsules^1−8^ have been developed to bind various species^9−12^ within their internal cavities, enabling applications that include new means of drug delivery,^13^ catalysis,^14,15^ and chemical separations.^16,17^ The use of capsules for separations has the potential to enable industrially relevant compounds to be captured selectively from feedstocks and isolated in pure form. Among the various stimuli used to trigger guest release,^18−22^ light is particularly attractive, as it is easy to obtain and focus, and it does not generate any chemical waste products.^23−29^ Recent studies have revealed that light can reversibly control guest uptake and release processes from metal–organic cages that integrate photoswitchable moieties.^30−33^

Complex drug molecules, such as steroids, are often challenging to purify from similar precursor molecules or side-products, following synthesis or biological production. Steroids are generally isolated by extraction with organic solvents. This process can denature proteins associated with the steroids, which may also be extracted alongside the steroids. Steroids may then be purified by chromatography,^34^ repeated crystallization,^35^ or partitioning between solvents with different polarities.^36^ These processes require large volumes of solvent that must be discarded or redistilled, at great energetic expense.

**Figure 1:**
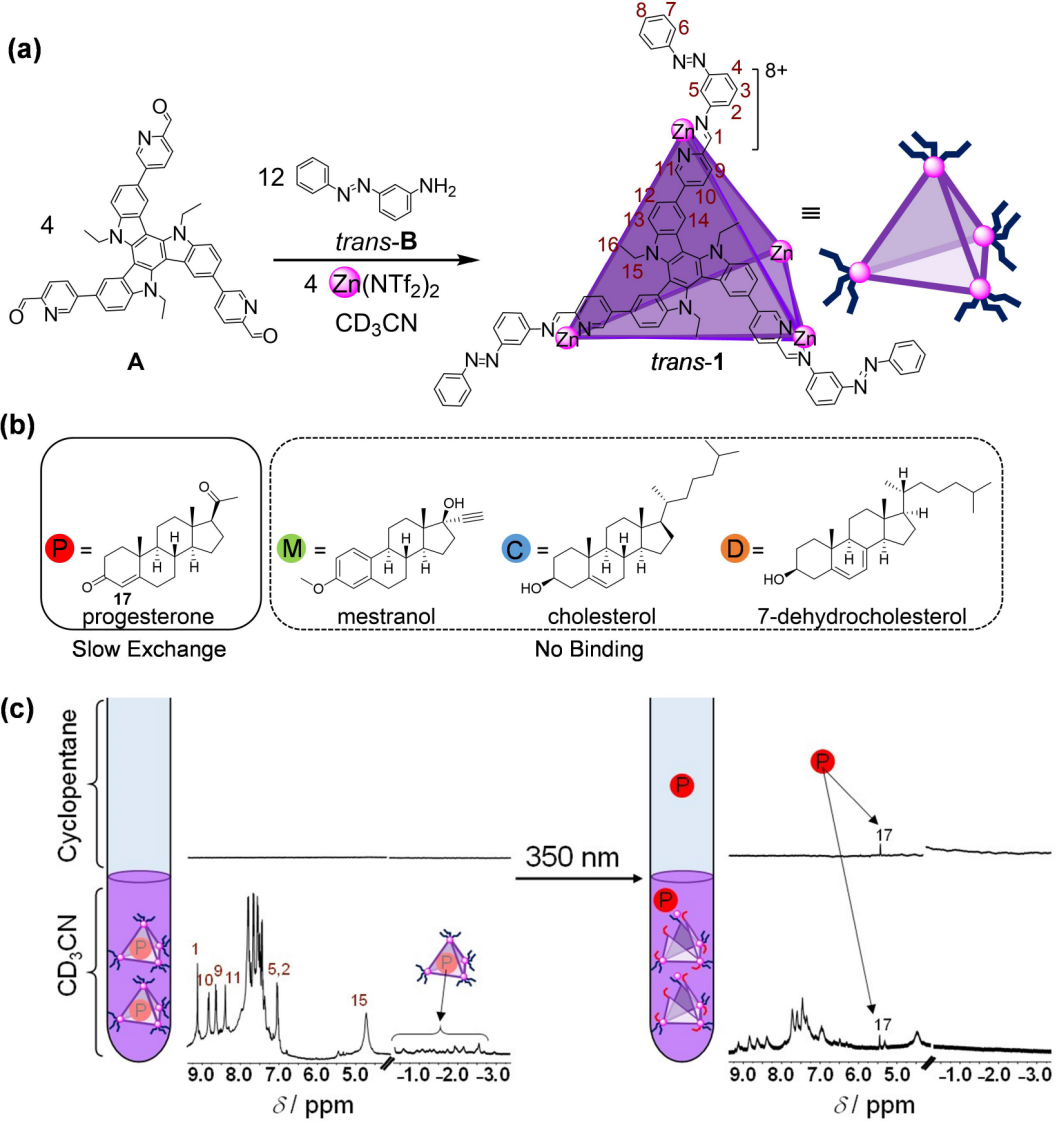
Self-assembly of photoresponsive cage *trans*-**1**. (a) The assembly of subcomponents **A** and *trans*-**B** with Zn^II^ produced cage *trans*-**1**; only one of the four ligands that form faces of the tetrahedron is shown. (b) Steroid guests investigated for binding within *trans*-**1**. Progesterone bound in slow exchange on the ^1^H NMR time scale. No binding was observed for mestranol, cholesterol and 7-dehydrocholesterol, which are represented as green, blue and orange circles, respectively. (c) Light-driven progesterone release from *trans*-**1** in a biphasic solvent system. CD_3_CN and cyclopentane formed two distinct immiscible solvent layers. Slice-selective ^1^H NMR^42^ indicated that the complex of progesterone inside *trans*-**1** dissolved only in CD_3_CN. Ultraviolet irradiation triggered cage disassembly and concomitant progesterone release. Slice-selective ^1^H NMR revealed that the released progesterone distributed across both solvent phases.

Here we introduce the use of coordination cage **1** (Figure 1a) as a synthetic steroid receptor^37,38^ that selectively bound and purified progesterone, a naturally occurring steroid and drug that is central to women’s reproductive health and fertility.^39^ More than a million tons of progesterone and related steroidal drugs are produced annually, with an estimated global market value of US$10 billion in 2020.^40^

As shown in Figure 1a, **1** contains 12 diazo moieties at its cationic vertices, which adopt the *trans* configuration in the ground state. Light triggers these diazo groups to isomerize to the thermodynamically unfavorable *cis* configuration, creating steric clashes that result in dissociation of the cage vertices, which results in the release of bound progesterone. This purified progesterone may then be extracted from the opened cage. Irradiation at a longer wavelength subsequently converts the *cis* diazo moieties back to the *trans* configuration, recovering the cage for further use. This cycle thus demonstrates a straightforward method for the purification of a valuable steroid, highlighting a potential alternative to current separation methods.

## Results and Discussion

### Self-Assembly and Photo-Isomerization of Capsule **1**

We hypothesized that the incorporation of phenyldiazenyl-functionalized subcomponent *trans*-**B** at the vertices of cage **1** would enable the use of light to control the uptake and release of steroid guests,^37^ based upon prior work involving light-driven anion uptake and release.^30^ Tritopic triazatruxene trialdehyde subcomponent **A**(37) (4 equiv) and *trans*-**B** (12 equiv) reacted with zinc(II) bis(trifluoromethanesulfonyl)imide (triflimide, Tf_2_N^–^, 4 equiv) in acetonitrile, yielding the tetrahedral capsule *trans*-**1** (Figure 1a) as the uniquely observed product. *trans*-**1** was characterized by NMR spectroscopy (Figures S2–S9) and electrospray ionization mass spectrometry (ESI-MS) (Figures S10 and S11). The ^1^H NMR spectra of *trans*-**1** exhibited only one set of ligand signals, consistent with the exclusive formation of a pair of T-symmetric tetrahedral enantiomers with a single set of face and vertex stereochemical configurations (Figure S2).^41^ A ^1^H DOSY spectrum of the cage corroborated the presence of a single species with a diffusion coefficient (*D*) of 3.2 × 10^–10^ m^2^·s^–1^ (Figure S7), corresponding to a hydrodynamic radius of 20.3 Å, consistent with our posited tetrahedral structure.

Upon illumination at 350 nm, the *trans*-azobenzene moieties at the vertices of *trans*-**1** progressively underwent photoisomerization to give the *cis* isomer. This photochemical transformation was monitored by ^1^H NMR (Figures S13–S15). The ^1^H NMR spectra exhibited multiple sets of ligand signals, consistent with the formation of a mixture of cages containing both *cis*- and *trans*-diazo moieties (Figure S13). As anticipated,^30^ photoswitching was inferred to lead to steric collisions between *cis*-diazo groups, prompting partial disassembly of the cage. This process was marked by the appearance of ^1^H NMR signals for subcomponents **A** and *cis*-**B** (Figure S14). Following irradiation at 500 nm, this partial disassembly reversed, regenerating cage *trans*-**1**, as confirmed by ^1^H NMR (Figure S15).

### Selective Progesterone Encapsulation

Based upon previous results,^37^ we investigated the ability of *trans*-**1** to bind progesterone, mestranol, cholesterol and 7-dehydrocholesterol (Figure 1b). The host–guest complexes were characterized by ^1^H NMR (500 MHz, CD_3_CN, 0 °C), ^1^H–^1^H COSY, DOSY, mass spectroscopy and isothermal titration calorimetry (ITC). Addition of progesterone to a solution of *trans*-**1** gave rise to a new set of NMR peaks, consistent with guest binding in slow exchange on the ^1^H NMR time scale (Figure S23). Progesterone⊂*trans*-**1** exhibited upfield-shifted signals in the range −3 to 0 ppm (Figure S17), consistent with close contacts between encapsulated progesterone and the aromatic panels lining the cage cavity. We infer progesterone exchange to take place through the faces of the cage, on a faster time scale than dynamic ligand exchange.^43^ The thermodynamics of progesterone binding were also probed using ITC (Figure S25). ITC data fitted to a 1:1 binding profile, with a binding constant of 1.73 (±0.23) × 10^5^ M^–1^. Diffusion spectroscopy (DOSY, Figure S20), and ESI-MS (Figures S21 and S22) also confirmed progesterone inclusion within *trans*-**1**. Numerous attempts to obtain single crystals suitable for X-ray diffraction of progesterone⊂*trans*-**1** as well as empty cage *trans*-**1** were unsuccessful. An increase in the cage hydrodynamic radius (Figures S7 and S20) is consistent with the ethyl substituents of *trans*-**1** switching from being directed inward to the empty cavity, to outward, following the binding of progesterone (Figure S62).

Progesterone (volume = 367 Å^3^) fits well within *trans*-**1** (cavity volume = 715 Å^3^), with a 51% occupancy close to the 55% optimum,^44^ whereas attempts to encapsulate mestranol, cholesterol and 7-dehydrocholesterol within *trans*-**1** provided no ^1^H NMR evidence of binding (Figures S27–S32). We infer that the more rigid aromatic steroid mestranol did not interact well with the cage interior, disfavoring its binding.^37^ Steroids bearing long alkyl chains (cholesterol and 7-dehydrocholesterol) do not fit within *trans*-**1** cavity, preventing their binding.^38^ When mestranol, cholesterol and 7-dehydrocholesterol were added to progesterone⊂*trans*-**1**, no changes to the ^1^H NMR spectra were observed (Figures S34–S37). Future work may enable the use of pairwise competition experiments to map the selectivity of progesterone encapsulation relative to other steroids.^45−49^

### Light-Powered Progesterone Release

The photoisomerization of **1** allowed progesterone release and uptake to be controlled using light as an external stimulus. When progesterone⊂*trans*-**1** was irradiated with 350 nm light, the *trans*-azobenzene moieties at the vertices of the cage photoisomerized to the *cis* isomer, promoting the dissociation of the cage as evidenced by ^1^H NMR spectra (Figure S49) and releasing the progesterone cargo as observed in the ^1^H DOSY spectrum (Figure S50). Conversely, upon irradiation with 500 nm light, cage *trans*-**1** reformed, resulting in the reuptake of progesterone, as confirmed by ^1^H NMR (Figure S49).

We hypothesized that cyclopentane, which is immiscible with acetonitrile (Figure 1c), might extract progesterone from acetonitrile following its light-driven release from cage **1**. Cyclopentane is an attractive extractant due to its low boiling point and heat of evaporation, which minimize energy consumption during solvent recycling.^50^ Progesterone⊂*trans*-**1** was added to a 1:1 cyclopentane:acetonitrile biphasic system. The ^1^H NMR spectrum of the cyclopentane phase indicated no progesterone release prior to illumination. Slice-selective ^1^H NMR^42^ spectra of the acetonitrile and cyclopentane layers revealed the partitioning of progesterone⊂*trans*-**1** exclusively into the acetonitrile layer (Figure 1c), with no progesterone observed in cyclopentane. The biphasic system was then irradiated at 350 nm to promote progesterone release. The ^1^H NMR signals assigned to encapsulated progesterone in the range −3 to 0 ppm disappeared, suggesting complete progesterone release (Figure S50). After irradiation, slice-selective ^1^H NMR data indicated the presence of free progesterone in both acetonitrile and cyclopentane phases (Figures 1c and S54).

We then attempted the extraction of progesterone from progesterone⊂*trans*-**1** in CD_3_CN before and after irradiation. A 1:1 cyclopentane:acetonitrile biphasic mixture was shaken for 30 min and then left for 10 min to ensure that the two clear layers were well separated. Finally, the cyclopentane phase was removed and evaporated to isolate the progesterone. The partition coefficients before and after illumination were estimated to be 49.5 (±2.10), and 13.7 (±0.17), respectively (see Supporting Information section 6.3). Without irradiation, extractions resulted in only 6% (±1%) of the progesterone being isolated, whereas 78% (±3%) of the progesterone was isolated after irradiation.

### Separation of Progesterone from Other Steroids

**Figure 2:**
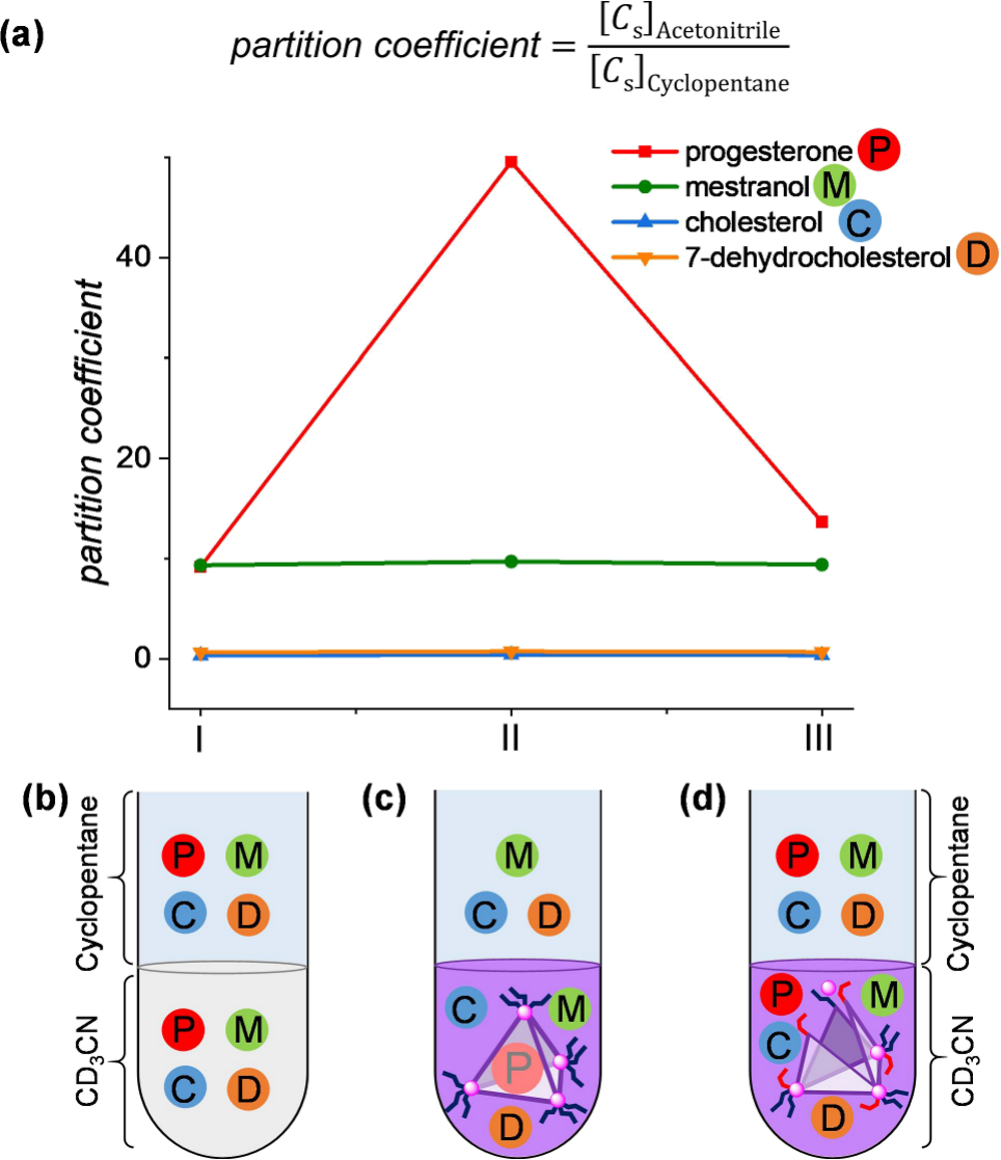
Cage **1** impacts the partition coefficient of progesterone, but not other steroids. (a) Plot showing the distribution of progesterone, mestranol, cholesterol and 7-dehydrocholesterol between acetonitrile and cyclopentane; (b) with no cage (c) in the presence of *trans*-**1** in CD_3_CN; (d) in the presence of disassembled **1** after irradiation with light at 350 nm.

We studied the partitioning of steroids between acetonitrile and cyclopentane (Figure 2 and Supplementary Table 1). As shown in Figure 2a, the presence of *trans*-**1** brought about a sharp increase in the partition coefficient of progesterone from 9 to 50, due to the formation of the host–guest complex progesterone⊂*trans*-**1** (Figure 2c). After illumination at 350 nm, progesterone release was observed, causing the partition coefficient to drop to 14 (Figure 2d). The partition coefficients of the other steroids were minimally impacted by the presence of **1** in either its closed or open state (Figure 2c,d).

**Figure 3:**
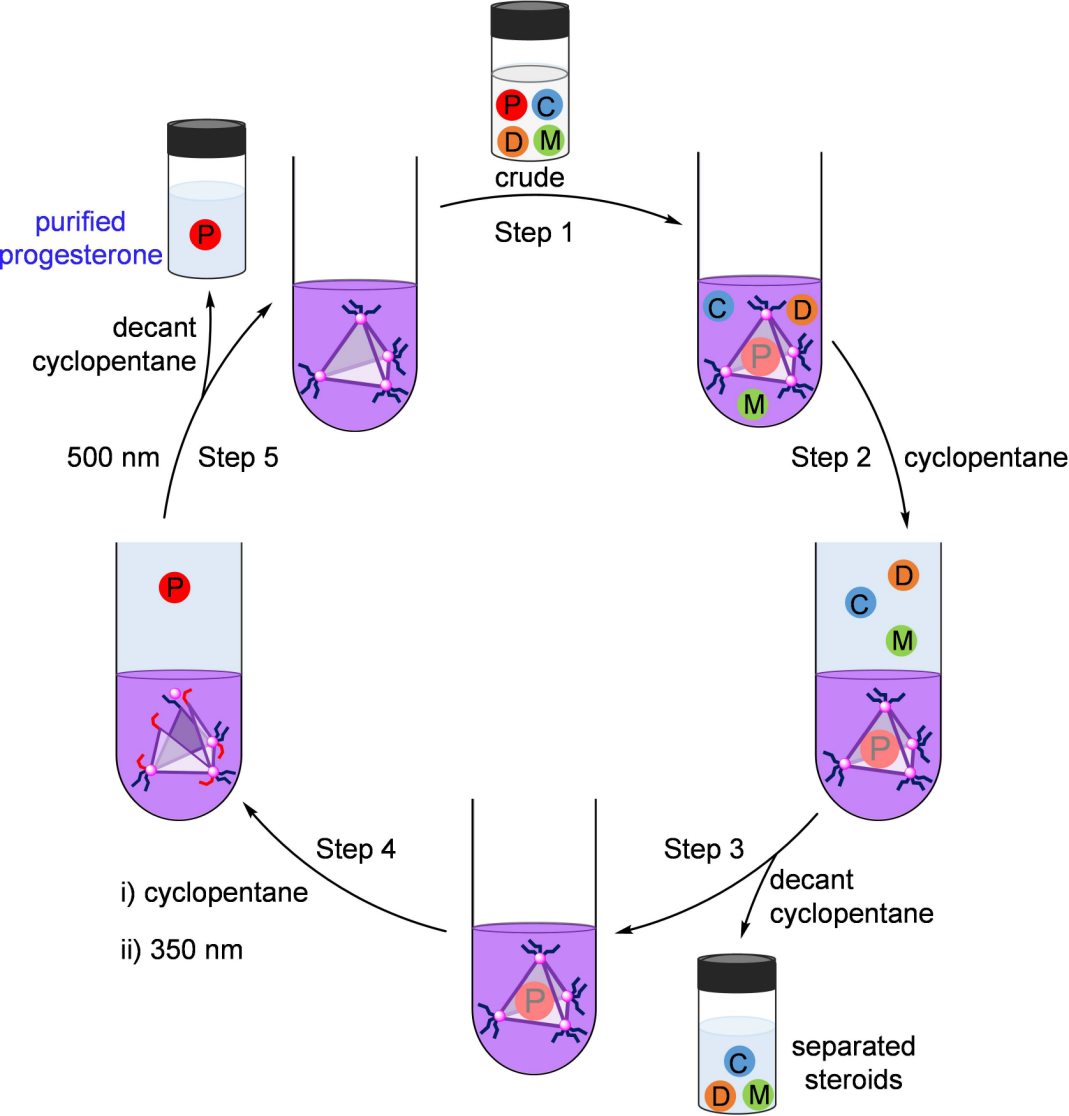
Illustration of stepwise progesterone separation using cage **1**. A mixture of progesterone, mestranol, cholesterol and 7-dehydrocholesterol was initially introduced to a solution of cage *trans*-**1** in CD_3_CN (Step 1). The cage selectively encapsulated progesterone, leaving the other steroids free in solution. Added cyclopentane (Step 2) extracted mestranol, cholesterol and 7-dehydrocholesterol, which were then removed in cyclopentane solution (Step 3). Irradiation at 350 nm resulted in the release of progesterone (Step 4), which was then purified through cyclopentane extraction. Illumination at 500 nm (Step 5) enabled *trans*-**1** to be recycled for further use.

The results of our partitioning experiment suggested a novel means of purifying progesterone from mixtures of chemically similar steroids. This five-step separation system is illustrated in Figure 3. When an equimolar mixture of progesterone, mestranol, cholesterol and 7-dehydrocholesterol was treated with one equivalent of *trans*-**1** in CD_3_CN (Figure 3, Step 1), progesterone⊂*trans*-**1** was observed as the uniquely formed host–guest complex, leaving the other steroids free in solution (Figure S34). Added cyclopentane (Figure 3, Step 2 and Figure S57) extracted the unbound steroids, leaving purified progesterone⊂*trans*-**1** in the acetonitrile phase. Decanting the cyclopentane (Figure 3, Step 3) thus allowed isolation of these steroids. Adding a fresh cyclopentane layer to the progesterone⊂*trans*-**1** in acetonitrile, and then illuminating at 350 nm (Figure 3, Step 4), then decanting and isolating the purified progesterone (Figure 3, Step 5) and illuminating at 500 nm enabled the system to reset, preparing it for the next purification cycle.

We monitored this process using slice-selective ^1^H NMR, which showed distribution of unbound mestranol, cholesterol and 7-dehydrocholesterol across both phases within the biphasic NMR samples (Figure S58). In keeping with the procedure mentioned above, progesterone was observed to transport from acetonitrile to the cyclopentane phase following its release from progesterone⊂*trans*-**1** upon irradiating the system at 350 nm. Recovery of 73% (±3%) of the initial progesterone was achieved from the mixture following five extractions with cyclopentane. Our purification cycle also worked using a mixture containing excess steroids (5 equiv of each steroid with respect to *trans*-**1**; see Supporting Information section 10). By including an extraction step, progesterone was separated from a mixture containing similar steroids, such as testosterone (see Supporting Information section 12). Testosterone binds more strongly within *trans*-**1** than progesterone, thus necessitating the use of cyclopentane extraction to separate testosterone from progesterone, as described in Supporting Information section 15.

## Conclusions

Our work thus demonstrates a straightforward means of light-powered progesterone purification using a metal–organic cage. Many more polytopic aldehyde subcomponents are available in addition to the one studied herein, which have the potential to serve in place of **A** to produce photoresponsive cages with varying shapes, sizes, and guest affinities.^51^ Our technique thus has the potential to form the basis of new and efficient chemical separation processes.

## Supporting Information Available

Experimental procedures; NMR characterizations; mass spectrometry data; volume calculations; ITC experiment (PDF)
