# Supplementary material for: Light-Driven Purification of Progesterone from Steroid Mixtures Using a Photoresponsive Metal–Organic Capsule
Source: J Am Chem Soc. 2024 Jan 17;146(4):2568–73. doi: 10.1021/jacs.3c11005 (PMC10835723; doi:10.1021/jacs.3c11005)
Supplement: Supplementary file 1 — ja3c11005_si_001.pdf [file ja3c11005_si_001.pdf]

Supplementary information for

Light-Driven Purification of Progesterone from Steroid Mixtures Using a Photoresponsive Metal-Organic Capsule

Amit Ghosh, Jiratheep Pruchyathamkorn, Carles Fuertes Espinosa, Jonathan R. Nitschke*

[1]University of Cambridge, Yusuf Hamied Department of Department of Chemistry, Cambridge CB2 1EW, UK.

*Correspondence to: jrn34@cam.ac.uk.

Table of Contents

1. Materials and Methods 1
2. Self-Assembly and Characterization of *trans*-**1** 2
   2.1. Preparation of *trans*-**1** 2
   2.2. Characterization of *trans*-**1** Using NMR Spectrometry 3
   2.3. Characterization of *trans*-**1** Using Mass Spectrometry 7
3. Characterization of Disassembly and Assembly of *trans*-1 Following Exposure to Light at 350 nm and then at 500 nm 8
4. Host-Guest Studies Using *trans*-**1** 10
   4.1. Progesterone as Guest 10
      4.1.1 Binding of Progesterone by *trans*-1 10
      4.1.2 Characterization of Progesterone⊂*trans*-**1** Using NMR 11
      4.1.3. Characterization of Progesterone⊂*trans*-**1** Using Mass Spectrometry 13
      4.1.4. NMR Titration 14
      4.1.5. Variable Temperature Studies 15
      4.1.6. Progesterone Binding Studies Using ITC 16
   4.2. Binding of Other Steroids by *trans*-**1** 17
      4.2.1. Cholesterol as Guest 18
      4.2.2. 7-Dehydrocholesterol as Guest 19
      4.2.3. Mestranol as Guest 20
5. Binding of Progesterone by *trans*-**1** in the Presence of Other Steroids 21
6. Solubility of Steroids in Acetonitrile and Cyclopentane 24
   6.1. ^{1}H NMR of Steroids in Cyclopentane 24
   6.2. ^{1}H NMR of Steroids in CD_3CN 25
   6.3. Determination of Partition Coefficients 25
7. Extraction and Washing 29
   7.1. Washing with Cyclopentane to Remove Excess Progesterone 29
   7.2. Washing with Cyclopentane to Remove Unbound Cholesterol 30
   7.3. Washing with Cyclopentane to Remove Unbound 7-Dehydrocholesterol 30
   7.4. Washing with Cyclopentane to Remove Unbound Mestranol 31
   7.5. Washing with Cyclopentane to Remove Unbound and Excess Steroids 31

8. Photoswitching of Progesterone⊂*trans*-**1** Following Exposure to Light at 350 nm and then at 500 nm 32
9. Light-Powered Progesterone Release from *trans*-**1** in Presence of Cyclopentane 33
10. Purification of Progesterone from a Mixture of Steroids Using *trans*-**1**, Cyclopentane and Light 33
11. Purification of Progesterone from a Stoichiometric Mixture of Steroids Using *trans*-**1**, Cyclopentane and Light 37
12. Purification of Progesterone from a Mixture Containing Six Steroids Using *trans*-**1**, Cyclopentane Extraction, and Light 39
13. Volume Calculations 40
14. MM3 Models 41
15. Purification of Progesterone from Testosterone Using *trans*-**1**, and Cyclopentane Extraction 42
16. References 48

1. Materials and Methods

Unless stated otherwise, all the reagents were purchased from commercial sources and used without further purification. Ligand **A** was prepared following literature procedure with slightly modified reaction condition.[1] Compound **B** was synthesized according to reported procedures.[2] Self-assembly reactions were performed in CD_3CN.

Nuclear Magnetic Resonance (NMR)

NMR spectra were recorded using the following NMR spectrometers: Bruker 400 MHz Avance III HD smart probe (^1H, ^{13}C, ^{19}F, 2D NMR), Bruker Avance 500 MHz DCH cryoprobe (^1H, ^{19}F), Bruker Avance 500 MHz TCI cryoprobe (^1H, ^1H-DOSY). Chemical shifts of the NMR spectra are reported relative to $CDCl_3$ (^1H NMR: δ = 7.26 ppm, ^{13}C NMR: δ = 77.0 ppm), and CD_3CN (^1H NMR: δ = 1.94 ppm, ^{13}C NMR: δ = 118.3 and 1.8 ppm). Data for ^1H NMR spectra were reported as follows: chemical shift (ppm), peak shape (s = singlet, d = doublet, dd = doublet of doublets, ddd= doublet of doublets of doublets, t = triplet, m = multiplet), coupling constant (Hz), and integration. Data for ^{13}C NMR and ^{19}F NMR are reported in terms of chemical shift (ppm).

Slice-selective NMR experiments were performed on a Bruker AVIII HD Smart Probe spectrometer.[3] We thank Dr Peter Gierth and the NMR facility at the University of Cambridge for setting up the slice-selective NMR experiments.

Electrospray Ionization Mass Spectrometry (ESI-MS)

High-resolution ESI mass spectra (HRMS) were obtained using Waters Synap system (capillary voltage 2 kV; cone voltage: 40-60V; desolvation temperature 293 K; source temperature 293 K).

Photo Irradiation

The irradiations at 350 nm and 500 nm were performed in-situ placing the NMR tubes inside a Rayonet photochemical chamber reactor (40 cm deep, 25 cm diameter, 16 × 14 W light sources, operating temperature 32 °C).

2. Self-Assembly and Characterization of *trans*-1

2.1. Preparation of *trans*-1

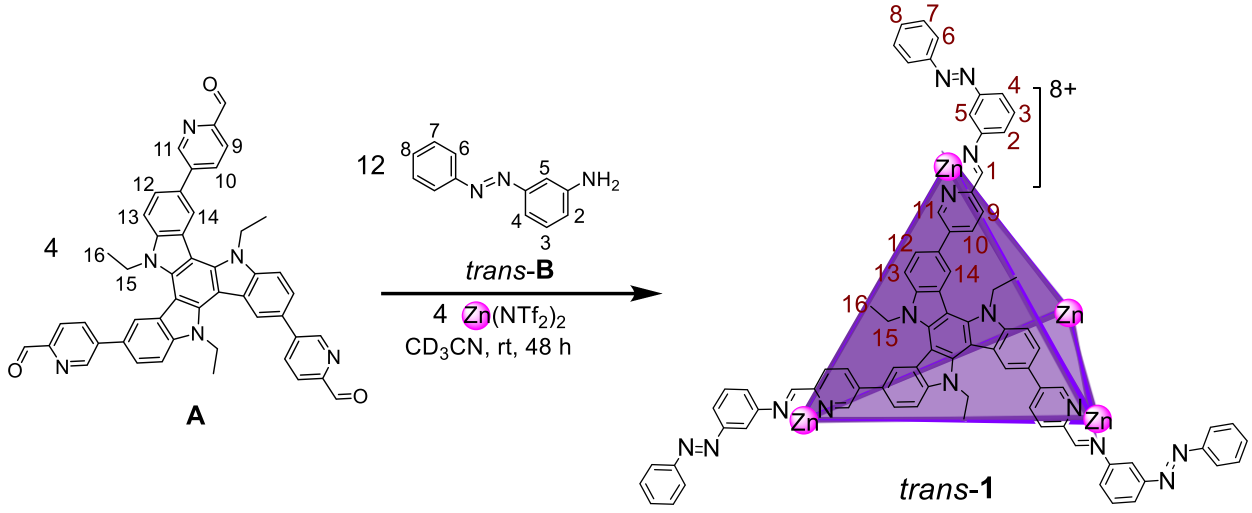

Figure S1: Synthetic scheme for cage *trans*-**1**.

In an NMR tube, ligand **A** (5.00 mg, 6.71 µmol, 4.0 equiv) in 100 µL of $CDCl_3$ was combined with ligand **B** (3.96 mg, 20.1 µmol, 12.0 equiv). To this mixture, $Zn(NTf_2)_2$ (4.20 mg, 6.71 µmol, 4.0 equiv) in 50 µL of CD_3CN was added and subsequently the solvents were evaporated under N_2 flow. Subsequently, 500 µL of CD_3CN was added and the reaction mixture was stirred at room temperature for 48 hours. Diethyl ether (Et_2O) (10 mL) was added to the solution and a large quantity of solid precipitated. The powder was collected by centrifugation and dried under reduced pressure, *trans*-**1** (12.3 mg, 95% yield). ^{1}H NMR (400 MHz, CD_3CN, 25 °C) δ_H (ppm) 9.11 (s, 12H, 1-H), 8.86 (dd, J = 8.4, 0.6 Hz, 12H, 10-H), 8.66 (dd, J = 8.4, 2.6 Hz, 12H, 9-H), 8.46 (dd, J = 2.6, 0.6 Hz, 12H, 11-H), 7.87 (ddd, J = 8.0, 2.4, 1.2 Hz, 12H, 4-H), 7.84 (d, J = 8.8, 12H, 13-H), 7.72 (dd, J = 8.0, 1.2 Hz, 24H, 6-H), 7.64-7.53 (m, 24H, 3-,14-H), 7.51-7.47 (m, 36H, 7-,8-H), 7.43 (dd, J = 8.8, 1.2 Hz, 12H, 12-H), 7.12 (t, J = 2.4 Hz, 12H, 5-H), 7.07 (ddd, J = 8.0, 2.4, 1.2 Hz, 12H, 2-H) 4.89 (q, J = 7.0 Hz, 18H, 15-H); 1.45 (t, J = 7.0 Hz, 36H, 16-H) ppm. ^{13}C NMR (125.8 MHz, CD_3CN): δ 165.0, 152.6, 151.9, 148.1, 144.4, 141.4, 139.4, 138.9, 131.9, 131.4, 130.9, 129.5, 127.6, 124.8, 124.3, 123.0, 122.8, 122.6, 121.5, 120.8, 119.0, 114.5, 111.6, 103.2, 41.8, 15.1 ppm. ^{19}F NMR (CD_3CN, 470.4 MHz, referenced to C_6F_6 at -164.38 ppm): δ -80.5 (s, Tf_2N^-) ppm. ESI-MS: m/z = 673.7 [*trans*-**1**]$^{8+}$, 810.1 ([*trans*-**1**-($C_2F_6NO_4S_2$)]$^{7+}$, 991.9 [*trans*-**1**-2($C_2F_6NO_4S_2$)]$^{6+}$.

2.2. Characterization of *trans*-1 Using NMR Spectrometry

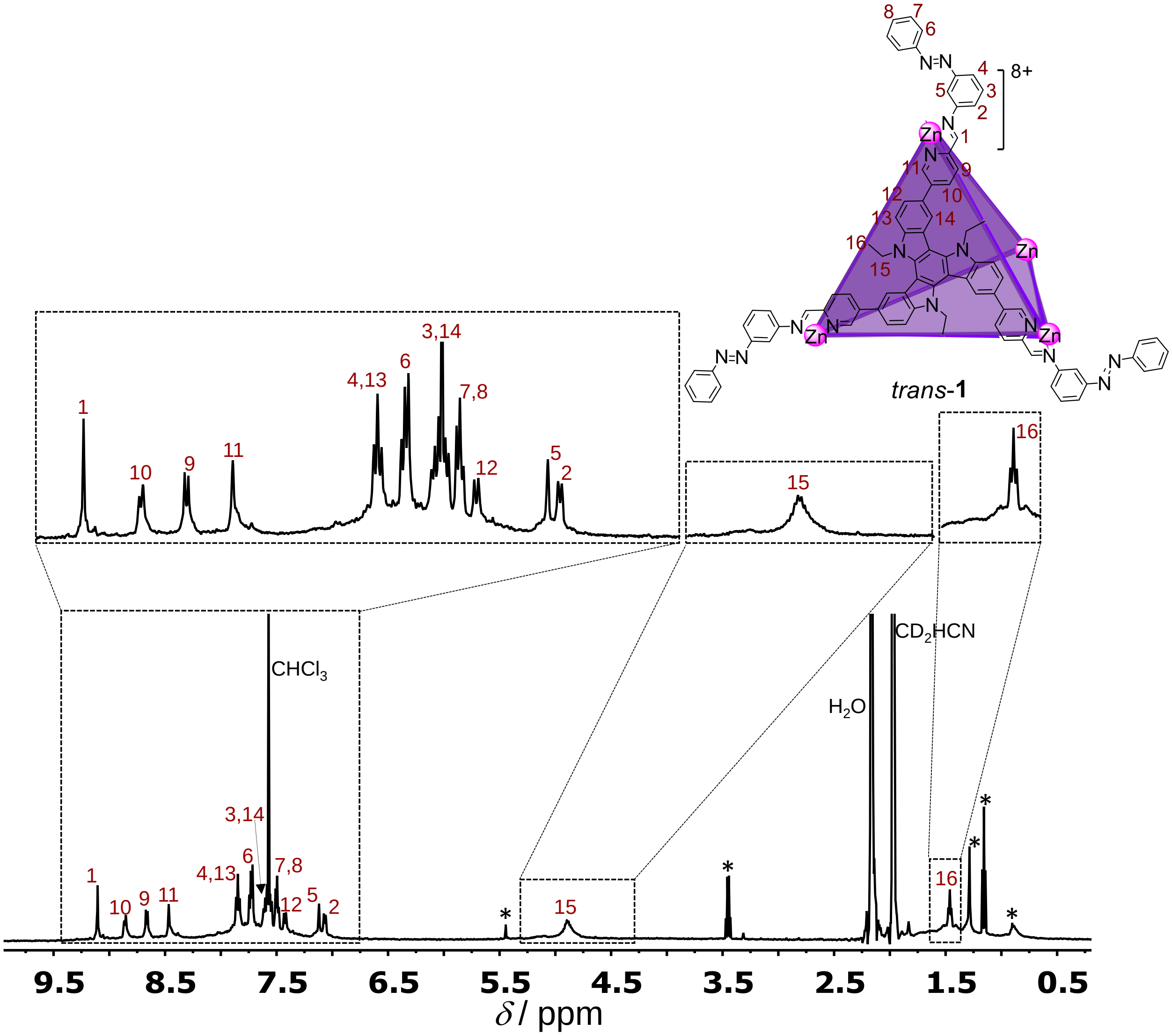

Figure S2: ^{1}H NMR (CD_3CN, 500 MHz, 25 °C) spectrum of *trans*-**1**. The peaks of the solvents (CH_2Cl_2 and Et_2O) and impurities are represented by asterisks.

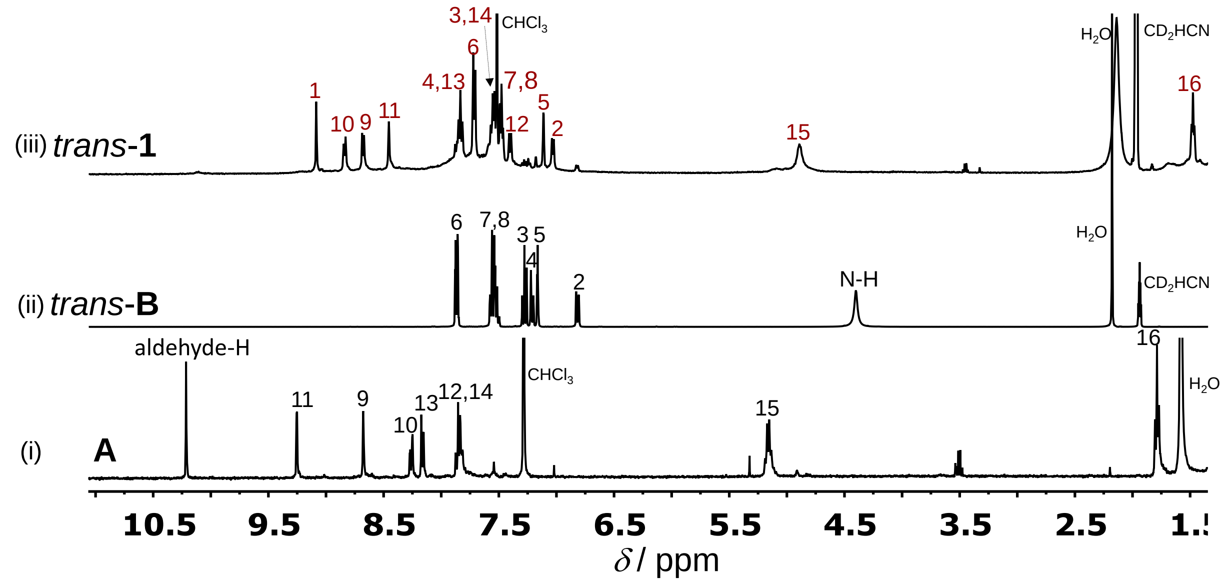

Figure S3: Comparison of ^1H NMR spectra (500 MHz, 25 °C) of (i) subcomponent **A** (in $CDCl_3$); (ii) subcomponent *trans*-**B** (in CD_3CN) and (iii) cage *trans*-**1** (in CD_3CN).

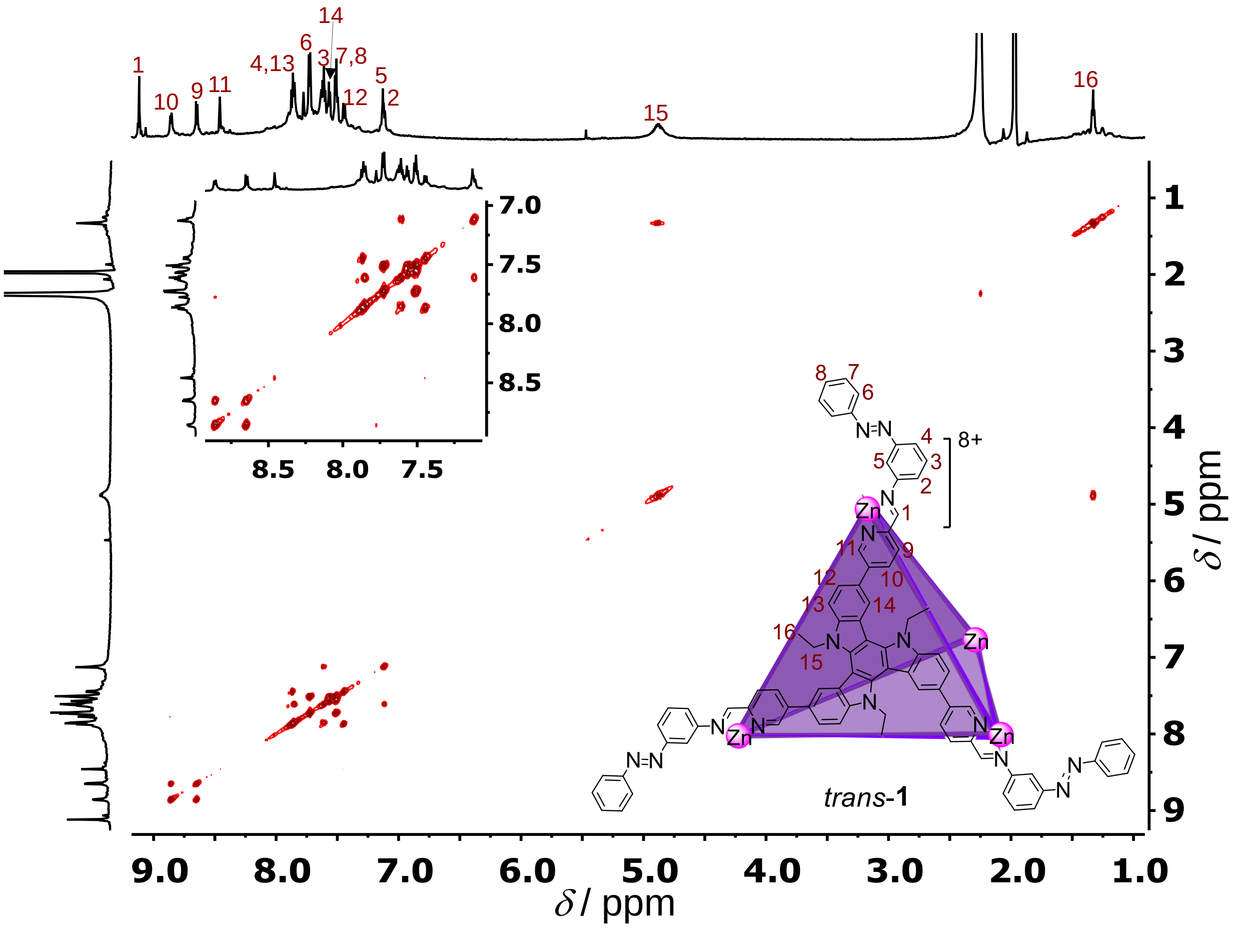

Figure S4: ^1H-^1H COSY NMR spectrum (CD_3CN, 500 MHz, 25 °C) of *trans*-**1**.

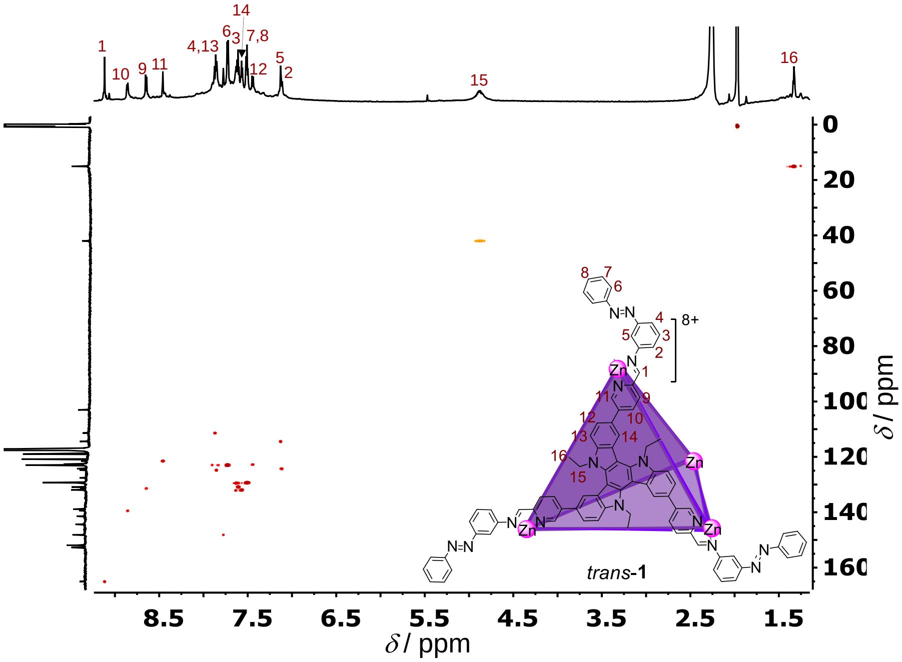

Figure S5: ^{1}H-^{13}C HSQC NMR spectrum (CD$_3$CN, 500 MHz, 25 °C) of *trans*-**1**.

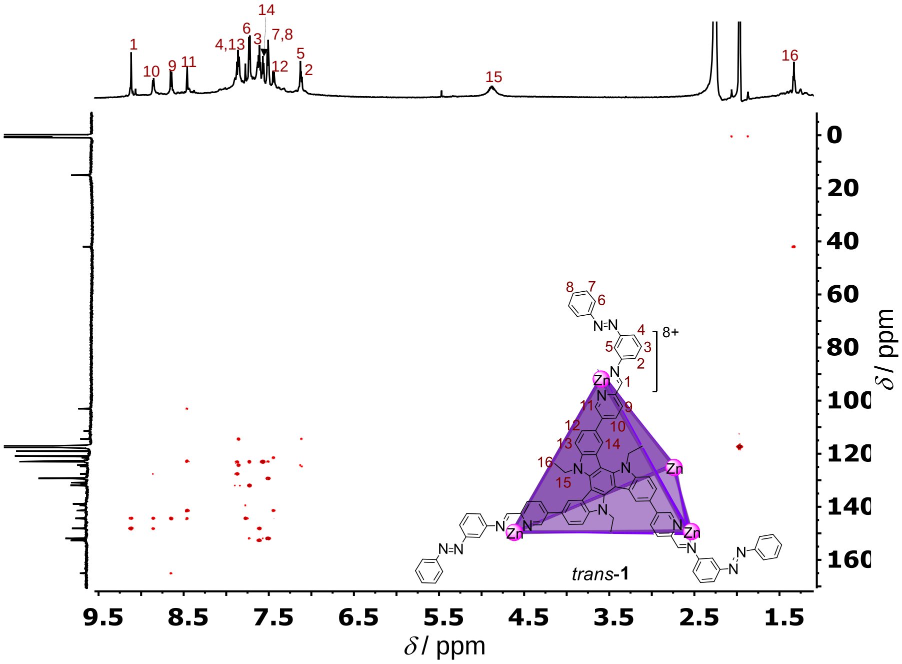

Figure S6: ^{1}H-^{13}C HMBC NMR spectrum (CD$_3$CN, 500 MHz, 25 °C) of *trans*-**1**.

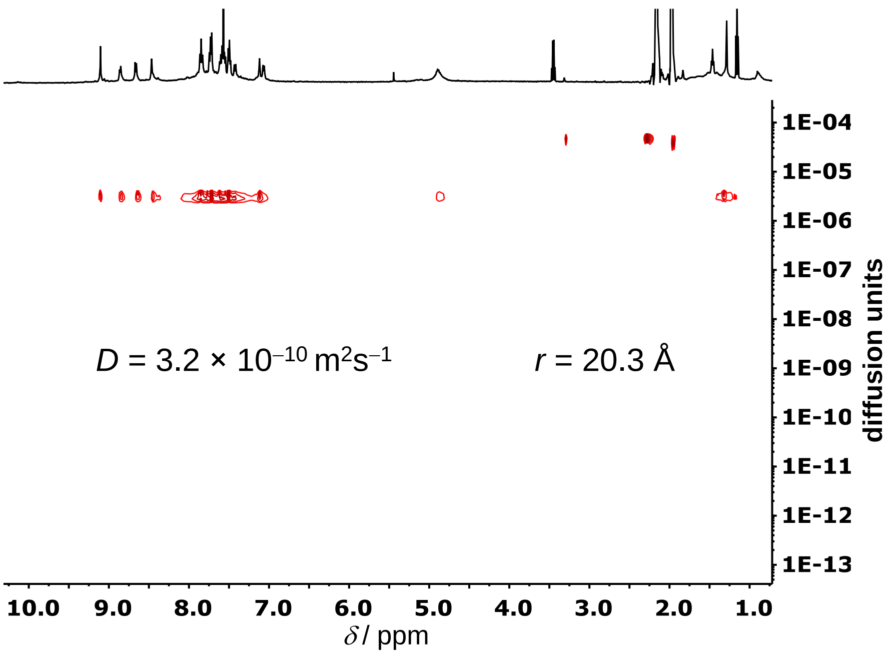

Figure S7: ^{1}H DOSY NMR spectrum (CD_3CN, 400 MHz, 25 °C) of *trans*-**1**.

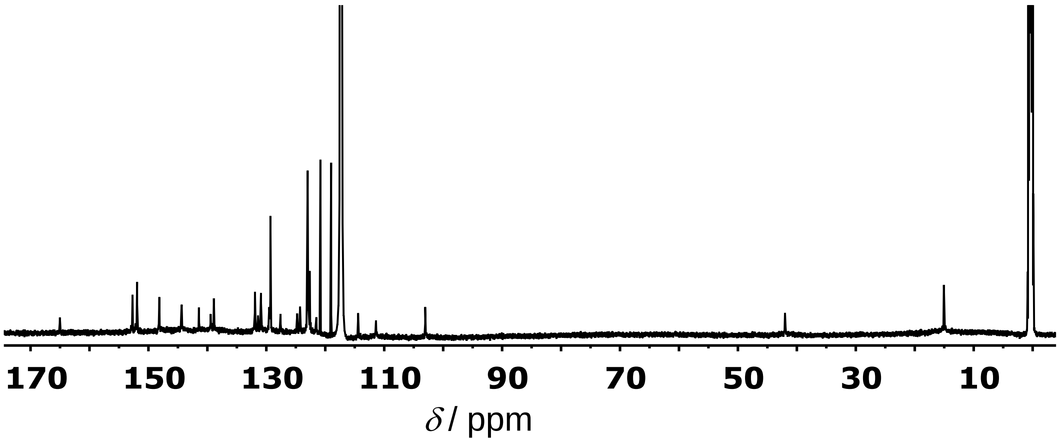

Figure S8: ^{13}C NMR spectrum (CD_3CN, 125.8 MHz, 25 °C) of *trans*-**1**.

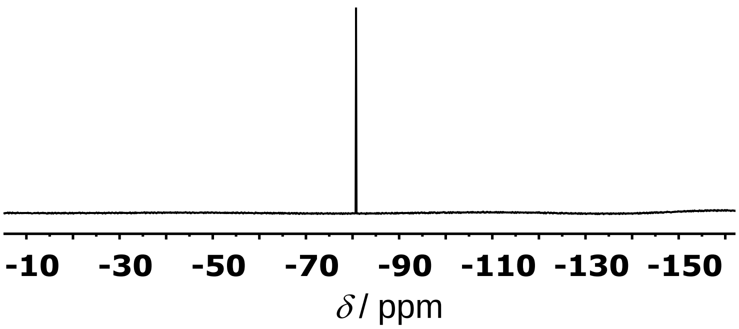

Figure S9: ^{19}F NMR spectrum (CD_3CN, 470.4 MHz, 25 °C) of *trans*-**1**.

2.3. Characterization of *trans*-1 Using Mass Spectrometry

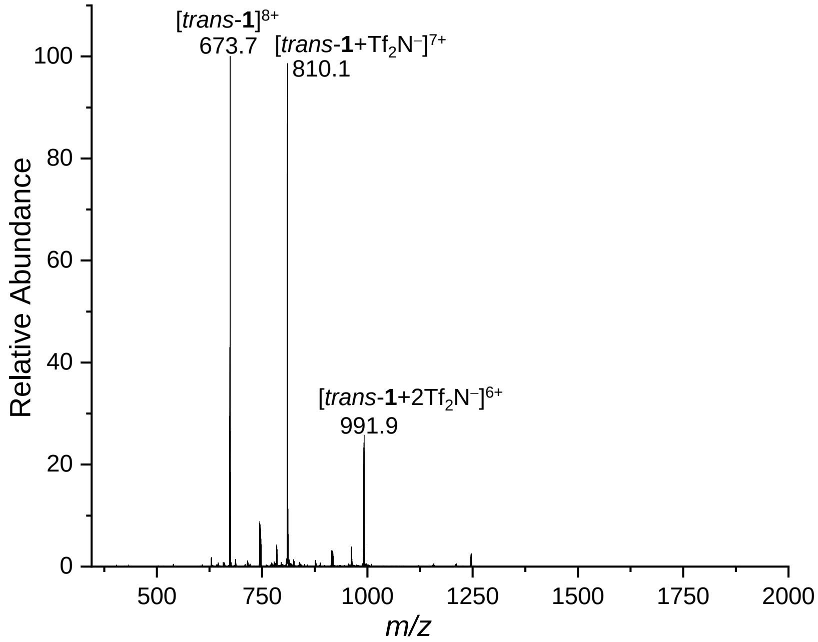

Figure S10: Low-resolution ESI-mass spectrum of *trans*-**1** in CH_3CN.

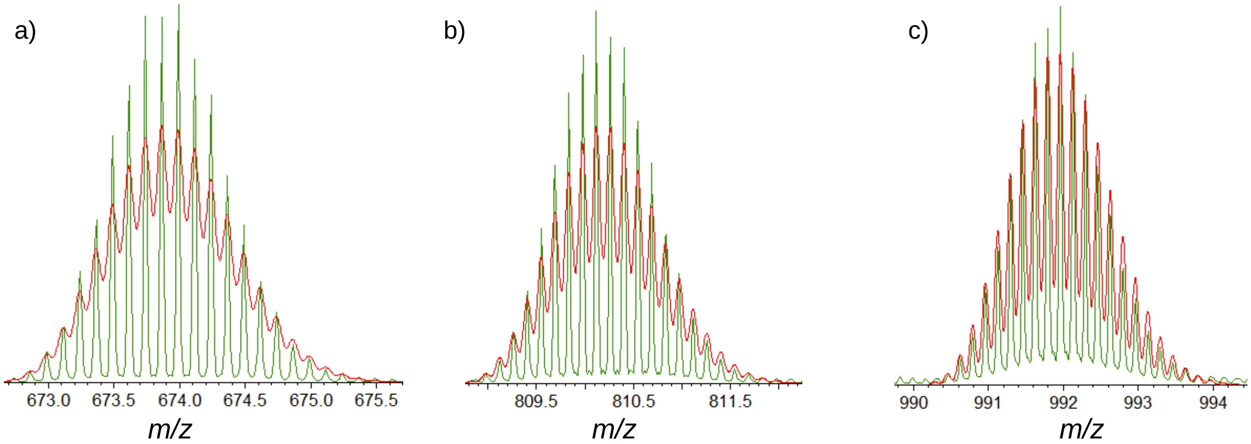

Figure S11: High-resolution ESI-MS spectra of *trans*-**1** in CH_3CN. Experimental (green) and calculated (red) peaks for a) [*trans*-**1**]$^{8+}$: *m/z* = 673.7376 (calculated *m/z* = 673.7330), b) [*trans*-**1**-($C_2F_6NO_4S_2$)]$^{7+}$: *m/z* = 810.1115 (calculated *m/z* = 810.1127), c) [*trans*-**1**-2($C_2F_6NO_4S_2$)]$^{6+}$: *m/z* = 991.9457 (calculated *m/z* = 991.9528).

3. Characterization of Disassembly and Assembly of *trans*-1 Following Exposure to Light at 350 nm and then at 500 nm

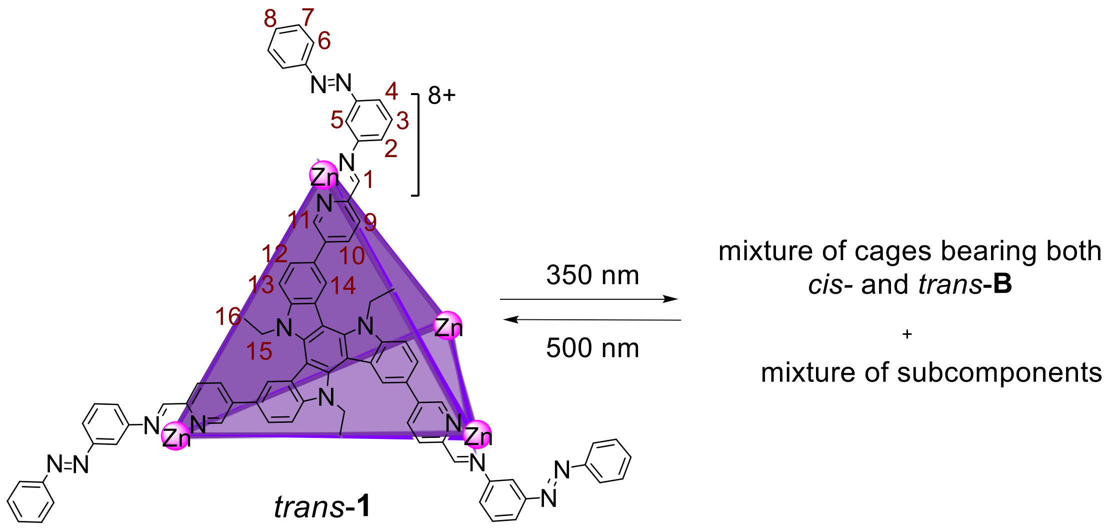

Figure S12: Reversible disassembly and assembly of cage *trans*-**1** powered by light.

Initially *trans*-**1** (c = 2.42 mM) was prepared in an NMR tube as described above and the NMR tube was then irradiated at 350 nm. After 10 minutes the ^{1}H NMR spectrum was recorded. For the reverse process, the same solution was irradiated at 500 nm for 30 minutes and then the NMR spectrum was recorded subsequently. The irradiations were performed in-situ placing the NMR tubes inside a Rayonet photochemical chamber reactor (40 cm deep, 25 cm diameter, 16 × 14 W light sources, operating temperature 32 °C).

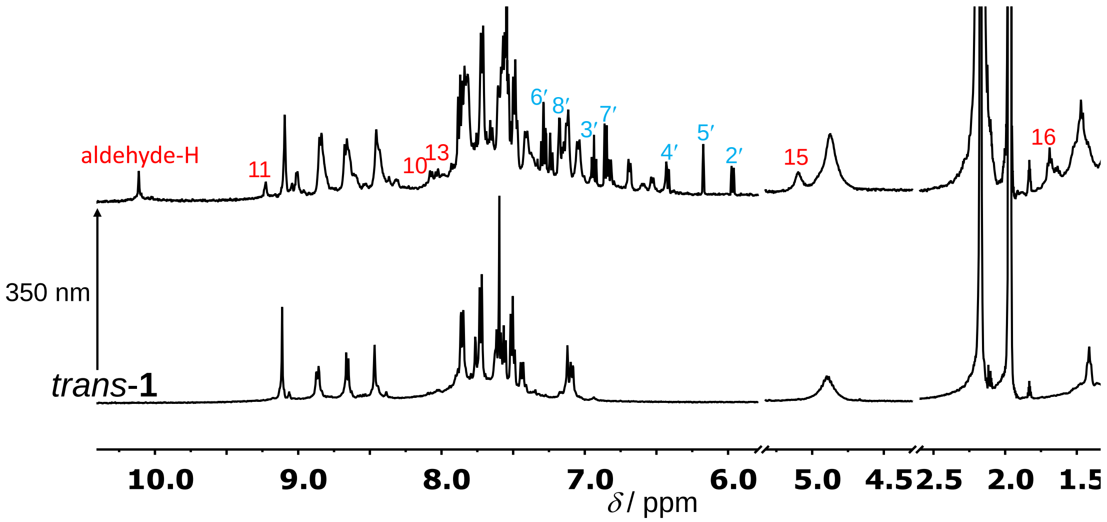

Figure S13: ^{1}H NMR spectra (500 MHz, CD_3CN, 25 °C) of cage *trans*-**1** (c = 2.42 mM) before and after irradiation at 350 nm for 30 minutes. Only the signals for subcomponents **A** (red) and *cis*-**B** (cyan) are marked.

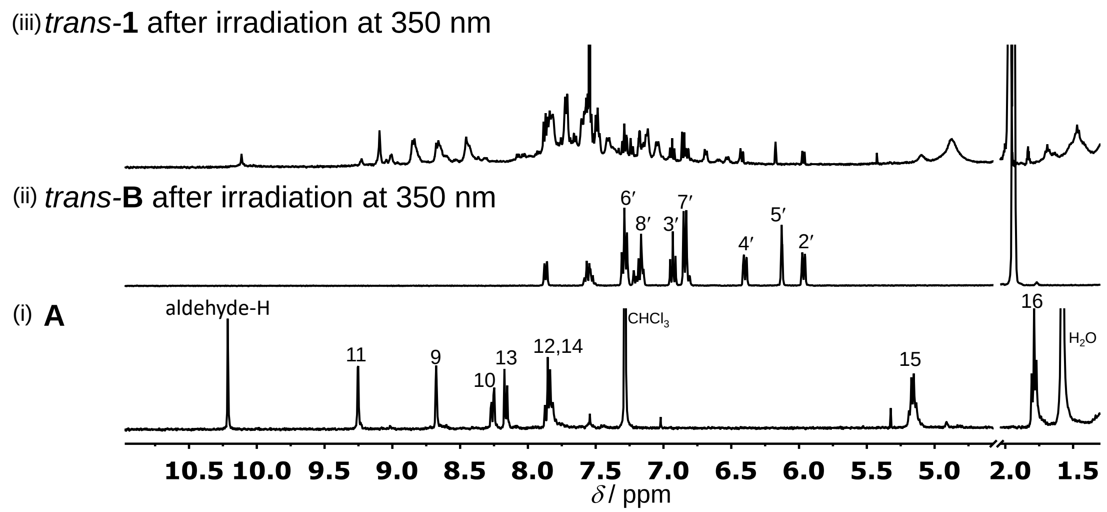

Figure S14: Comparison of ^{1}H NMR spectra (500 MHz, 25 °C) of (i) subcomponent **A** (in $CDCl_3$); (ii) subcomponent **B** (in CD_3CN) after 350 nm irradiation and (iii) cage *trans*-**1** (in CD_3CN) after 350 nm irradiation. The signals of subcomponent **A** and *cis*-**B** are assigned.

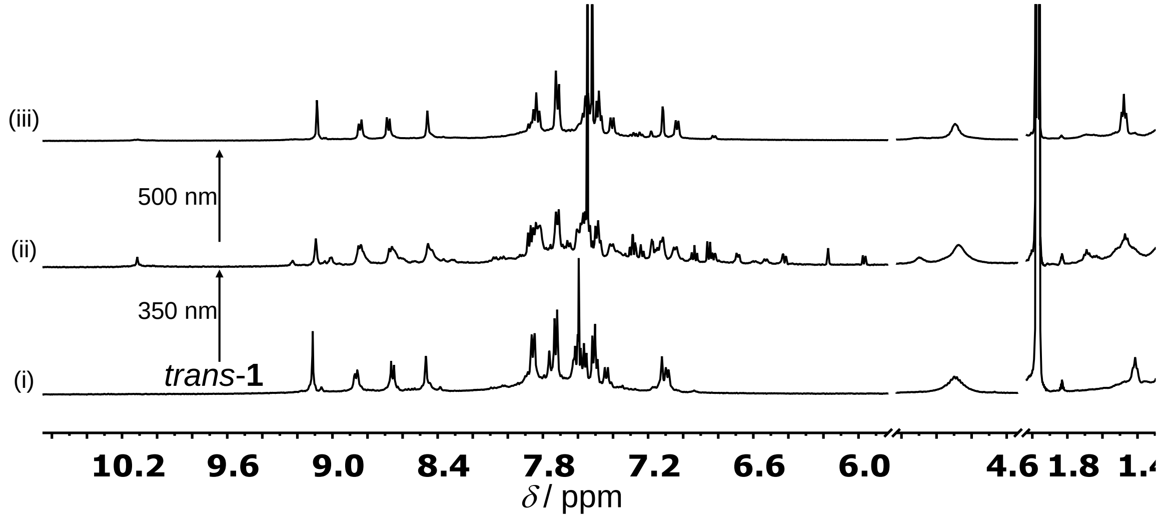

Figure S15: Reversible photoswitching of cage *trans*-**1** (c = 2.42 mM). ^{1}H NMR spectra (500 MHz, CD_3CN, 25 °C) of (i) cage *trans*-**1**; (ii) after 350 nm irradiation for 30 minutes and (iii) after irradiation at 500 nm for 30 minutes.

4. Host-Guest Studies Using *trans*-1

4.1. Progesterone as Guest

4.1.1 Binding of Progesterone by *trans*-1

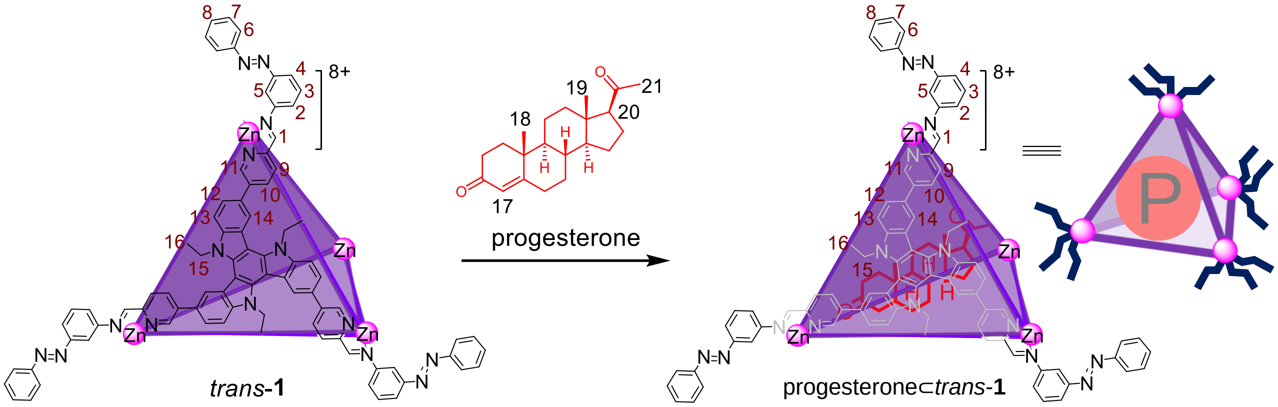

Figure S16: Encapsulation of progesterone as a guest.

Initially *trans*-**1** (c = 0.97 mM) was prepared in a NMR tube as described above. Progesterone was then added progressively, 0.3 equivalent at a time, as a solid. ^{1}H NMR spectra were recorded 30 minutes after each addition. Progesterone was encapsulated by *trans*-**1**, in slow exchange on the ^{1}H NMR chemical shift time scale.

4.1.2 Characterization of Progesterone⊂*trans*-1 Using NMR

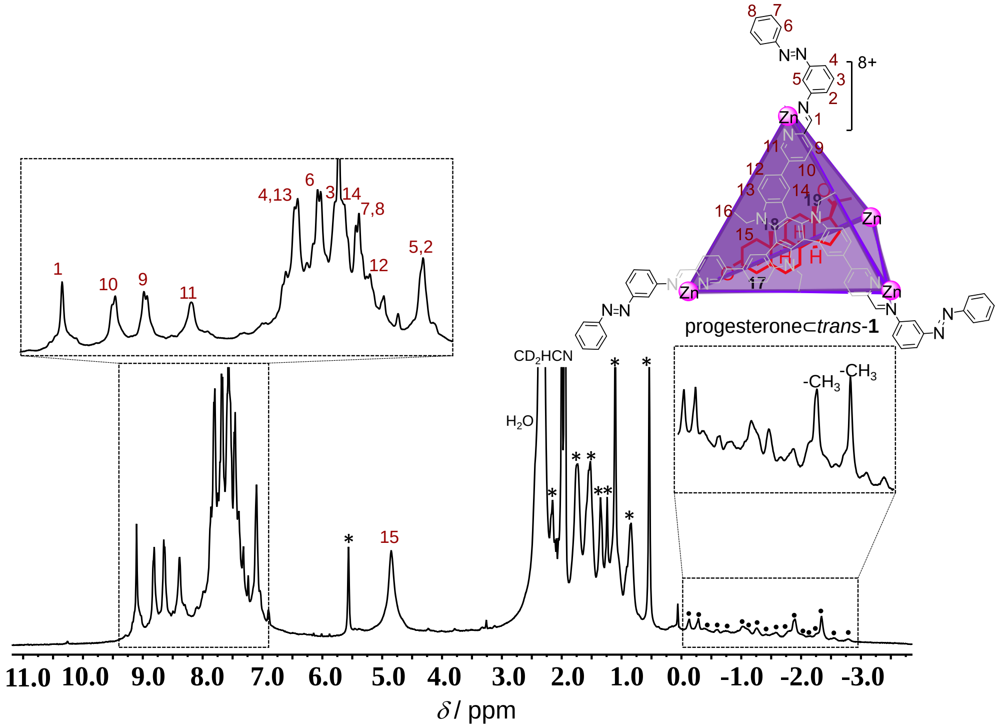

Figure S17: ^{1}H NMR (CD$_3$CN, 500 MHz, 0 °C) spectrum of progesterone⊂*trans*-**1**. Peaks corresponding to unbound progesterone are labelled with an asterisk. The peaks corresponding to encapsulated guests are indicated by filled circles. The methyl peaks of the encapsulated testosterone guest are assigned as indicated.

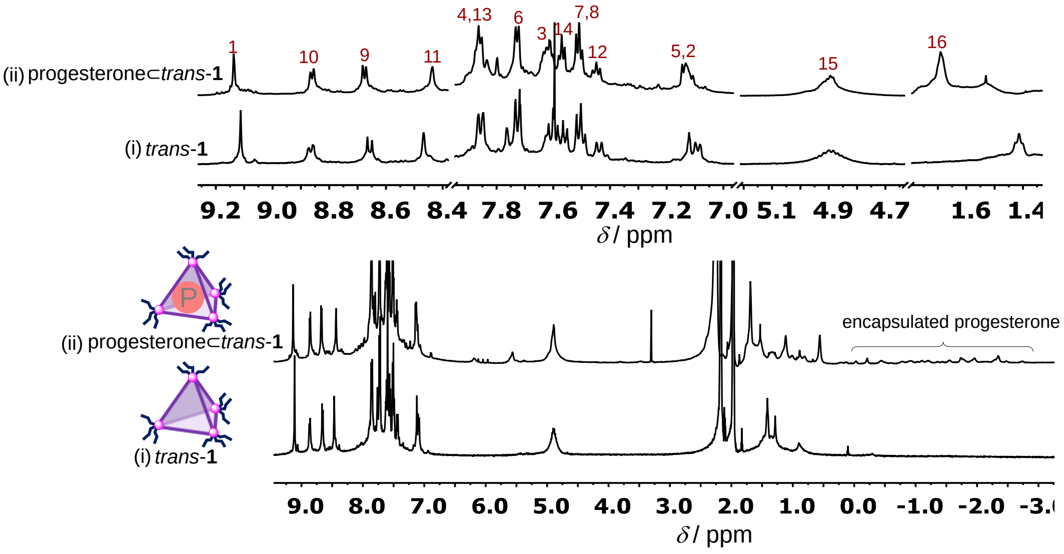

Figure S18: Comparison of ^{1}H NMR spectra (500 MHz, CD$_3$CN, 25 °C) of (i) cage *trans*-**1** and (ii) progesterone⊂*trans*-**1**.

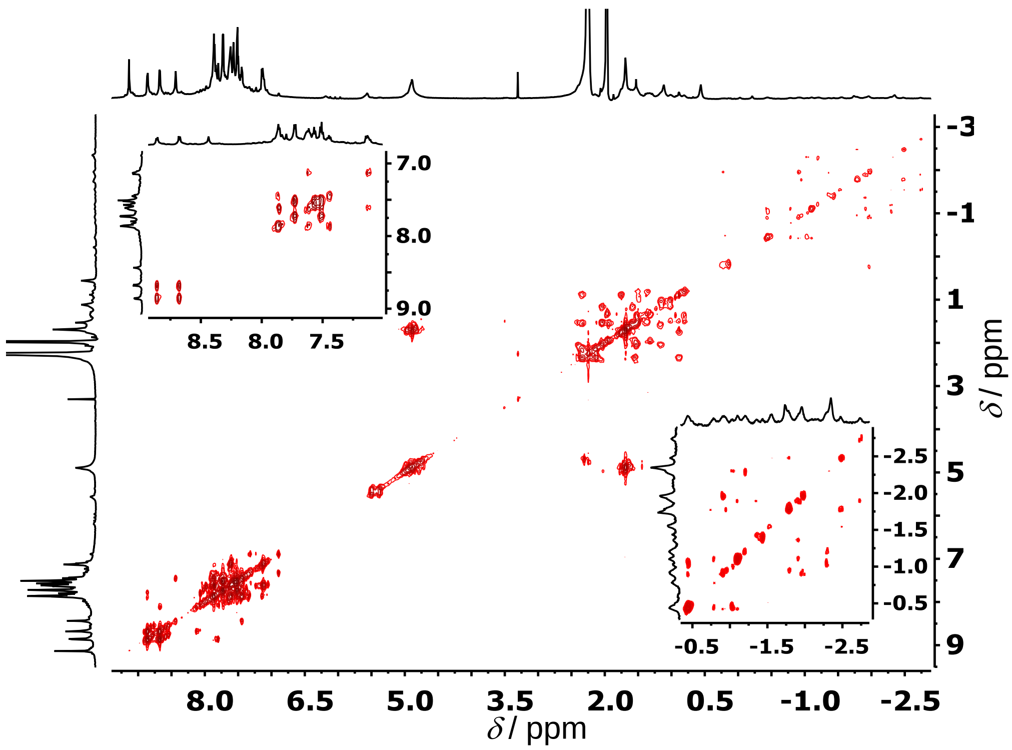

Figure S19: ^{1}H-^{1}H COSY spectrum (CD_3CN, 500 MHz, 25 °C) of progesterone⊂*trans*-**1**.

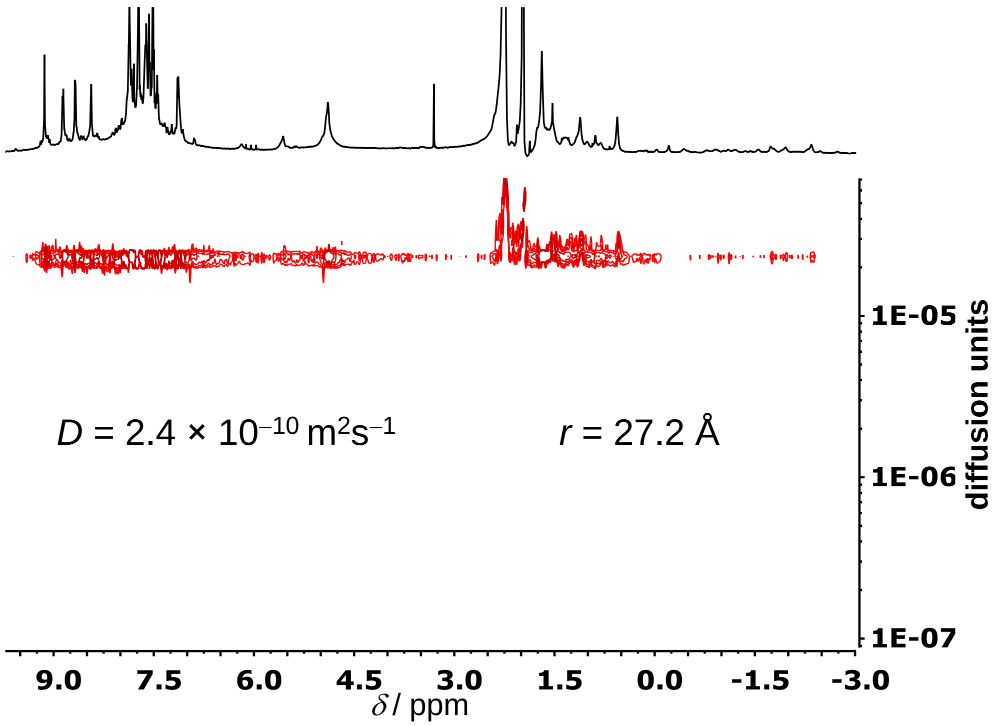

Figure S20: ^{1}H DOSY spectrum (CD_3CN, 400 MHz, 25 °C) of progesterone⊂*trans*-**1**.

4.1.3. Characterization of Progesterone⊂*trans*-1 Using Mass Spectrometry

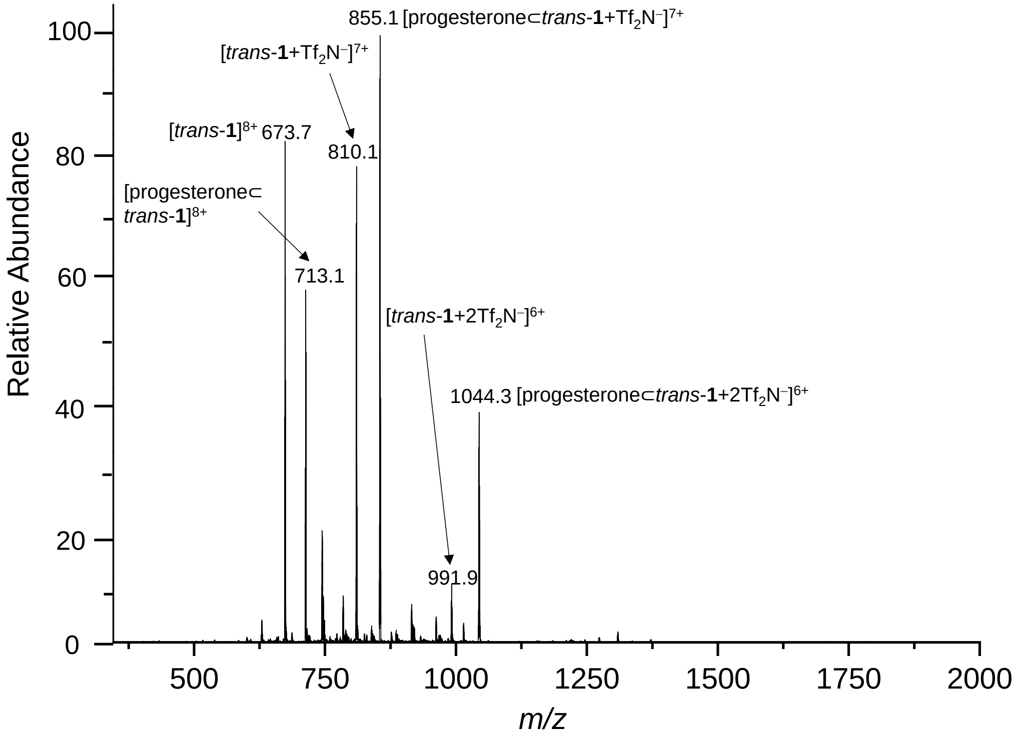

Figure S21: Low-resolution ESI-mass spectrum progesterone⊂*trans*-**1** in CH_3CN.

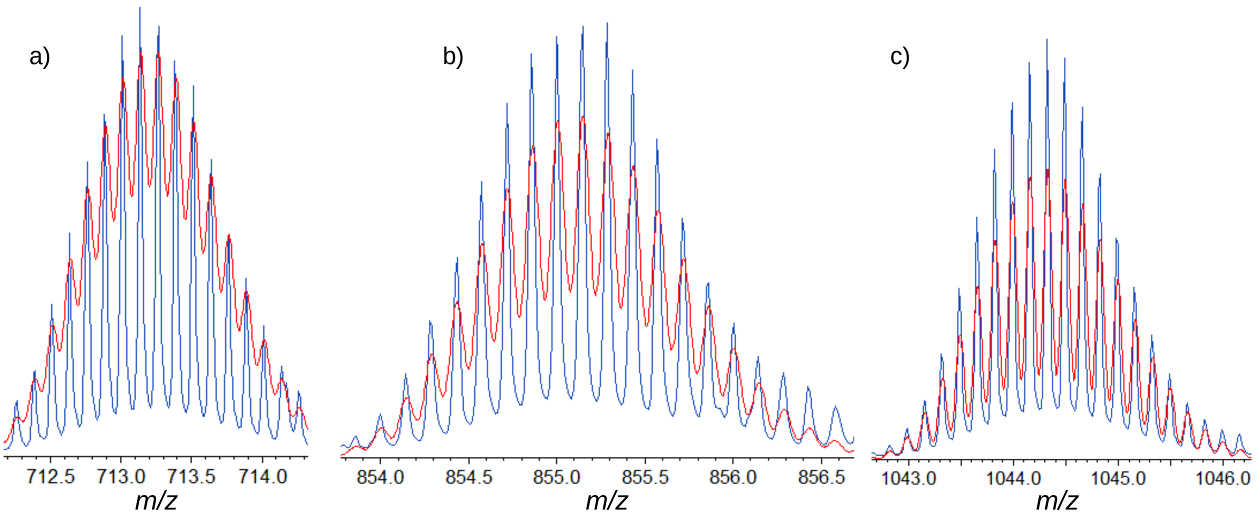

Figure S22: High-resolution ESI-MS spectra of progesterone⊂*trans*-**1** in CH_3CN. Experimental (blue) and calculated (red) peaks for a) [progesterone⊂*trans*-**1**]$^{8+}$: *m/z* = 713.2613 (calculated *m/z* = 713.2567, b) [progesterone⊂*trans*-**1**-($C_2F_6NO_4S_2$)]$^{7+}$: *m/z* = 855.1676 (calculated *m/z* = 855.1718), c) [progesterone⊂*trans*-**1**-2($C_2F_6NO_4S_2$)]$^{6+}$: *m/z* = 1044.4039 (calculated *m/z* = 1044.3919).

4.1.4. NMR Titration

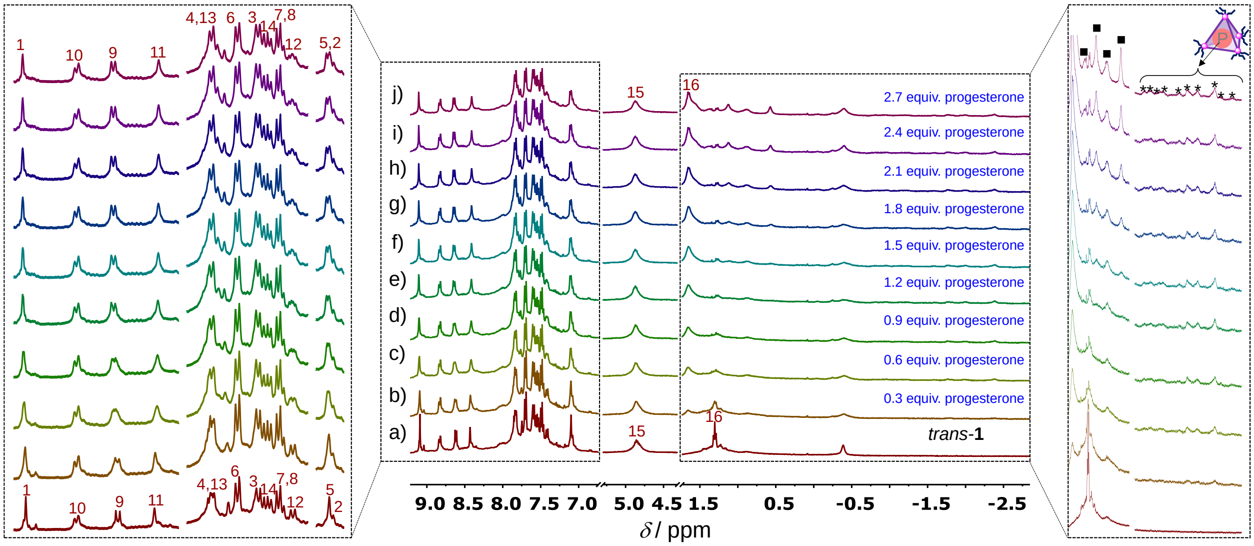

Figure S23: ^{1}H NMR (CD_3CN, 400 MHz, 25 °C) titration of *trans*-**1** (c = 0.97 mM) with a) 0, b) 0.3, c) 0.6, d) 0.9, e) 1.2, f) 1.5, g) 1.8, h) 2.1, i) 2.4 and j) 2.7 equiv. of progesterone. The peaks of the encapsulated progesterone are represented by asterisks *. Peaks corresponding to free progesterone are indicated by squares ■.

4.1.5. Variable Temperature Studies

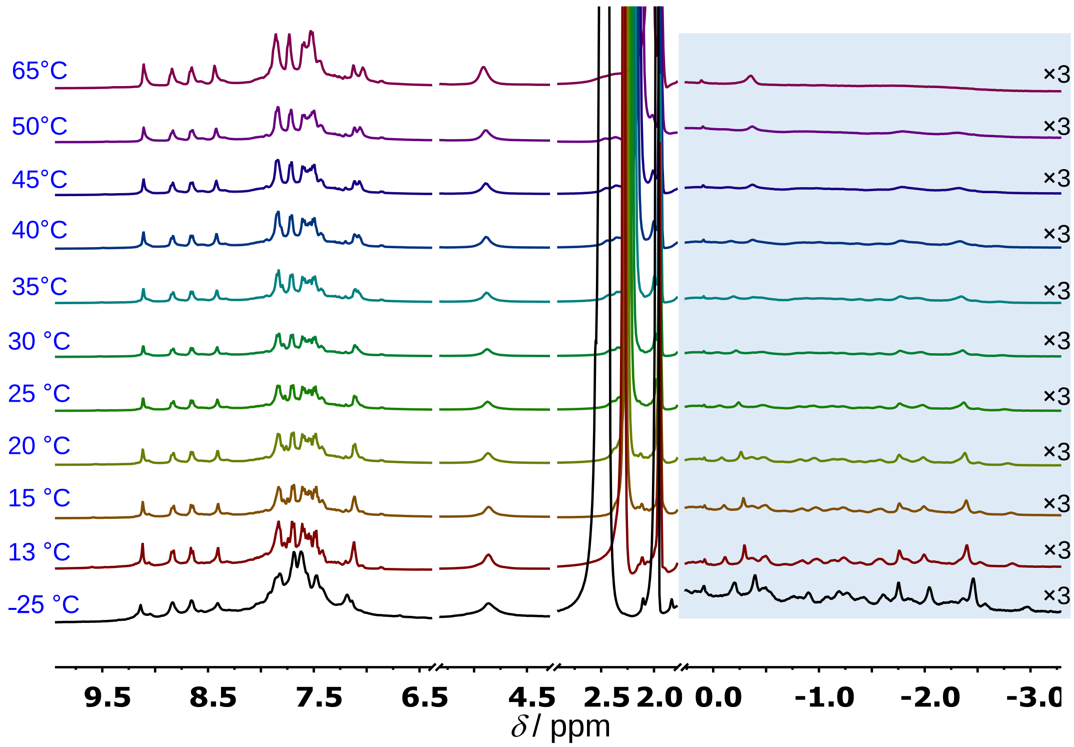

Figure S24: ^{1}H NMR (CD_3CN, 500 MHz, 25 °C) spectra of progesterone⊂*trans*-**1**.

4.1.6. Progesterone Binding Studies Using ITC

ITC experiments were conducted to investigate the thermodynamics of the binding of progesterone within *trans*-**1**. An acetonitrile solution containing 0.06 mM of cage was loaded into the sample cell, and 1-1.2 mM of the guest in acetonitrile was titrated in twenty 2 μL injections of 2 s duration at 120 s intervals. Heats of dilution, measured by titration of the guest into the sample cell containing only solvent, were subtracted from each data set. Progesterone has sufficient solubility to allow accurate determination of the binding data. The data fitting was performed using the Origin software package, using a one set of sites model to model a 1:1 binding event.

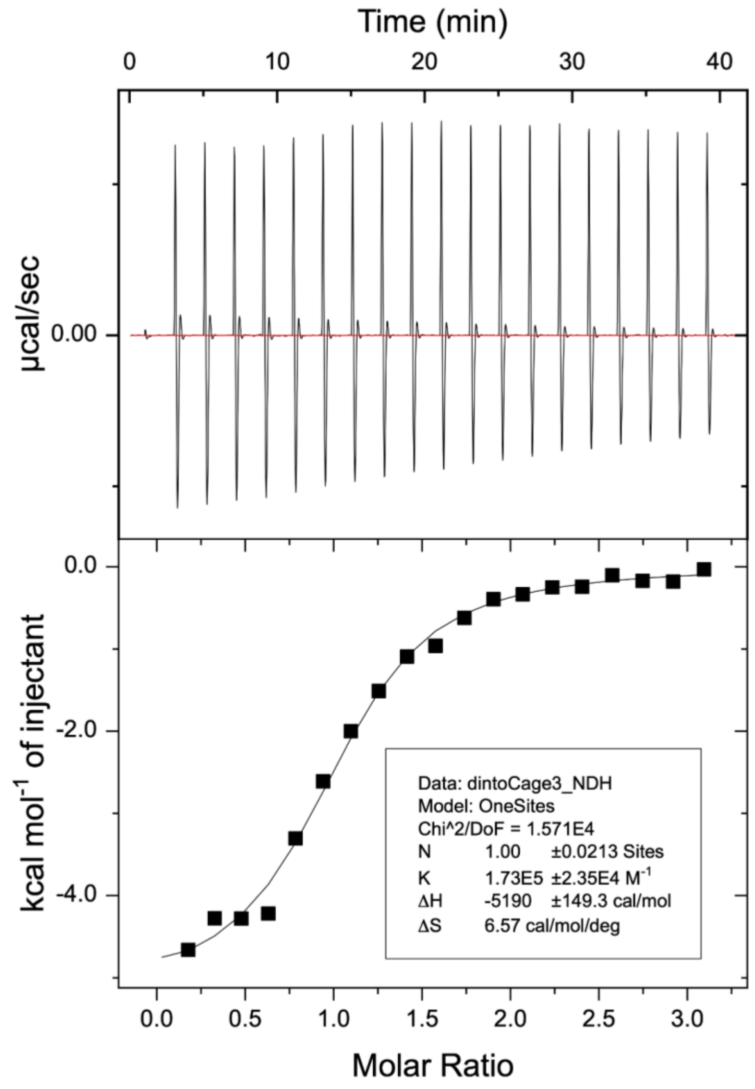

Figure S25: Calorimetric studies of the complexations of progesterone by cage *trans*-**1**. ITC titration plot and fitted curve (25 °C, CH_3CN) obtained through titration of progesterone into *trans*-**1** (0.06 mM), which fit to a 1:1 binding stoichiometry.

4.2. Binding of Other Steroids by *trans*-1

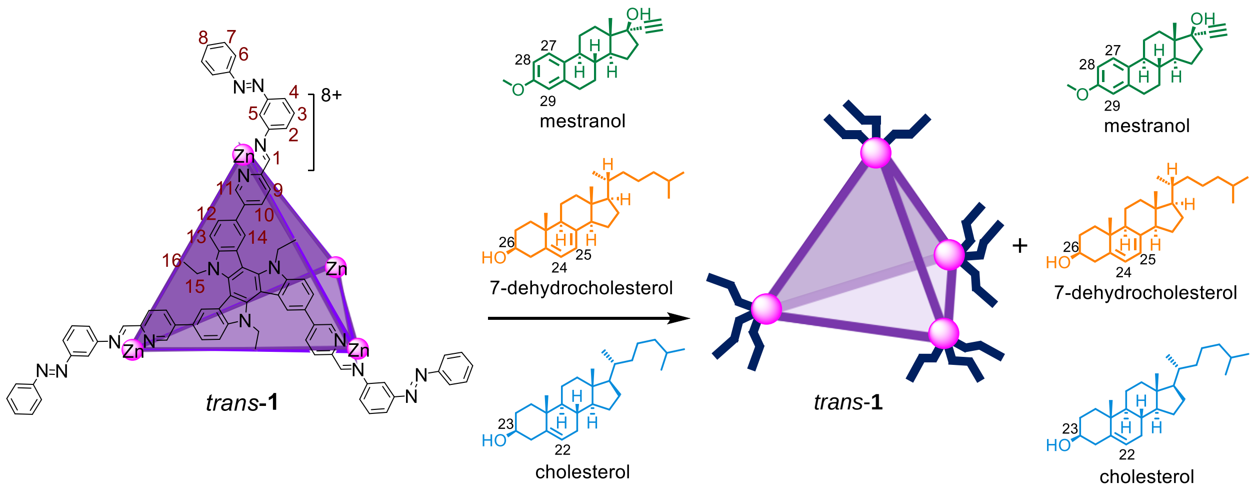

Figure S26: Host-guest studies using other steroids as prospective guests (mestranol, cholesterol and 7-dehydrocholesterol).

The steroids mestranol, cholesterol and 7-dehydrocholesterol were investigated as potential guests for *trans*-**1** (c = 0.97 mM) because the above-mentioned steroids also show good solubility in cyclopentane, as does progesterone. Initially a solution of *trans*-**1** was prepared in a NMR tube as described above. Five equivalents of each steroid was then added, as a solid. ^{1}H NMR spectra were recorded 30 minutes after the addition of each steroid. NMR spectra indicated that the other steroids were not encapsulated by *trans*-**1**.

4.2.1. Cholesterol as Guest

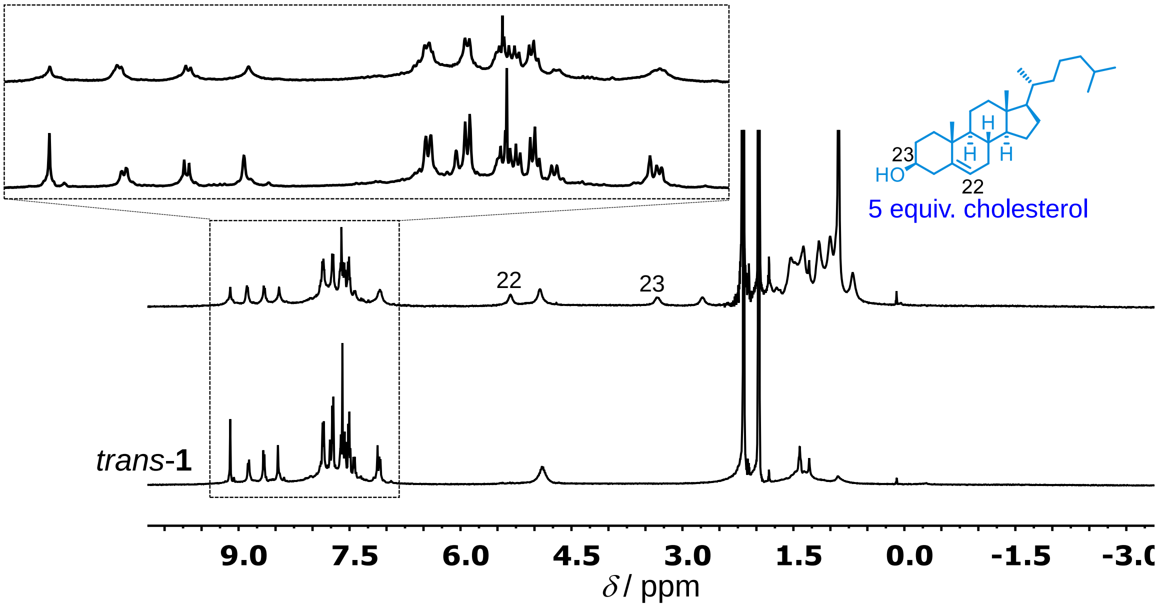

Figure S27: ¹H NMR (CD_3CN, 500 MHz, 25 °C) spectra of *trans*-**1** (*c* = 0.97 mM) in the absence and in the presence of excess cholesterol (*c* = 4.85 mM).

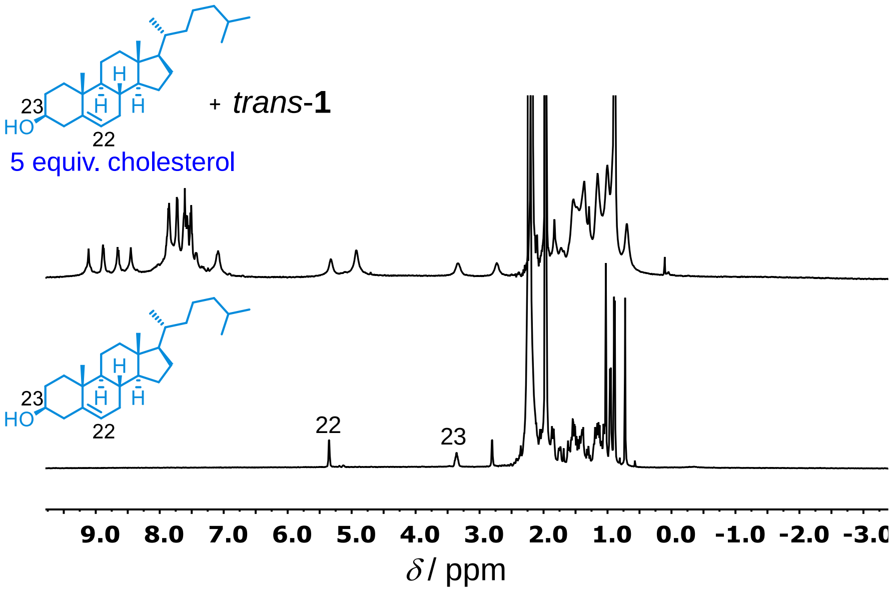

Figure S28: Comparison of ¹H NMR (CD_3CN, 500 MHz, 25 °C) spectra of cholesterol, and cholesterol (*c* = 4.85 mM) in the presence of *trans*-**1** (*c* = 0.97 mM).

4.2.2. 7-Dehydrocholesterol as Guest

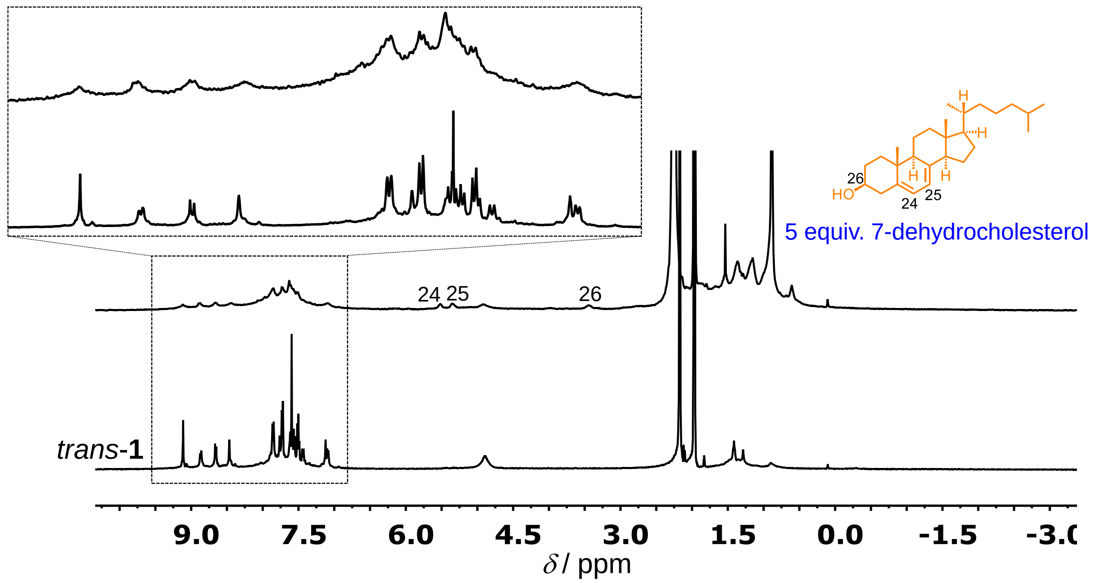

Figure S29: ^{1}H NMR (CD_3CN, 500 MHz, 25 °C) spectra of *trans*-**1** (c = 0.97 mM) in the absence and in the presence of excess 7-dehydrocholesterol (c = 4.85 mM).

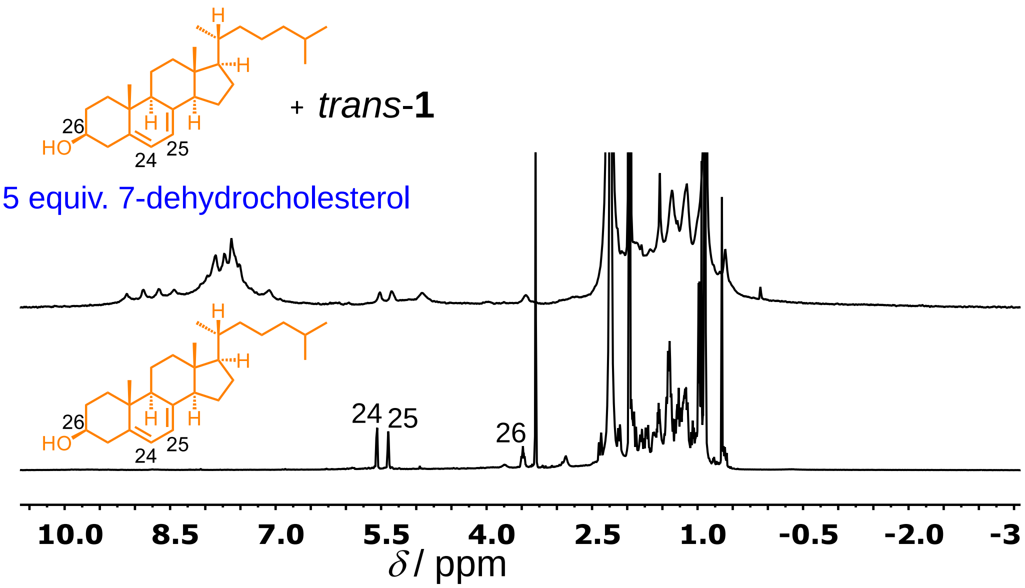

Figure S30: Comparison of ^{1}H NMR (CD_3CN, 500 MHz, 25 °C) spectra of 7-dehydrocholesterol, and 7-dehydrocholesterol (c = 4.85 mM) in the presence of *trans*-**1** (c = 0.97 mM).

4.2.3. Mestranol as Guest

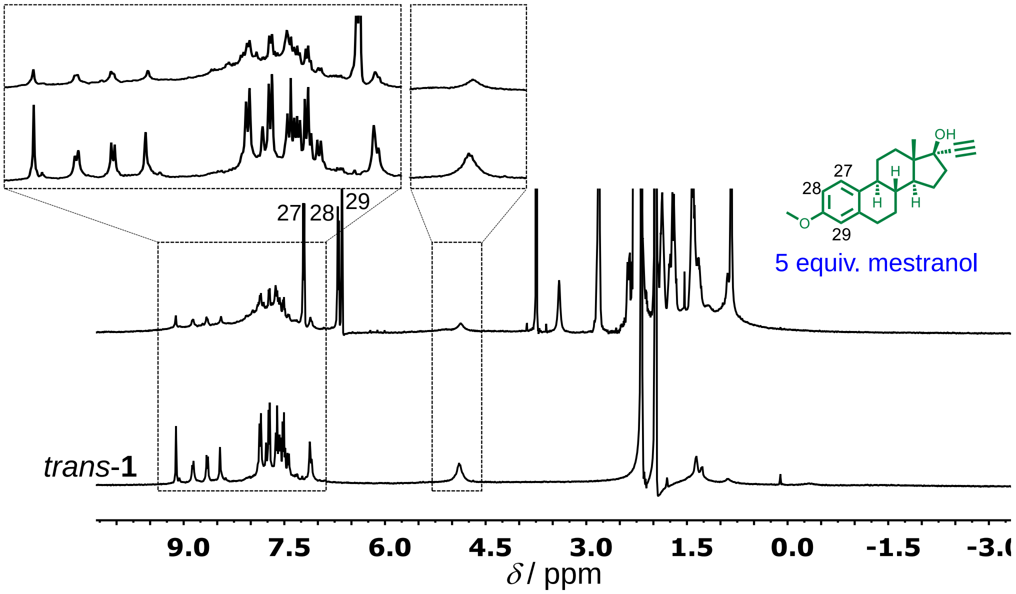

Figure S31: ¹H NMR (CD_3CN, 500 MHz, 25 °C) spectra of *trans*-**1** (*c* = 0.97 mM) in the absence and in the presence of excess mestranol (*c* = 4.85 mM).

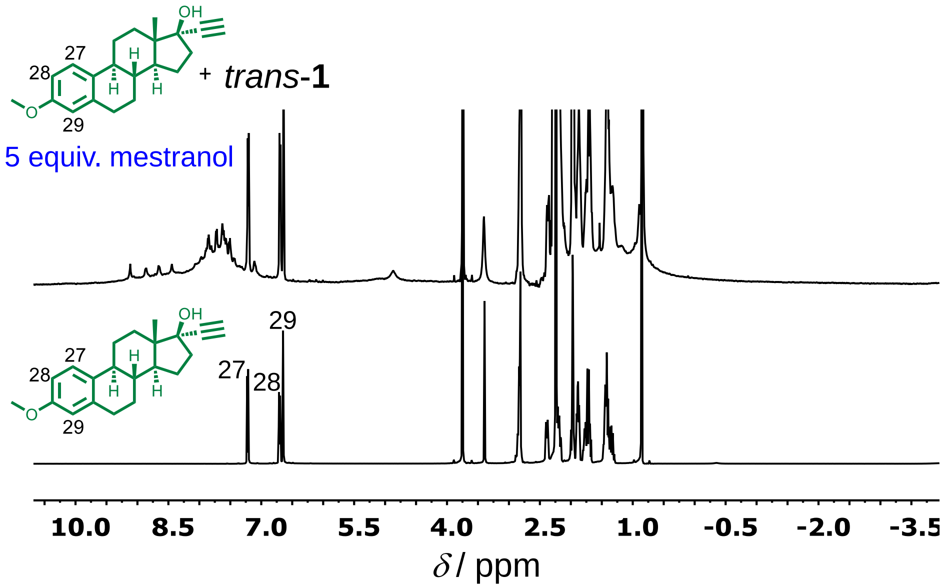

Figure S32: Comparison of ¹H NMR (CD_3CN, 500 MHz, 25 °C) spectra of mestranol, and mestranol (*c* = 4.85 mM) in the presence of *trans*-**1** (*c* = 0.97 mM).

5. Binding of Progesterone by *trans*-1 in the Presence of Other Steroids

Initially *trans*-**1** (c = 0.97 mM) was prepared in a NMR tube as described above. Mestranol (c = 0.97 mM or c = 4.85 mM), progesterone (c = 0.97 mM or c = 4.85 mM), cholesterol (c = 0.97 mM or c = 4.85 mM) and 7-dehydrocholesterol (c = 0.97 mM or c = 4.85 mM) were then added individually. It was found that progesterone was the only steroid encapsulated by *trans*-**1**, showing a slow exchange on the ^{1}H NMR chemical shift time scale while other steroids remain unbound.

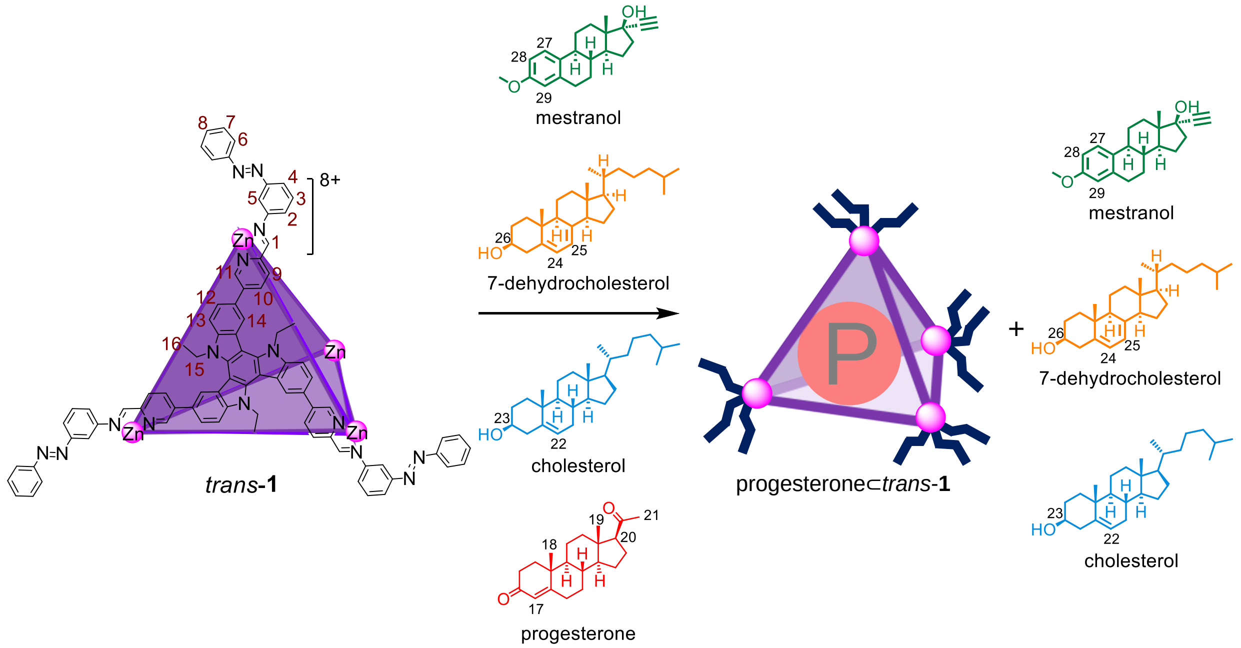

Figure S33: Guest binding studies of *trans*-**1** in the presence of progesterone, mestranol, cholesterol and 7-dehydrocholesterol.

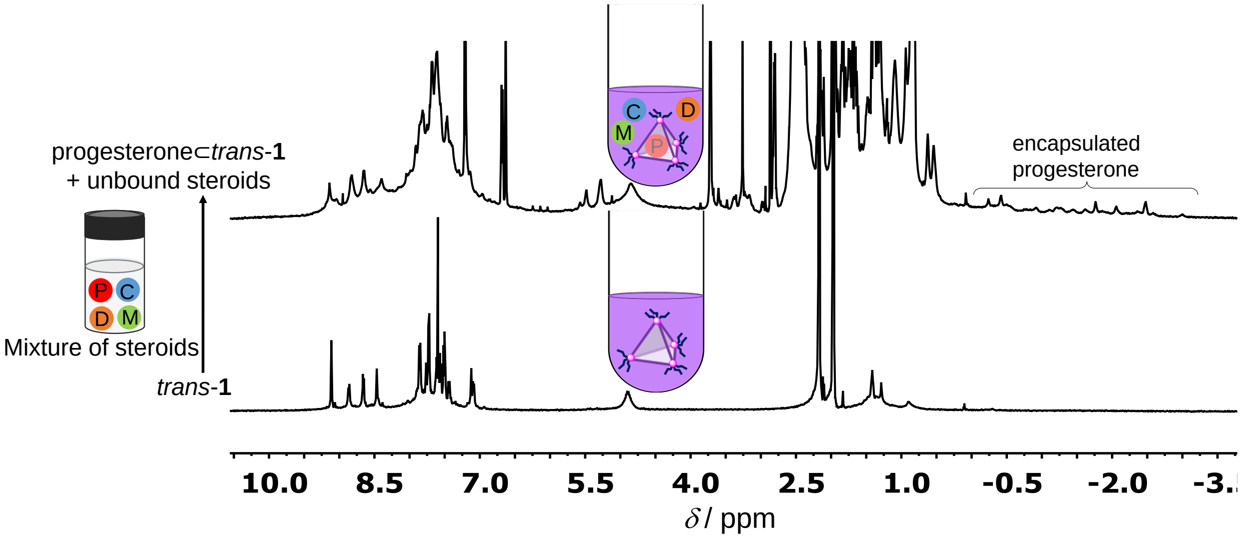

Figure S34: ^{1}H NMR (CD_3CN, 500 MHz, 25 °C) spectra of *trans*-**1**, and *trans*-**1** (*c* = 0.97 mM) in the presence of the mixture of steroids (*c* = 0.97 mM).

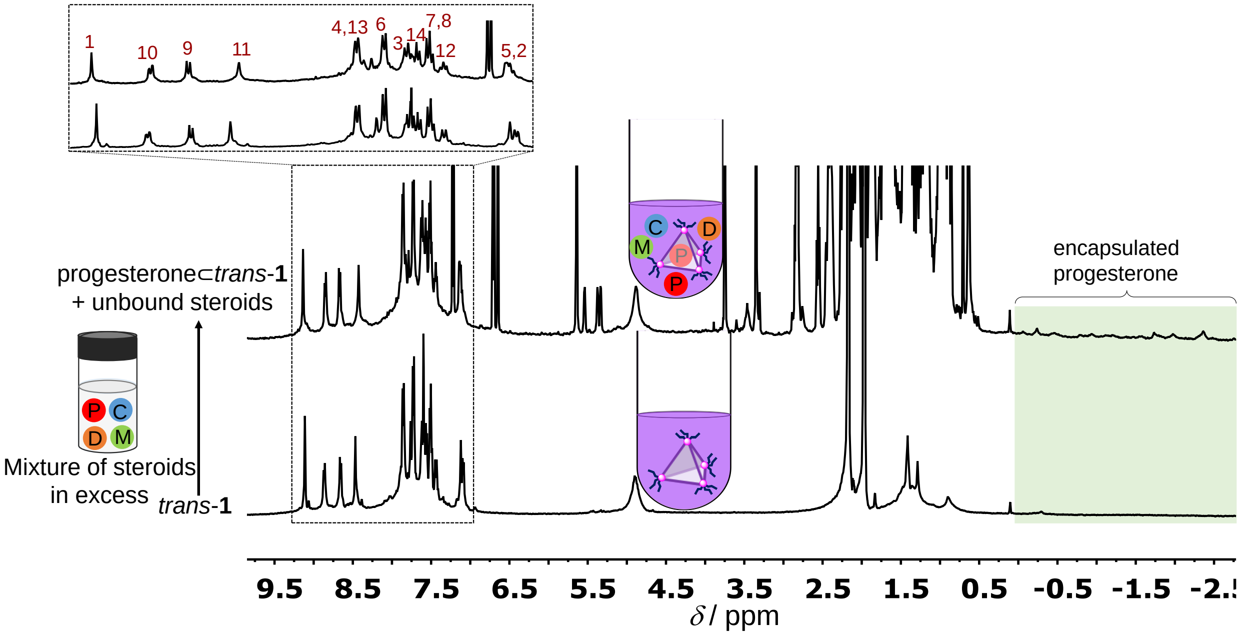

Figure S35: ^{1}H NMR (CD_3CN, 500 MHz, 25 °C) spectra of *trans*-**1** (*c* = 0.97 mM), and *trans*-**1** in the presence of the mixture of steroids (*c* = 4.85 mM). The peaks of the progesterone encapsulated within *trans*-**1** are highlighted.

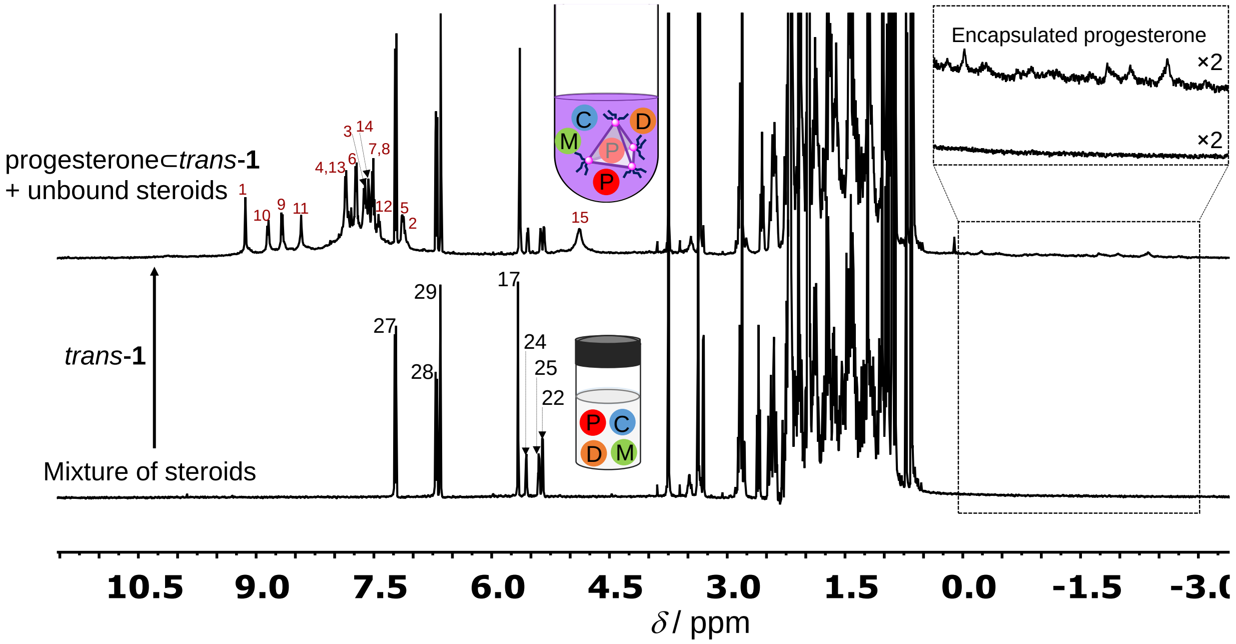

Figure S36: ^{1}H NMR (CD_3CN, 500 MHz, 25 °C) spectra of the mixture of steroids, and the mixture of steroids in the presence of *trans*-**1**.

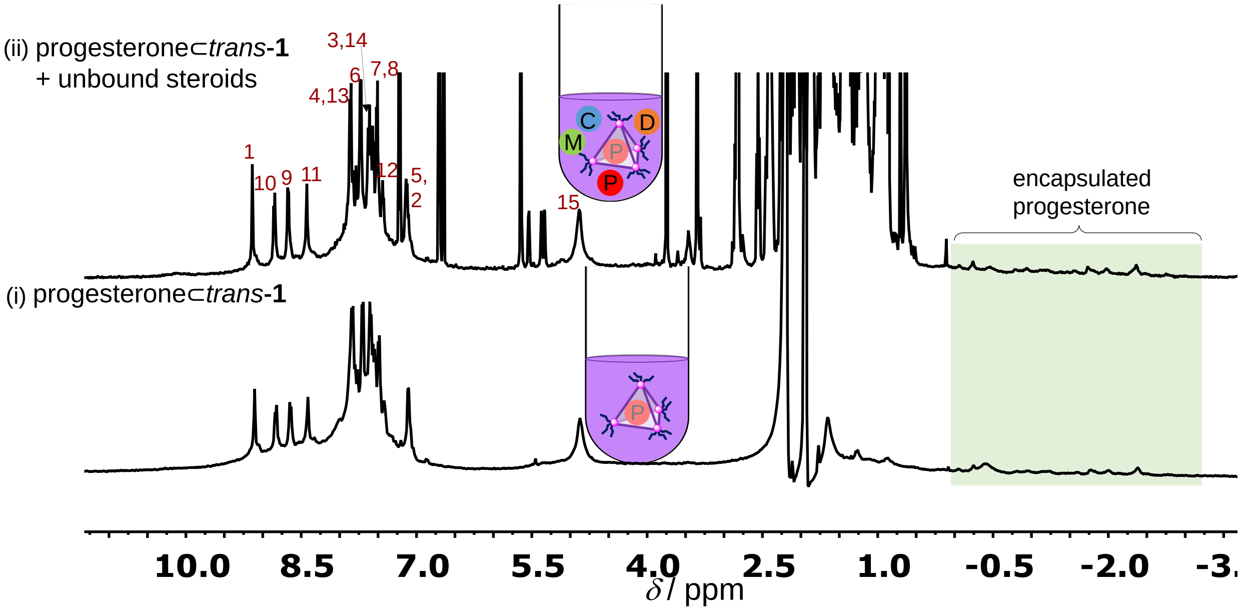

Figure S37: Comparison of ^{1}H NMR spectra (CD_3CN, 500 MHz, 25 °C) of (i) progesterone⊂*trans*-**1** and (ii) *trans*-**1** in the presence of the mixture of steroids. The peaks of the progesterone encapsulated within *trans*-**1** are highlighted.

6. Solubility of Steroids in Acetonitrile and Cyclopentane

6.1. ^{1}H NMR of Steroids in Cyclopentane

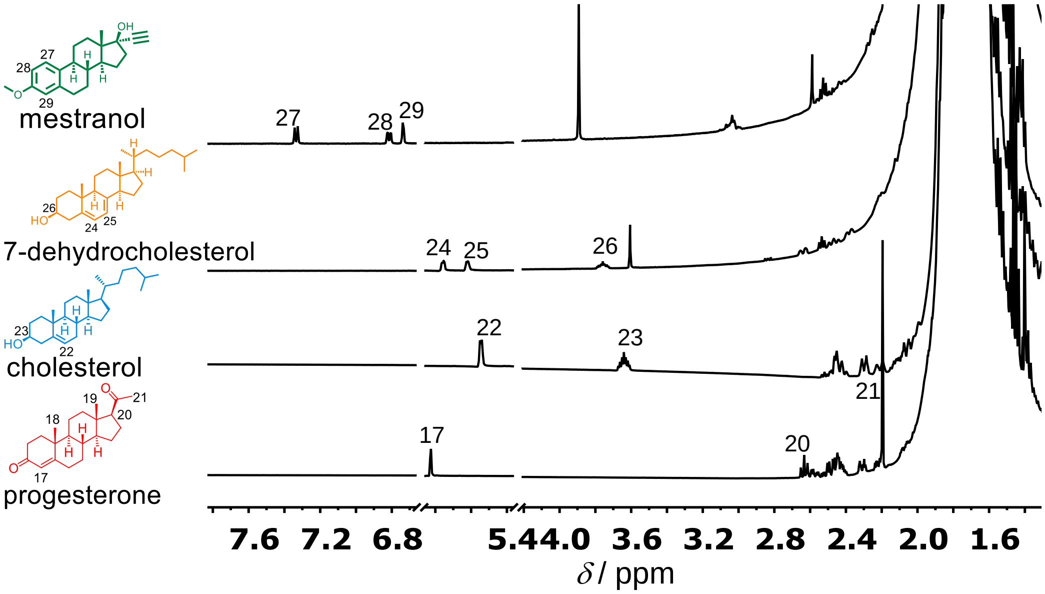

Figure S38: ^{1}H NMR (cyclopentane, 500 MHz, 25 °C) spectra of progesterone (c = 3.14 mM), cholesterol (c = 3.86 mM), 7-dehydrocholesterol (c = 3.84 mM) and mestranol (c = 3.11 mM). The NMR spectrometer was locked on CD_3CN inside a sealed capillary tube.

6.2. ^{1}H NMR of Steroids in CD_3CN

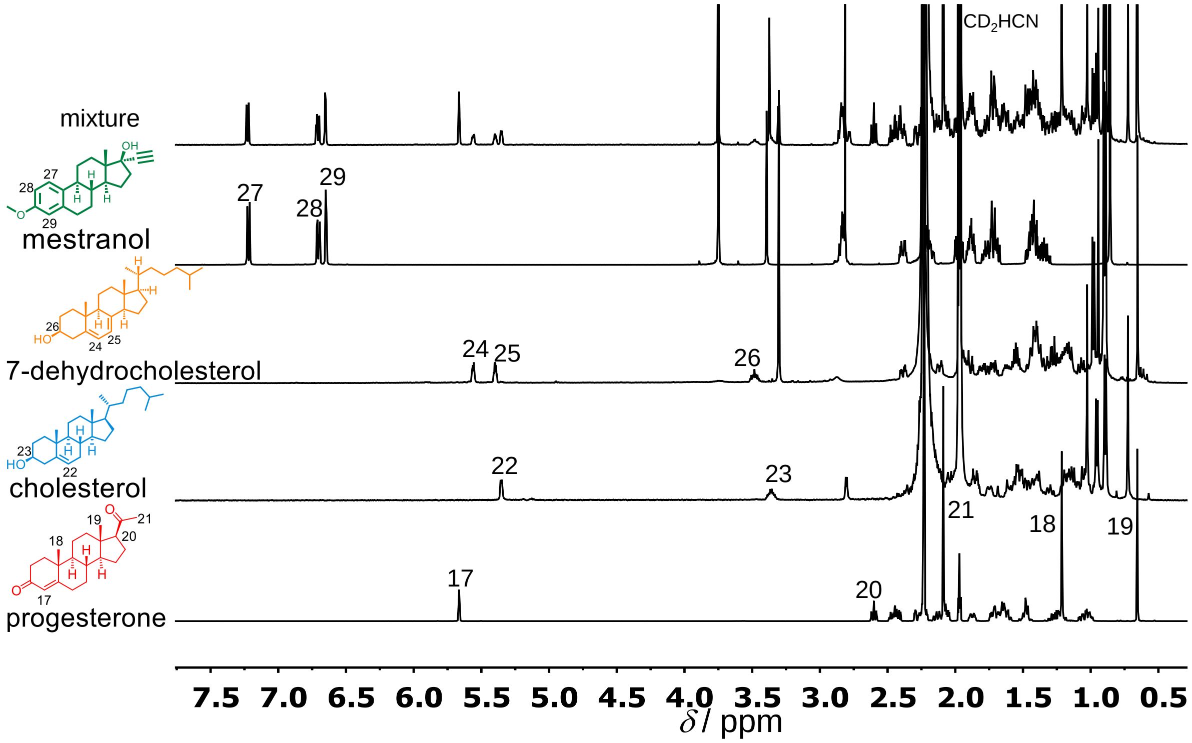

Figure S39: ^{1}H NMR (CD_3CN, 500 MHz, 25 °C) spectra of progesterone (c = 3.14 mM), cholesterol (c = 3.86 mM), 7-dehydrocholesterol (c = 3.84 mM) and mestranol (c = 3.11 mM), and a mixture of all four steroids.

6.3. Determination of Partition Coefficients

Using slice-selective ^{1}H NMR: The values of the partition coefficients were determined by slice-selective ^{1}H NMR. 1 mg of each steroid was initially dissolved in 250 µl of CD_3CN in an NMR tube, then 250 µl of cyclopentane was added. The sample was then shaken for 30 minutes to enable the steroids to distribute between the two solvents. The NMR tubes were then allowed to stand for 10 minutes to ensure that the two clear layers were well separated. At equilibrium, the distributions of the steroids were calculated using NMR integration using Phloroglucinol as an internal standard.

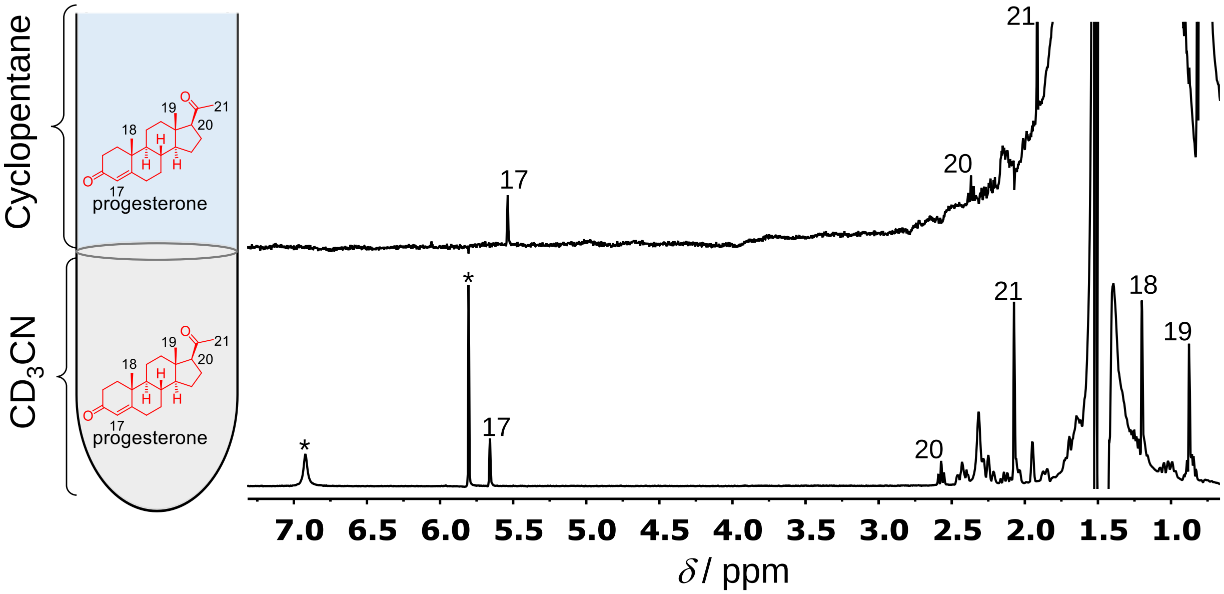

Figure S40: slice-selective ^{1}H NMR (CD_3CN/cyclopentane (1:1), 500 MHz, 25 °C) spectra of progesterone (c = 6.28 mM). Phloroglucinol (c = 6.28 mM) (marked by asterisks) is used as an internal standard to calculate the partition coefficient of progesterone between CD_3CN and cyclopentane. The experimental value of the partition coefficient ($\frac{[Cs]\text{Acetonitrile}}{[Cs]\text{Cyclopentane}}$) for progesterone is 9.06.

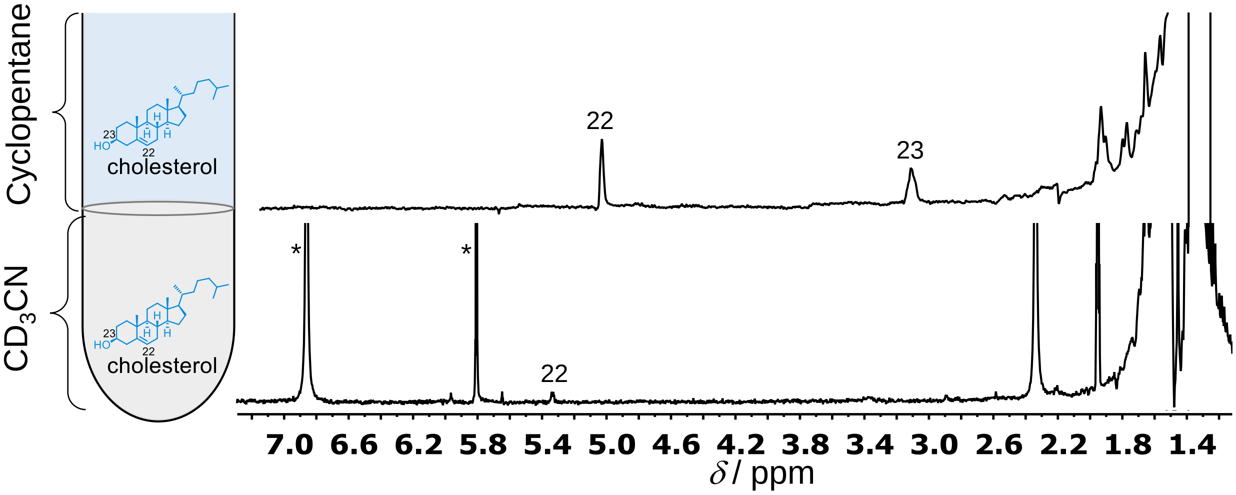

Figure S41: slice-selective ^{1}H NMR (CD_3CN/cyclopentane (1:1), 500 MHz, 25 °C) spectra of cholesterol (c = 7.72 mM). Phloroglucinol (c = 7.72 mM) (marked by asterisks) is used as an internal standard to calculate the partition coefficient of cholesterol between CD_3CN and cyclopentane. The experimental value of the partition coefficient ($\frac{[Cs]\text{Acetonitrile}}{[Cs]\text{Cyclopentane}}$) for cholesterol is 0.31.

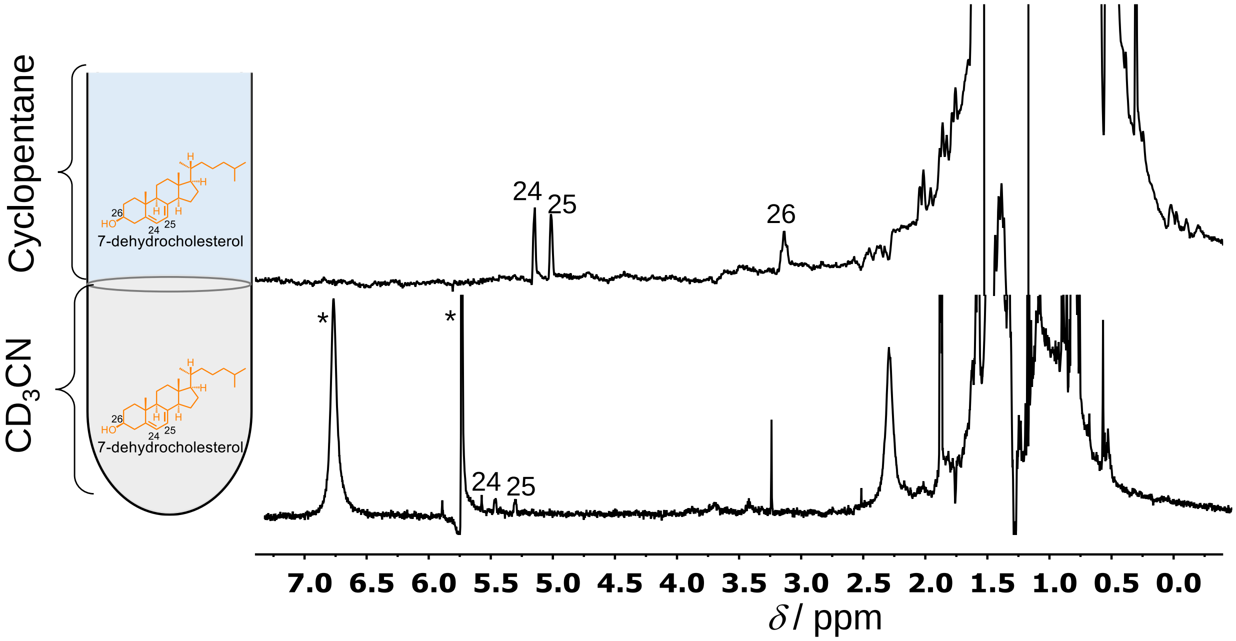

Figure S42: slice-selective ¹H NMR (CD_3CN/cyclopentane (1:1), 500 MHz, 25 °C) spectra of 7-dehydrocholesterol (c = 7.68 mM). Phloroglucinol (c = 7.68 mM) (marked by asterisks) is used as an internal standard to calculate the partition coefficient of 7-dehydrocholesterol between CD_3CN and cyclopentane. The experimental value of the partition coefficient ($\frac{[Cs]\text{Acetonitrile}}{[Cs]\text{Cyclopentane}}$) for 7-dehydrocholesterol is 0.71.

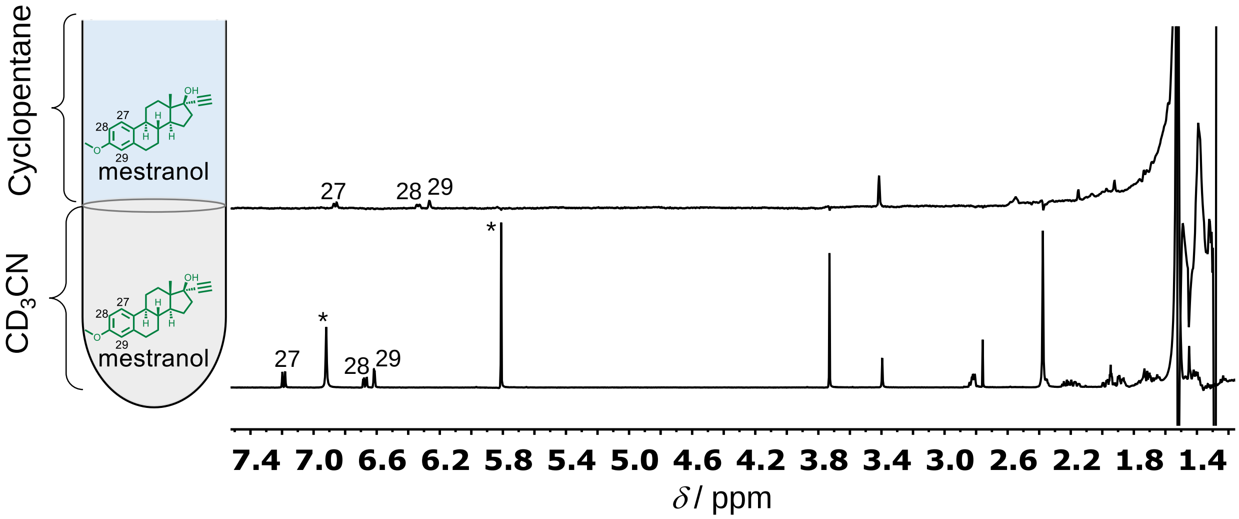

Figure S43: slice-selective ¹H NMR (CD_3CN/cyclopentane (1:1), 500 MHz, 25 °C) spectra of mestranol (c = 6.22 mM). Phloroglucinol (c = 6.22 mM) (marked by asterisks) is used as an internal standard to calculate the partition coefficient of mestranol between CD_3CN and cyclopentane. The experimental values of the partition coefficient ($\frac{[Cs]\text{Acetonitrile}}{[Cs]\text{Cyclopentane}}$) for mestranol is 9.51.

Using bulk extraction experiment: The partition coefficient of each steroid was measured from bulk extraction experiments. 100 mg of each steroid was added into a 1:1 mixture of acetonitrile and cyclopentane (10 ml). The sample was then shaken for 30 minutes for the steroid to distribute between the two solvents. The layers were then separated and evaporated to obtain the solid steroid. Then the partition coefficient of each steroid was calculated using following equation:

$$\text{partition coefficient} = \left(\frac{[Cs]\text{Acetonitrile}}{[Cs]\text{Cyclopentane}}\right)$$

The partition coefficients of progesterone, mestranol, cholesterol and 7-dehydrocholesterol were determined to be 9.20 (± 0.07), 9.34 (± 0.34), 0.34 (± 0.01) and 0.65 (± 0.02), respectively.

The partition coefficients presented in Fig. 2 were calculated using this extraction experiment.

Table S1. Partition coefficients for progesterone, mestranol, cholesterol and 7-dehydrocholesterol between acetonitrile and cyclopentane with no cage, in the presence of *trans*-**1**, and in the presence of disassembled **1**.

Systems	partition coefficients			
	progesterone	mestranol	cholesterol	7-dehydrocholesterol
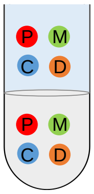	9.20 (± 0.07)	9.34 (± 0.34)	0.34 (± 0.01)	0.65 (± 0.02)
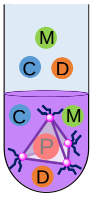	49.5 (± 2.10)	9.70 (± 0.50)	0.40 (± 0.02)	0.75 (± 0.02)
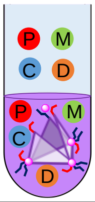	13.7 (± 0.17)	9.40 (± 0.31)	0.37 (± 0.01)	0.68 (± 0.02)

7. Extraction and Washing

Initially, *trans*-**1** (c = 0.97 mM) was prepared in an NMR tube in 500 µl CD_3CN as described above. Five equivalents of each steroid as solids were added. Progesterone was the only steroid encapsulated by *trans*-**1**, in slow exchange on the ^{1}H NMR chemical shift time scale, while the other steroids remained unbound. 500 µl of cyclopentane was then added to the mixture. The sample was then shaken for 30 minutes for the steroids to distribute between the two solvents. The NMR tubes were allowed to settle for 10 minutes to ensure that the two clear layers were well separated. Cyclopentane containing unbound steroids was then decanted from the NMR tube. For cholesterol and 7-dehydrocholesterol, only one extraction was sufficient, while for progesterone and mestranol, five repeated extractions were required to remove the excess steroids. After removal of cyclopentane, the CD_3CN was evaporated using a flow of N_2. Finally, fresh CD_3CN was used to record the NMR spectrum.

7.1. Washing with Cyclopentane to Remove Excess Progesterone

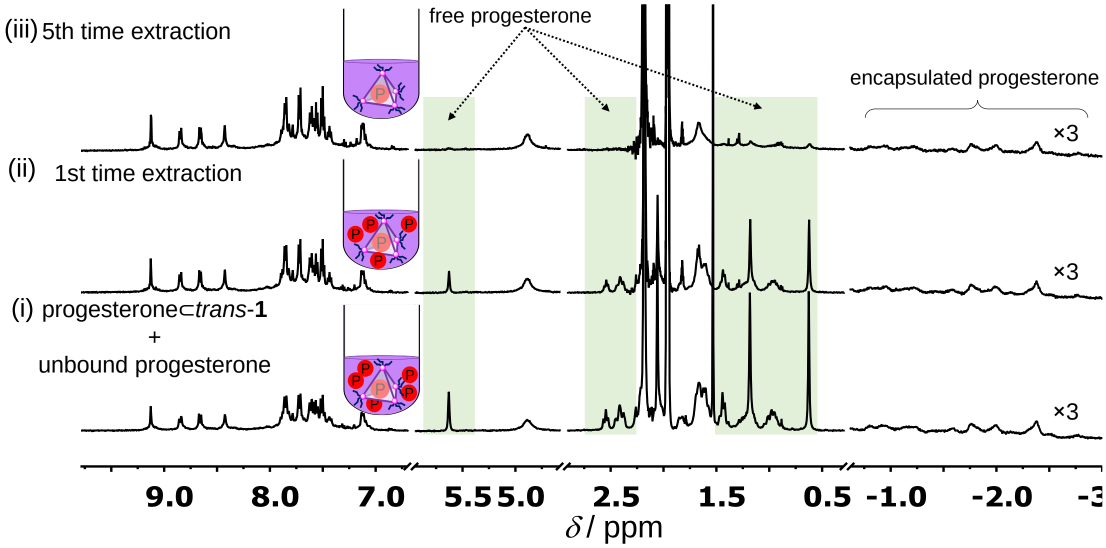

Figure S44: ^{1}H NMR (CD_3CN, 500 MHz, 25 °C) spectra of (i) *trans*-**1** (c = 0.97 mM) in the presence of excess progesterone (c = 4.85 mM). Removal of excess progesterone with (ii) 500 µl and (iii) 2.5 ml cyclopentane washings. The peaks of the unbound progesterone are highlighted.

7.2. Washing with Cyclopentane to Remove Unbound Cholesterol

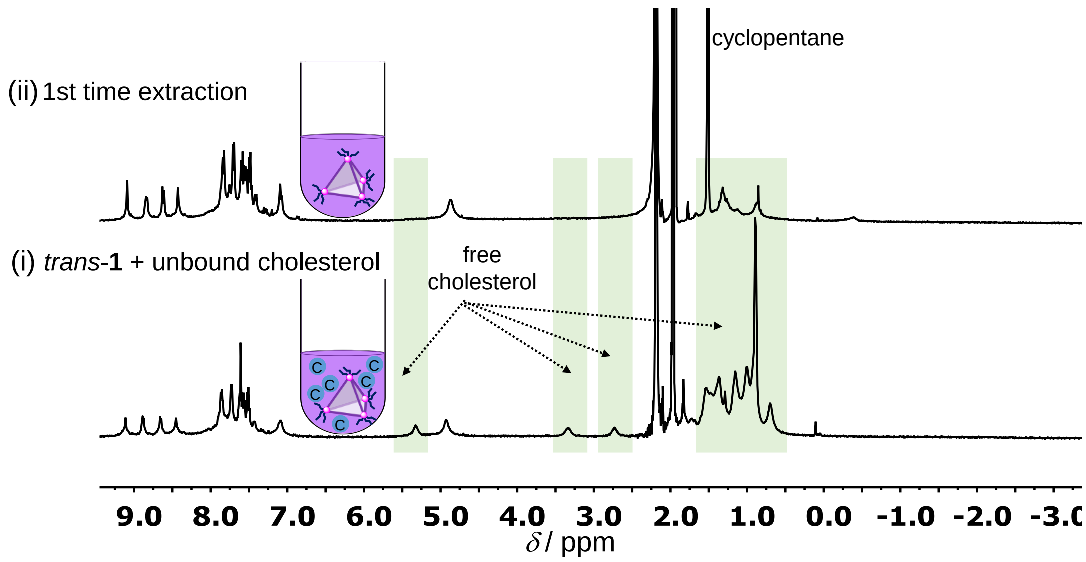

Figure S45: ^{1}H NMR (CD_3CN, 500 MHz, 25 °C) spectra of (i) *trans*-**1** (c = 0.97 mM) in the presence of excess cholesterol (c = 4.85 mM). (ii) Removal of excess cholesterol with 500 µl of cyclopentane. The peaks of the unbound cholesterol are highlighted.

7.3. Washing with Cyclopentane to Remove Unbound 7-Dehydrocholesterol

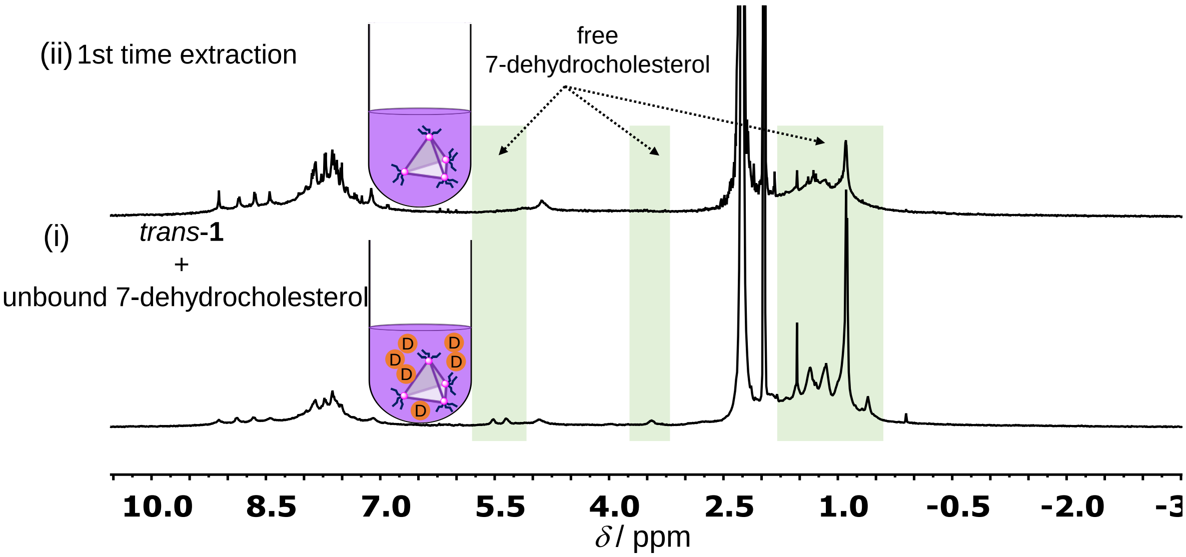

Figure S46: ^{1}H NMR (CD_3CN, 500 MHz, 25 °C) spectra of (i) *trans*-**1** (c = 0.97 mM) in the presence of excess 7-dehydrocholesterol (c = 4.85 mM). (ii) Removal of excess 7-dehydrocholesterol with 500 µl of cyclopentane washings. The peaks of the unbound 7-dehydrocholesterol are highlighted.

7.4. Washing with Cyclopentane to Remove Unbound Mestranol

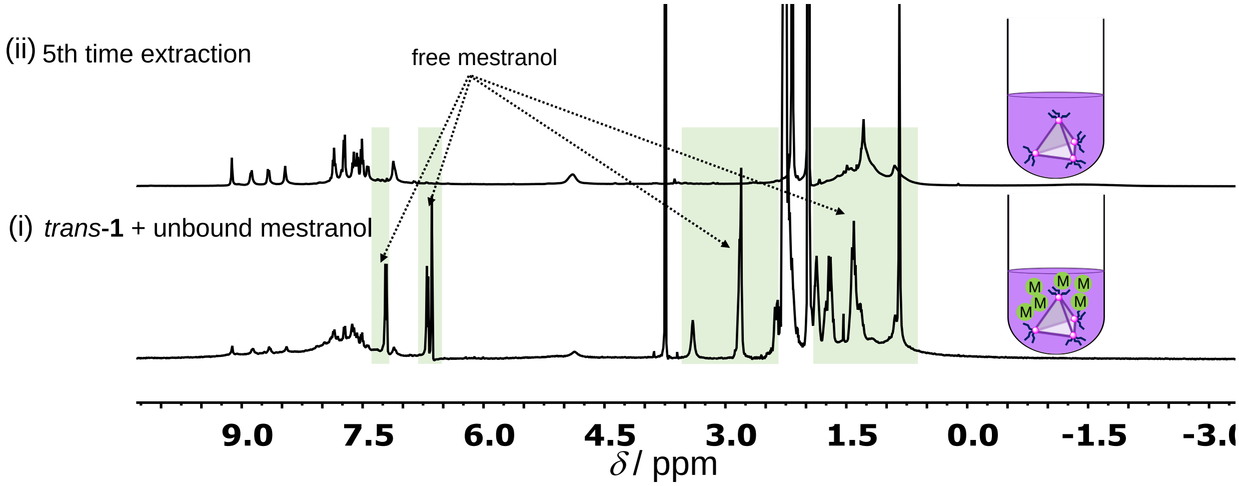

Figure S47: ^{1}H NMR (CD_3CN, 500 MHz, 25 °C) spectra of (i) *trans*-**1** (*c* = 0.97 mM) in the presence of excess mestranol (*c* = 4.85 mM). (ii) Removal of excess mestranol with 2.5 ml (5 × 500 µl) cyclopentane washings. The peaks of the unbound mestranol are highlighted.

7.5. Washing with Cyclopentane to Remove Unbound and Excess Steroids

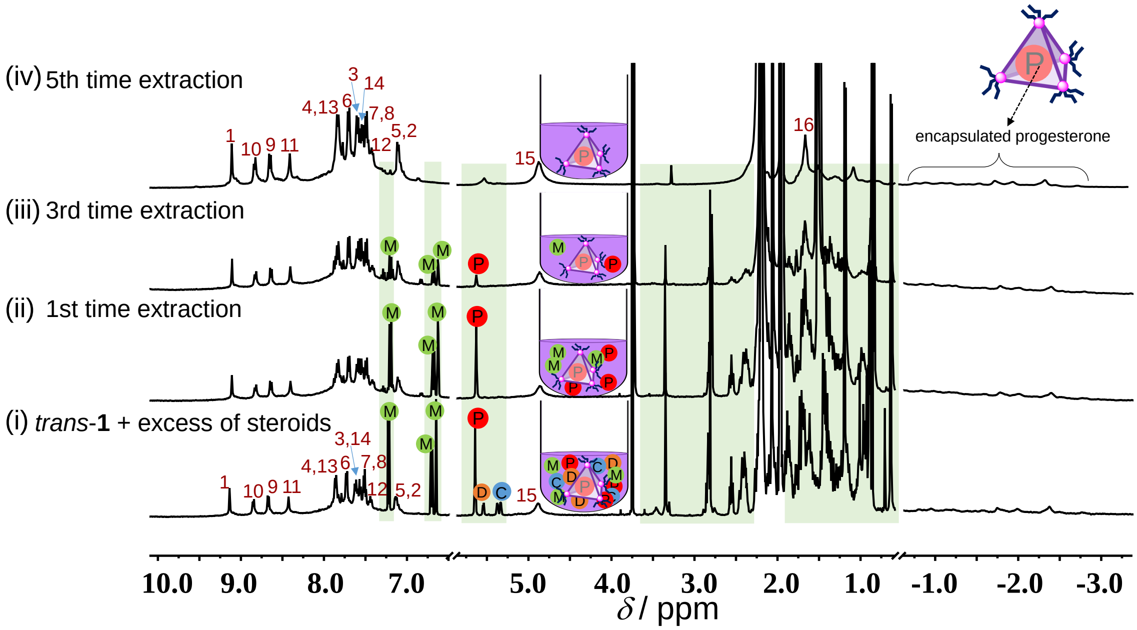

Figure S48: ^{1}H NMR (CD_3CN, 500 MHz, 25 °C) spectra of (i) *trans*-**1** (*c* = 0.97 mM) in the presence of four steroids in excess. Removal of unbound steroids with (ii) 500 µl; (iii) 1.5 ml and (iii) 2.5 ml of cyclopentane washings. The peaks of the unbound steroids are highlighted.

8. Photoswitching of Progesterone⊂*trans*-1 Following Exposure to Light at 350 nm and then at 500 nm

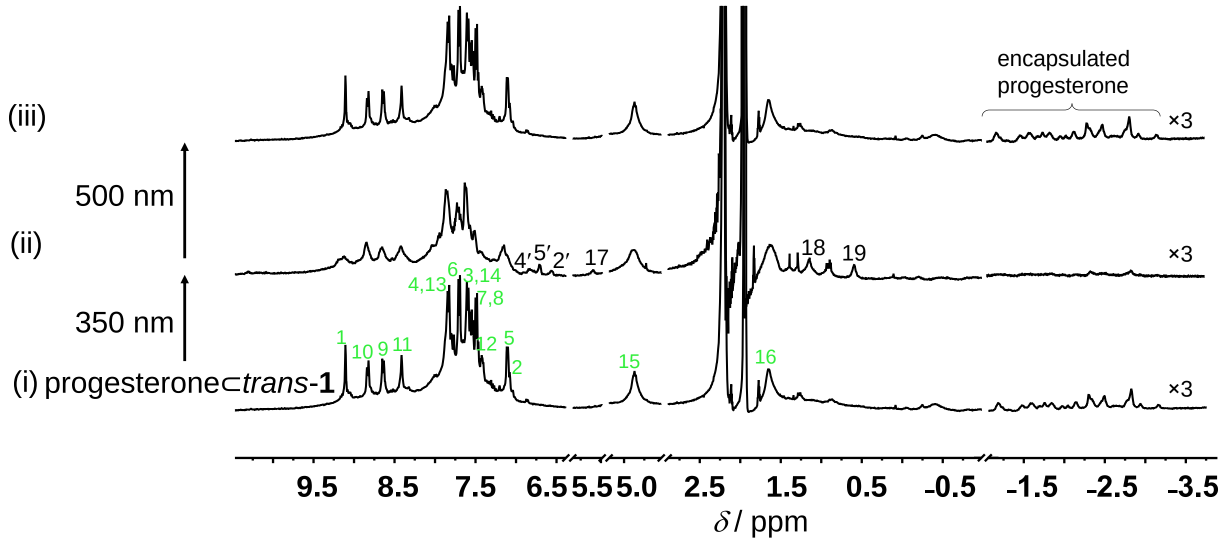

Figure S49: Reversible photoswitching of progesterone⊂*trans*-**1** (c = 1.6 mM). ^{1}H NMR spectra (500 MHz, CD_3CN, 25 °C) of (i) cage progesterone⊂*trans*-**1**; (ii) after 350 nm irradiation for 30 minutes and (iii) after irradiation at 500 nm for 30 minutes. The appearance of proton signals corresponding to *cis*-**B** (4′-H, 5′-H and 2′-H) after irradiation at 350 nm indicates dissociation of the cage. The signals 17-H, 18-H and 19-H are associated with progesterone release. The irradiations were performed in-situ placing the NMR tubes inside a Rayonet photochemical chamber reactor (40 cm deep, 25 cm diameter, 16 × 14 W light sources, operating temperature 32 °C).

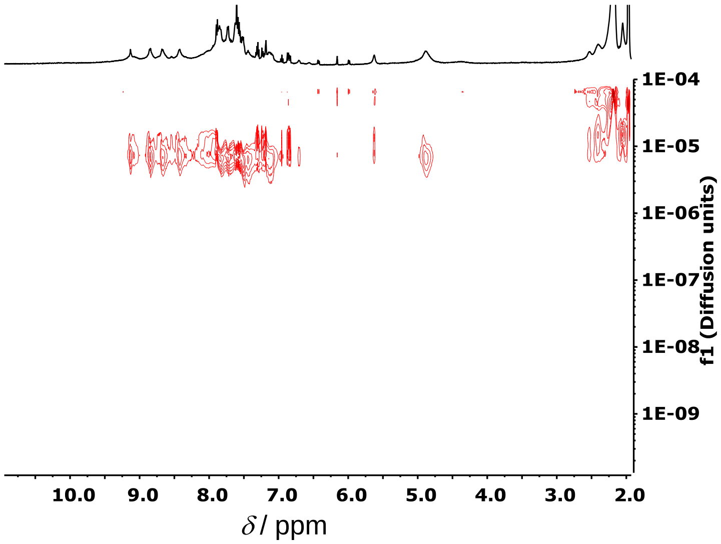

Figure S50: ^{1}H DOSY spectrum (CD_3CN, 500 MHz, 25 °C) of progesterone⊂*trans*-**1** after photoisomerization.

9. Light-Powered Progesterone Release from *trans*-1 in Presence of Cyclopentane

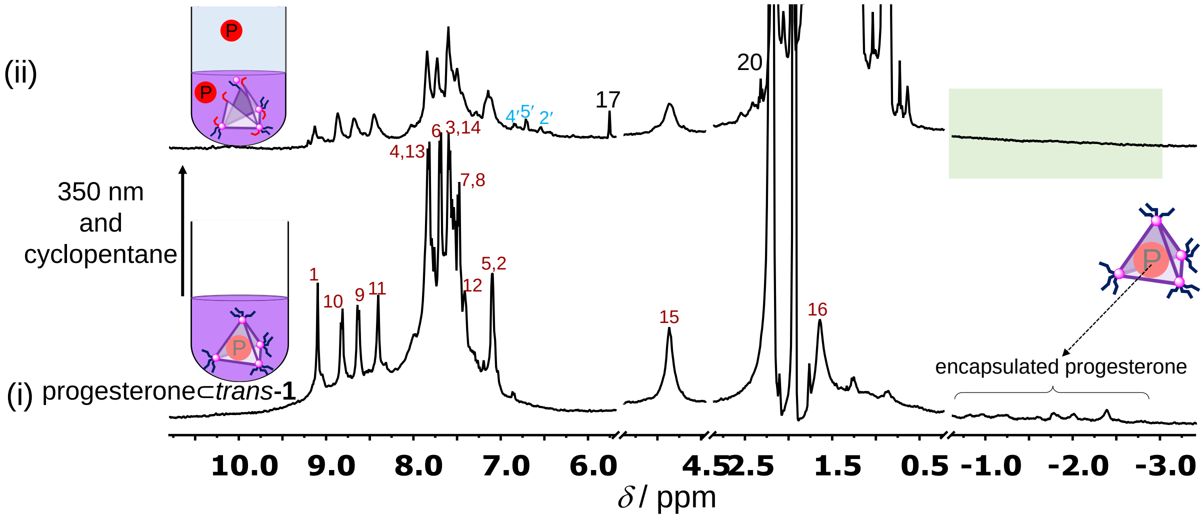

Figure S51: Photoswitching of progesterone⊂*trans*-**1** (c = 1.6 mM) in the presence of cyclopentane. ^{1}H NMR spectra (500 MHz, CD_3CN, 25 °C) of (i) progesterone⊂*trans*-**1**; (ii) after 350 nm irradiation for 30 minutes in the presence of 500 µl of cyclopentane. The appearance of proton signals corresponding to free progesterone (17-H and 20-H) suggests release of the guest. The highlighted area indicates no evidence of guest encapsulation. The irradiation was performed in-situ placing the NMR tubes inside a Rayonet photochemical chamber reactor (40 cm deep, 25 cm diameter, 16 × 14 W light sources, operating temperature 32 °C).

10. Purification of Progesterone from a Mixture of Steroids Using *trans*-1, Cyclopentane and Light

Initially *trans*-**1** (*c* = 5.09 mM) in 500 µl of CD_3CN was prepared in an NMR tube, as confirmed by ^{1}H NMR. Mestranol (*c* = 25.4 mM), progesterone (*c* = 25.4 mM), cholesterol (*c* = 25.4 mM) and 7-dehydrocholesterol (*c* = 25.4 mM) were then added. ^{1}H NMR indicated that one equivalent of progesterone with respect to *trans*-**1** was encapsulated by the cage, while the other steroids remained unbound. Then, 500 µl of cyclopentane was added to the mixture. The sample was then shaken for 30 minutes for the steroids to distribute between the two solvents. The NMR tube was left for 10 minutes to ensure that the two clear layers were well separated. Slice selective ^{1}H NMR then measured the distribution of steroids in both layers. Cyclopentane containing the unbound steroids was then decanted from the NMR tube. Five extractions, each time with 500 µl of cyclopentane, were required to remove all of the unbound steroids. The cyclopentane washes were combined and evaporated. The CD_3CN remaining the in NMR tube was evaporated under a flow of N_2. Fresh CD_3CN was used to dissolve the red solid product inside the NMR tube. ^{1}H NMR indicated the residual solid to be pure progesterone⊂*trans*-**1**. To this sample, 500 µl of

cyclopentane was added, followed by the irradiation at 350 nm. ^{1}H NMR after irradiation indicated the release of progesterone from the cage. Slice selective ^{1}H NMR showed the distribution of progesterone in both layers. Five extractions were again required to maximise the yield of pure progesterone. The CD_3CN in NMR tube was evaporated using a flow of N_2. Fresh CD_3CN was used to dissolve the solid product inside the NMR tube. Finally, the sample was irradiated at 500 nm to reassemble the cage. ^{1}H NMR indicated the residual solid to be pure *trans*-**1**. Six purification cycles were performed to recover 68% (± 4%) of the pure progesterone from the mixture.

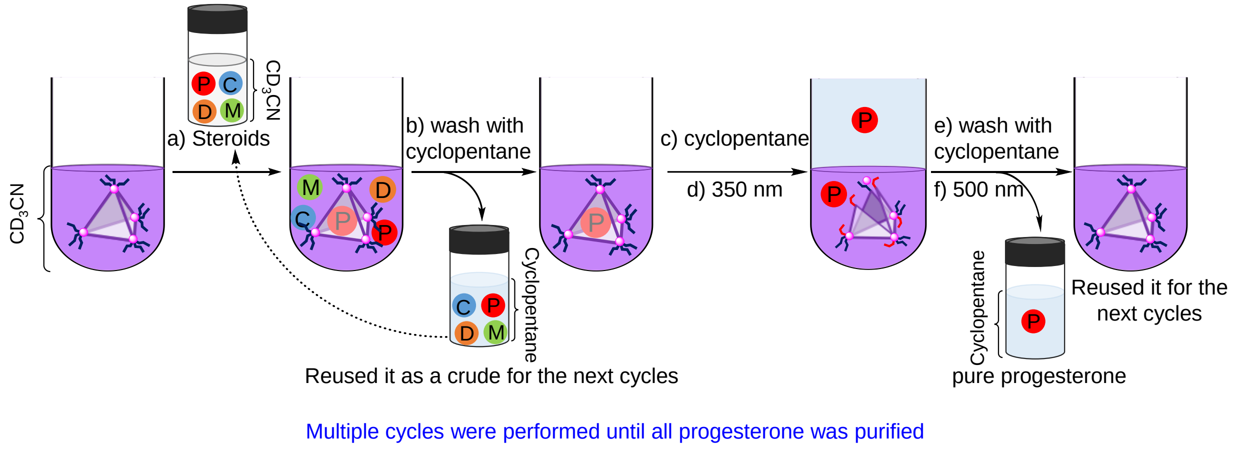

Figure S52: Purification cycles to extract pure progesterone from a mixture containing excess steroids.

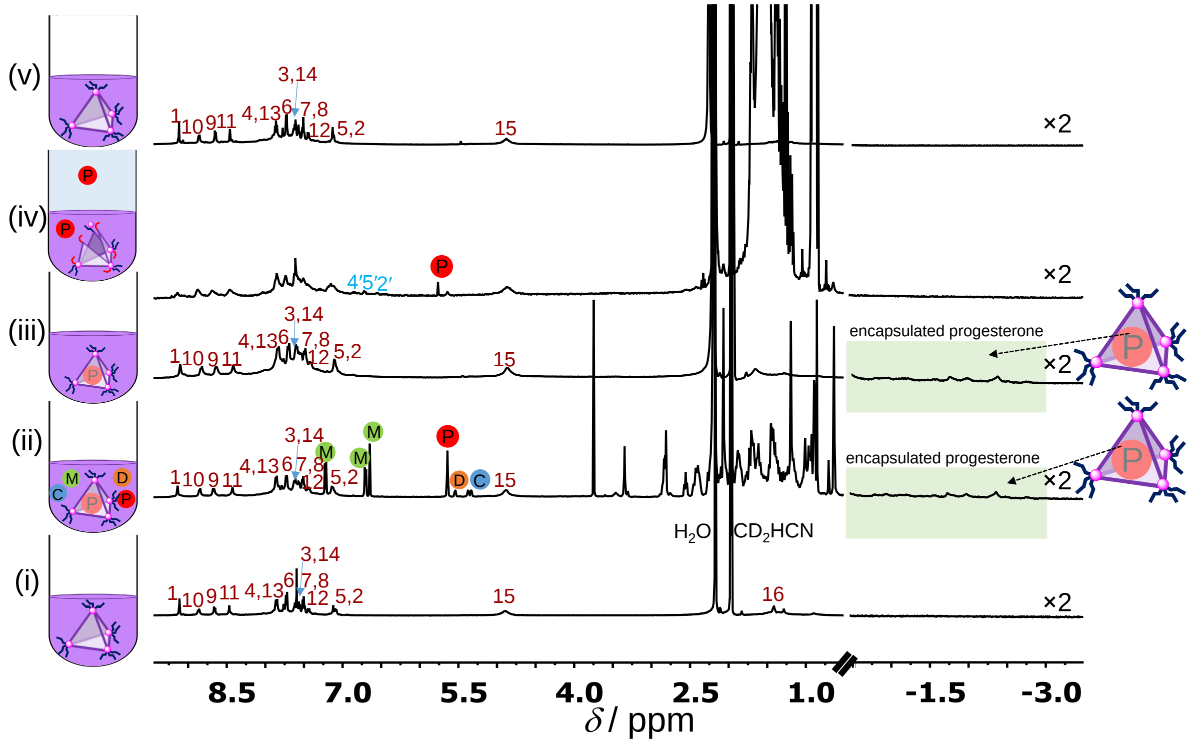

Figure S53: Purification of progesterone from a mixture of mestranol, cholesterol and 7-dehydrocholesterol. ^{1}H NMR spectra (500 MHz, CD_3CN, 25 °C) of (i) cage *trans*-**1**; (ii) after addition of 5 equiv. of each steroid; (iii) after extraction of unbound steroids with cyclopentane; (iv) after 350 nm irradiation for 30 minutes in presence of cyclopentane; (v) after extraction of unbound progesterone followed by 350 nm irradiation for 30 minutes. The peaks of the progesterone encapsulated within *trans*-**1** are highlighted.

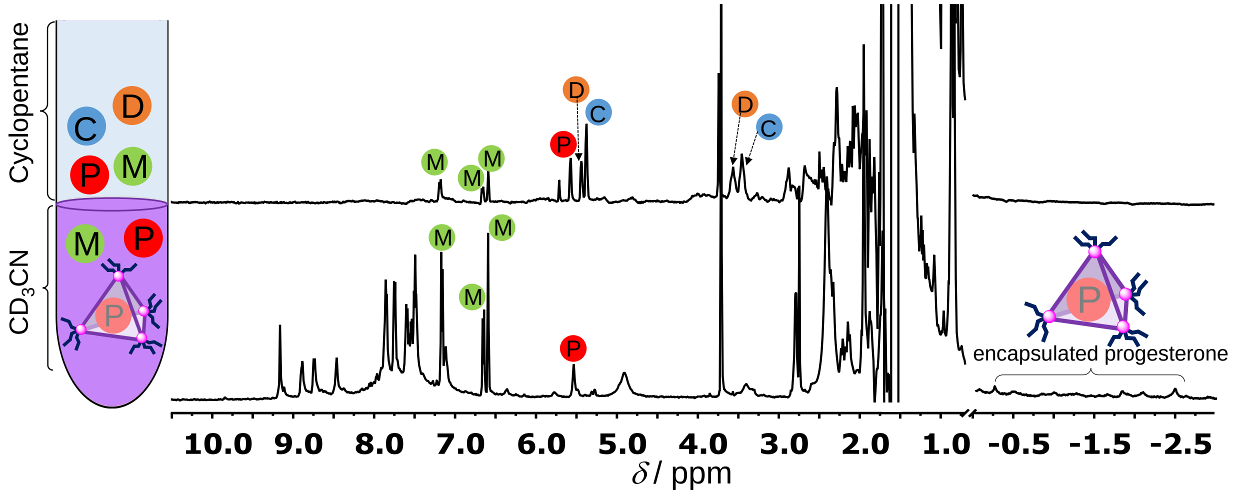

Figure S54: Slice-selective ^{1}H NMR of a mixture containing *trans*-**1**, progesterone, mestranol, cholesterol and 7-dehydrocholesterol.

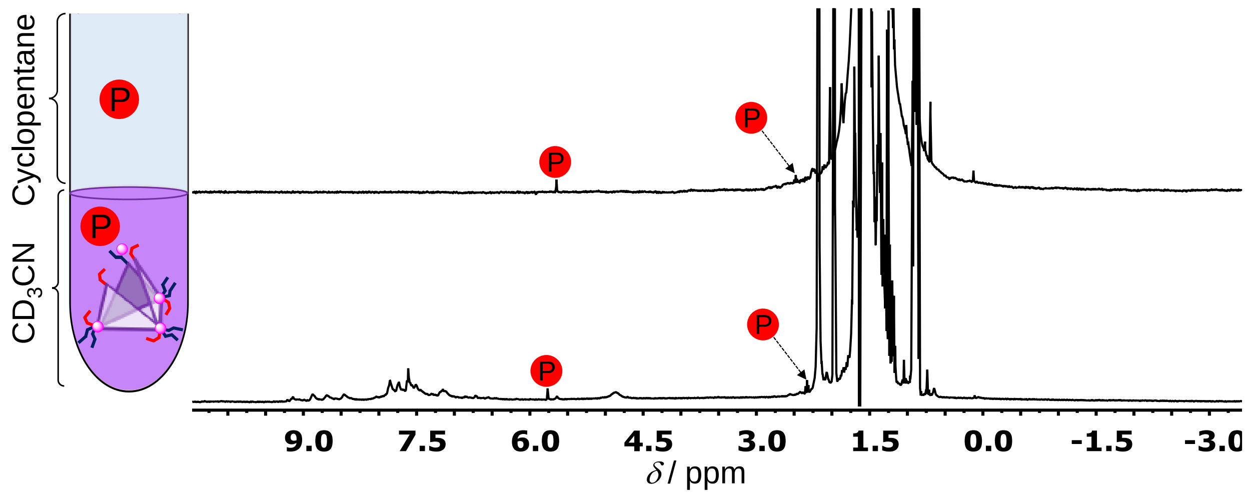

Figure S55: Slice-selective ^{1}H NMR of progesterone⊂*trans*-**1** after 350 nm light irradiation.

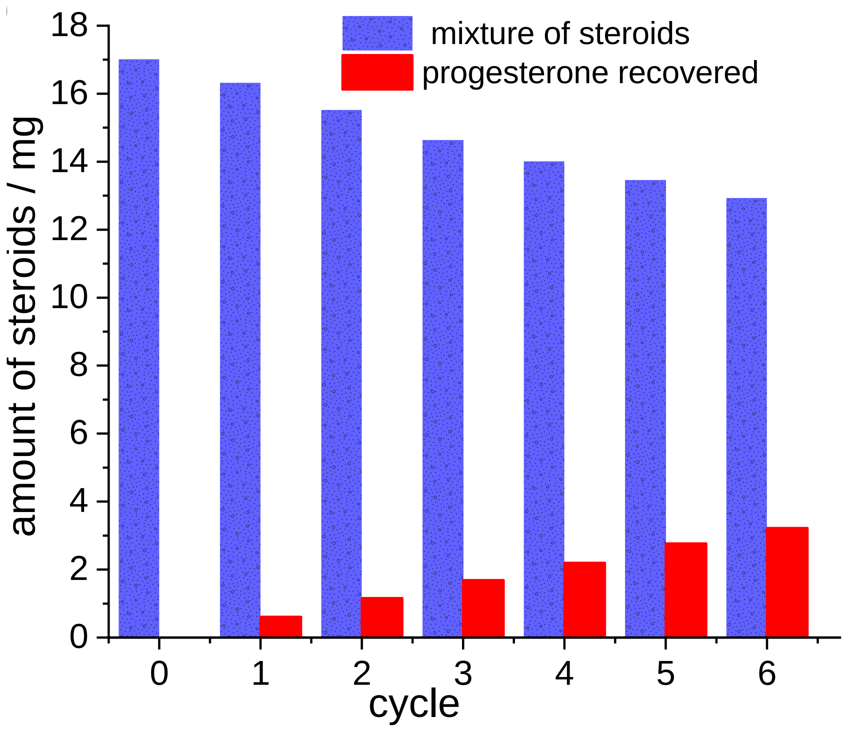

Figure S56: Plots showing purification of progesterone from the mixture. Multiple cycles needed to be performed to recover the maximum amount of progesterone.

11. Purification of Progesterone from a Stoichiometric Mixture of Steroids Using *trans*-1, Cyclopentane and Light

Initially *trans*-**1** (c = 6.36 mM) in 500 μl of CD_3CN was prepared in a NMR tube, as confirmed by ^{1}H NMR. Mestranol (c = 6.36 mM), progesterone (c = 6.36 mM), cholesterol (c = 6.36 mM) and 7-dehydrocholesterol (c = 6.36 mM) were then added. ^{1}H NMR indicated that progesterone was encapsulated by the cage while mestranol, cholesterol and 7-dehydrocholesterol remained unbound. Then, 500 μl of cyclopentane was added to the mixture. The sample was then shaken for 30 minutes for the steroids to distribute between the two solvents. The NMR tube was allowed to stand for about 10 minutes to ensure that the two clear layers were well separated. Cyclopentane containing mestranol, cholesterol and 7-dehydrocholesterol was then decanted from the NMR tube. Fresh CD_3CN was added to the NMR tube to make the volume up to 500 μl. ^{1}H NMR indicated the residual solid to be pure progesterone⊂*trans*-**1**. 500 μl of cyclopentane was then added before irradiation at 350 nm. An ^{1}H NMR spectrum was recorded after irradiation indicating the release of progesterone from the cage. Slice selective ^{1}H NMR was performed, showing the distribution of progesterone between both layers. Pure progesterone was then recovered by extraction.

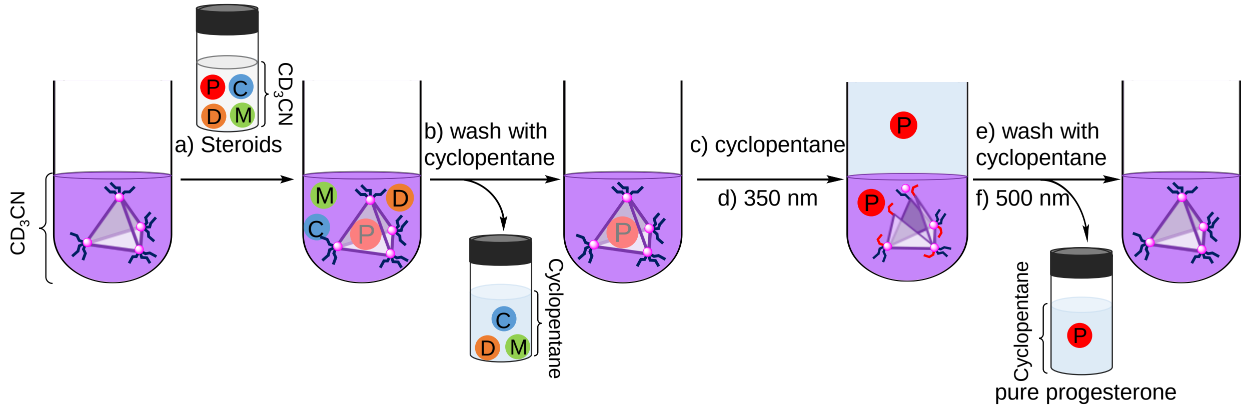

Figure S57: Extraction of progesterone from a mixture of steroids.

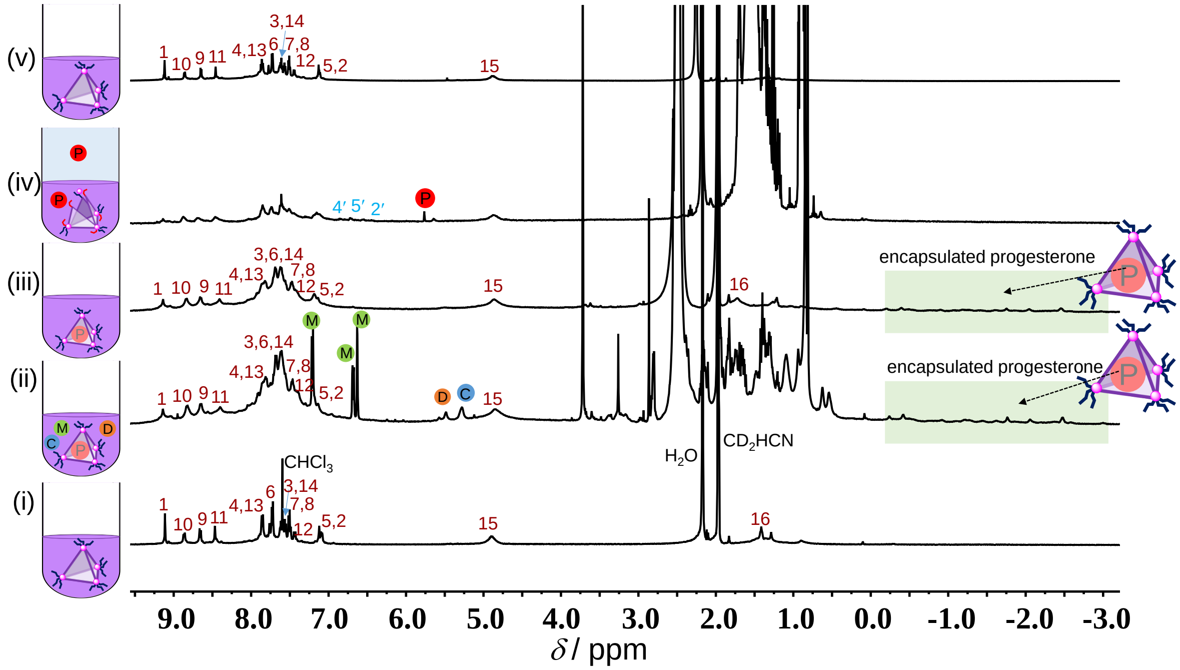

Figure S58: Purification of progesterone from a mixture of mestranol, cholesterol and 7-dehydrocholesterol. ^{1}H NMR spectra (500 MHz, CD_3CN, 25 °C) of (i) cage *trans*-**1**; (ii) after addition of 1 equiv. of each steroid; (iii) after extraction of mestranol, cholesterol and 7-dehydrocholesterol with cyclopentane; (iv) after 350 nm irradiation for 30 minutes in the presence of cyclopentane; (v) after removal of unbound progesterone followed by 350 nm irradiation for 30 minutes. The peaks of the progesterone encapsulated within *trans*-**1** are highlighted.

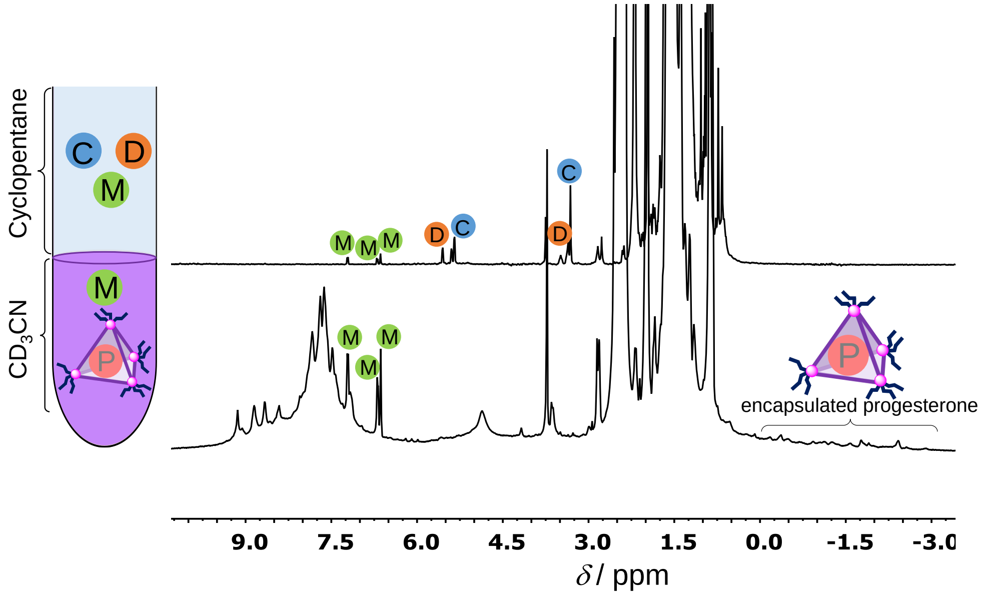

Figure S59: Slice-selective ^{1}H NMR of a mixture containing *trans*-**1**, progesterone, mestranol, cholesterol and 7-dehydrocholesterol.

12. Purification of Progesterone from a Mixture Containing Six Steroids Using *trans*-1, Cyclopentane Extraction, and Light

A mixture of 11-α-hydroxyprogesterone (6.36 mM), testosterone (6.36 mM), progesterone (6.36 mM), mestranol (6.36 mM), cholesterol (6.36 mM) and 7-dehydrocholesterol (6.36 mM) in 500 µl of CD_3CN was prepared in a NMR tube, as confirmed by ^{1}H NMR. Then, 500 µl of cyclopentane was added to the mixture. The sample was then shaken for 30 minutes for the steroids to partition between the two solvents. The NMR tube was allowed to stand for 10 minutes to ensure that the two clear layers were well separated. Cyclopentane containing progesterone, mestranol, cholesterol and 7-dehydrocholesterol was then decanted from the NMR tube. Then the cyclopentane was evaporated and then a solution of *trans*-**1** (6.36 mM) in 500 µl of CD_3CN was added. ^{1}H NMR indicated that progesterone was encapsulated by cage **1** while mestranol, cholesterol and 7-dehydrocholesterol remained unbound. Then, 500 µl of cyclopentane was added to the mixture. The sample was then shaken for 30 minutes for the steroids to partition between the two solvents. The NMR tube was allowed to stand for 10 minutes to ensure that the two clear layers were well separated. The cyclopentane layer containing mestranol, cholesterol and 7-dehydrocholesterol was then decanted from the NMR tube. ^{1}H NMR indicated the residual solid to be pure progesterone⊂*trans*-**1**. 500 µl of cyclopentane was then added before irradiation at 350 nm. The released progesterone was then recovered by extraction. Finally, the sample was irradiated at 500 nm to reassemble the cage. ^{1}H NMR indicated the residual solid to be pure *trans*-**1**.

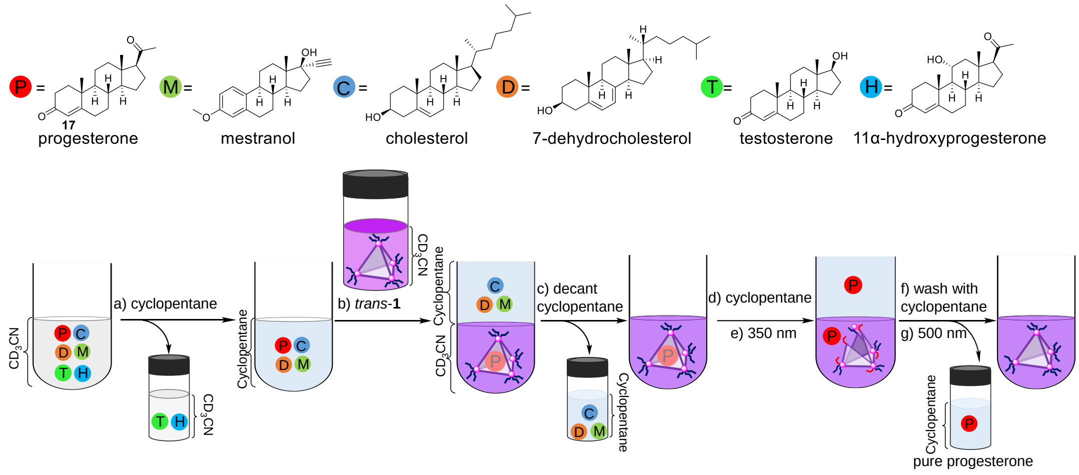

Figure S60: Extraction of progesterone from a mixture of structurally similar steroids.

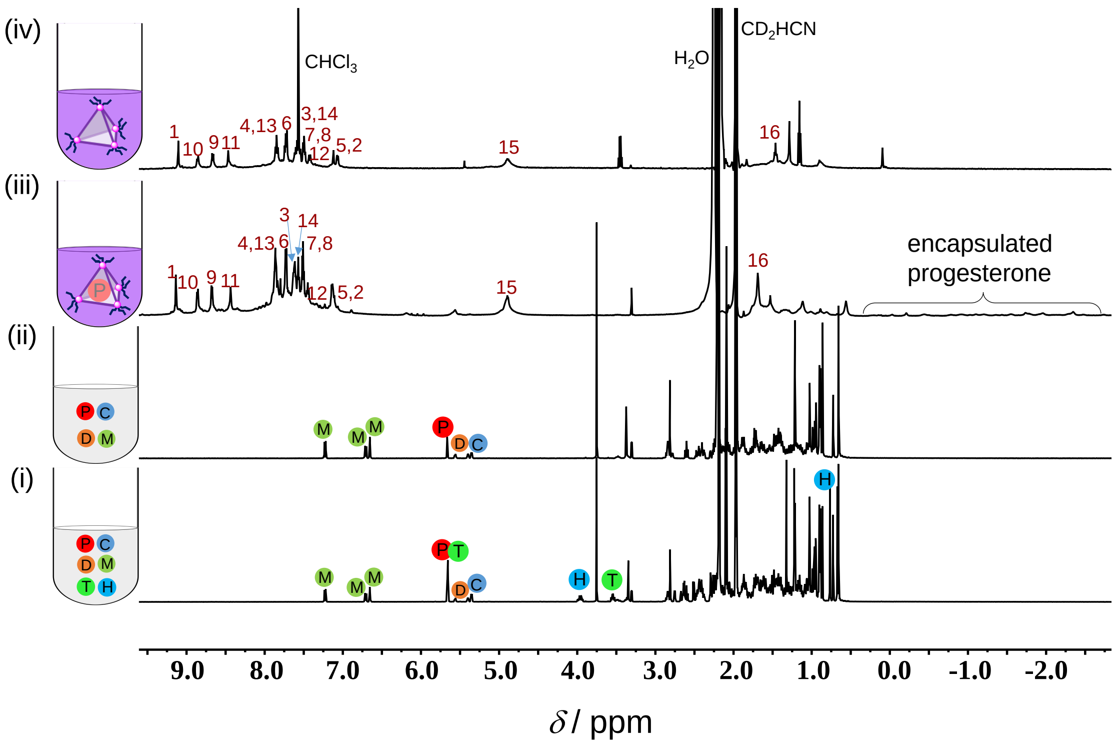

Figure S61: Purification of progesterone from a mixture of 11-α-hydroxyprogesterone, testosterone, mestranol, cholesterol and 7-dehydrocholesterol. ^{1}H NMR spectra (500 MHz, CD_3CN, 25 °C) of (i) the steroid mixture; (ii) after removal of 11-α-hydroxyprogesterone and testosterone; (iii) after addition of cage *trans*-**1** followed by extraction of unbound mestranol, cholesterol and 7-dehydrocholesterol with cyclopentane; (iv) after irradiation at 350 nm to purify progesterone, followed by 500 nm irradiation to reassemble the cage.

13. Volume Calculations

In order to determine the available void space within the structure of cage *trans*-**1**, Molovol[4] calculations based on the optimized structures obtained in this study were performed. The cavity volume was calculated to be 715 Å^3 using the parameters tabulated below.

Probe mode: one probe

Probe radius: 1.2 Å

Grid resolution: 0.1 Å

Optimization depth: 4

The molecular volumes of steroids were calculated using MoloVol from their MM3-optimized structures.

Table S2: Calculated steroid volumes.

Steroid	Volume (Å^3)
progesterone	367
mestranol	355
cholesterol	485
7-dehydrocholesterol	479

14. MM3 Models

Structures were calculated using Scigress,[5] running MM2 followed by MM3 calculations until convergence occurred.

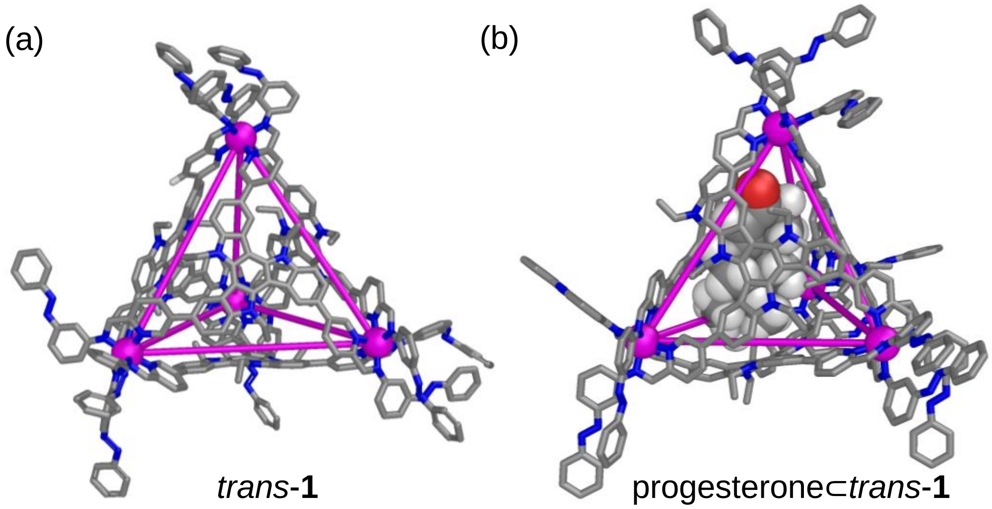

Figure S62. MM3-optimized molecular model of *trans*-**1** and progesterone⊂*trans*-**1**. The radii, measured as half of the distance between farthest-spaced hydrogen atoms, were calculated to be 18.9 Å and 24.8 Å for *trans*-**1** and progesterone⊂*trans*-**1** respectively, consistent with the increase in hydrodynamic radius observed by DOSY (Figures S7 and S20).

15. Purification of Progesterone from Testosterone Using *trans*-1, and Cyclopentane Extraction

Initially *trans*-**1** (5.09 mM) in 500 µl of CD_3CN was prepared in an NMR tube. Progesterone (c = 22.9 mM) and testosterone (c = 22.9 mM) were then added. ^{1}H NMR indicated that one equivalent of testosterone was encapsulated within *trans*-**1**, whereas the progesterone and excess testosterone remained unbound. Then, 500 µl of cyclopentane was added to the mixture. The sample was shaken for 30 minutes for the steroids to distribute between the two solvents. The NMR tube was left for 10 minutes to ensure that the two clear layers were well separated. Cyclopentane containing progesterone was then decanted from the NMR tube. Five extractions, each time with 500 µl of cyclopentane, were required to remove all of the progesterone.

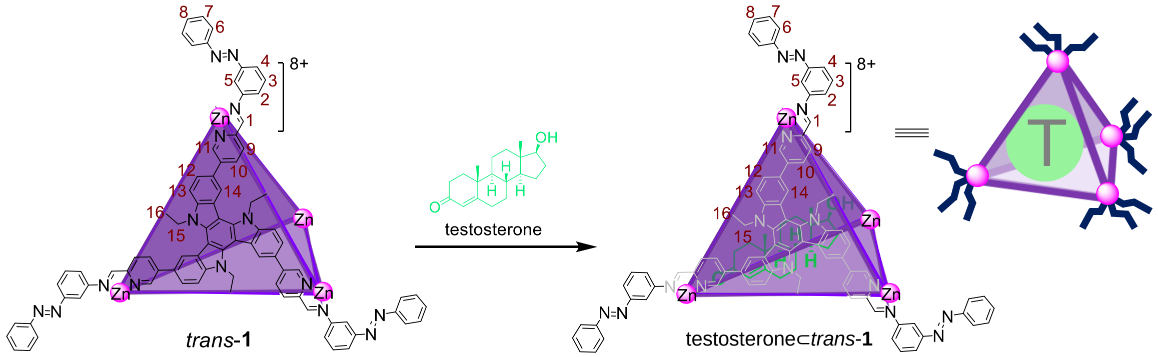

Figure S63. Encapsulation of testosterone as a guest. Initially *trans*-**1** (5.09 mM) was prepared in a NMR tube as described above. Testosterone was then added progressively, 0.5 equivalents at a time, as a solid. The ^{1}H NMR spectrum was recorded 30 minutes after each addition. Testosterone was found to be encapsulated by *trans*-**1**, in slow exchange on the ^{1}H NMR chemical shift time scale.

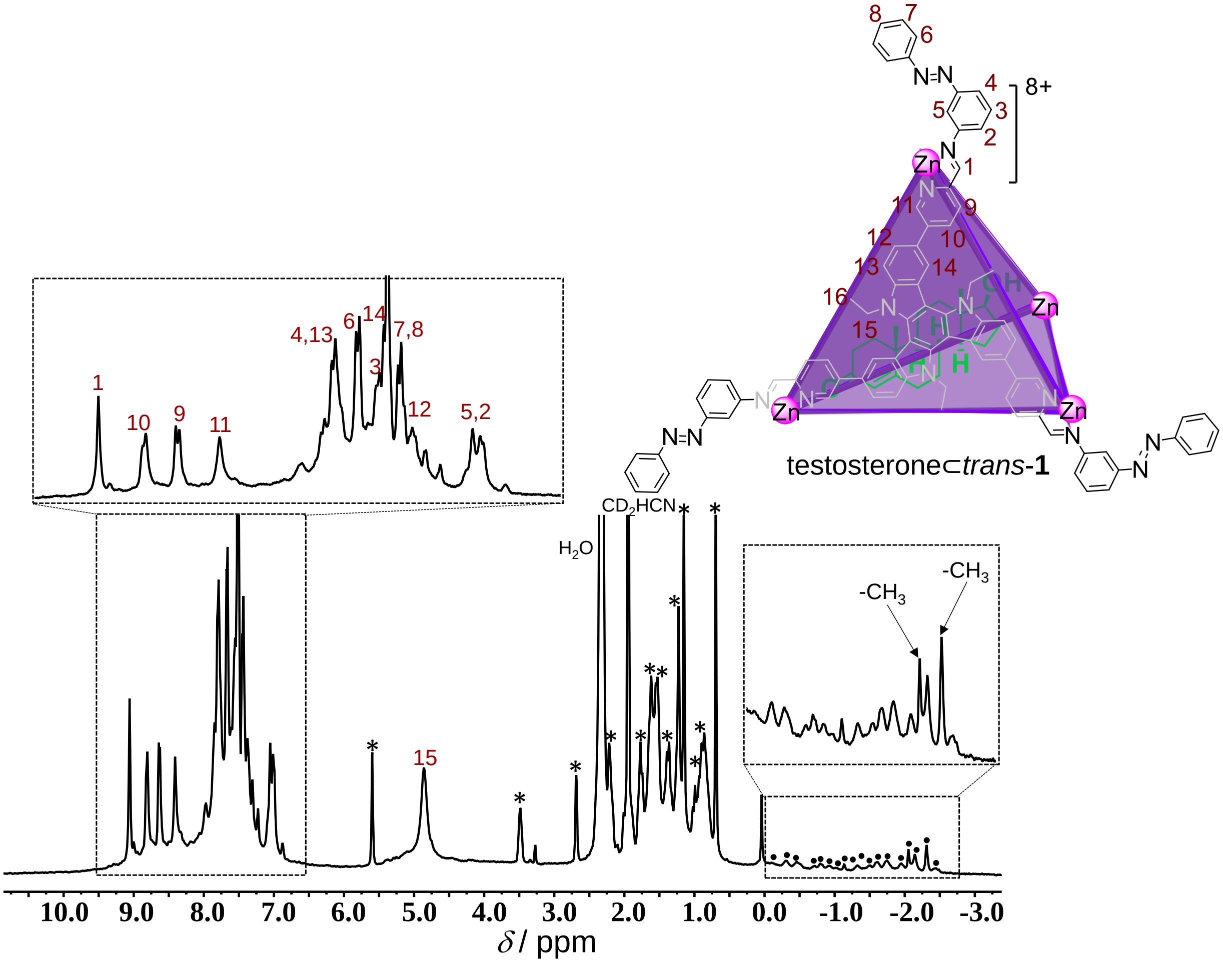

Figure S64. ^{1}H NMR (CD_3CN, 400 MHz, 0 °C) spectrum of testosterone⊂*trans*-**1**. Peaks corresponding to unbound testosterone are labelled with asterisks. The peaks corresponding to encapsulated guests are indicated by filled circles. The methyl peaks of the encapsulated testosterone guest are assigned as indicated.

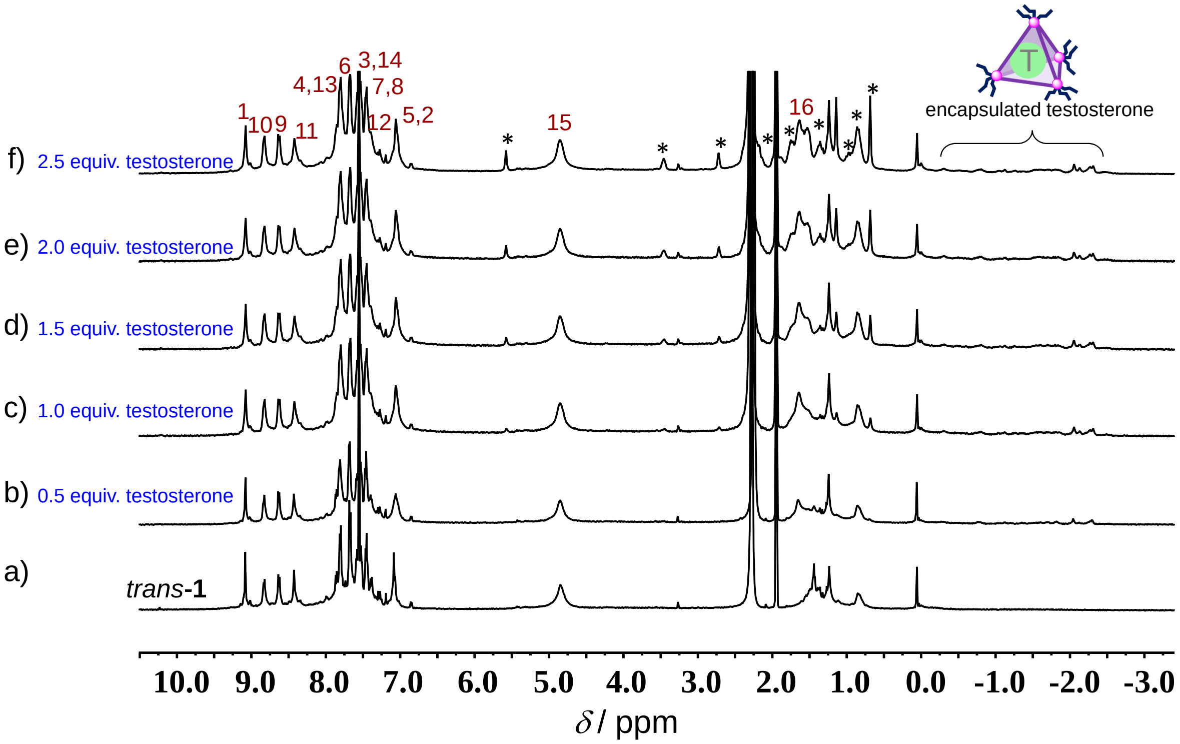

Figure S65. ^{1}H NMR (CD_3CN, 400 MHz, 0 °C) titration of *trans*-**1** (1.94 mM, a) with b) 0.5, c) 1.0, d) 1.5, e) 2.0, and f) 2.5 equiv. of testosterone. The peaks corresponding to free testosterone are labeled with asterisks.

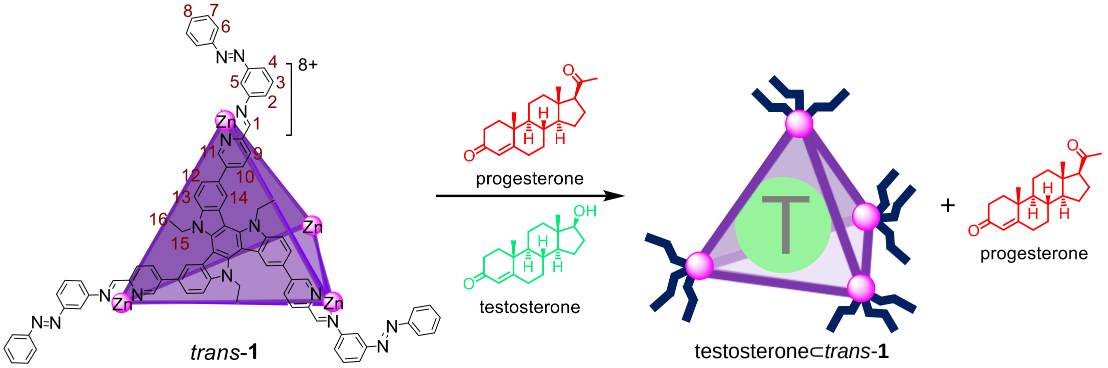

Figure S66. *trans*-**1** was observed to bind testosterone in preference to progesterone, as noted below.

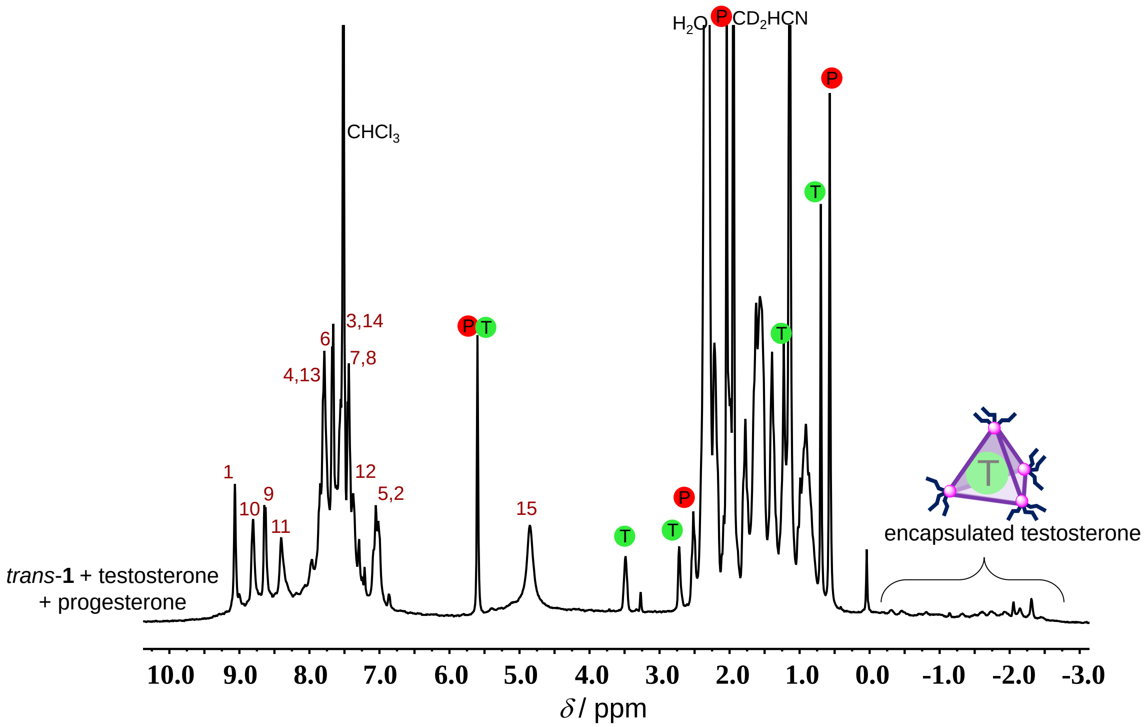

Figure S67. ^{1}H NMR spectrum (CD_3CN, 400 MHz, 0 °C) of *trans*-**1** in the presence of progesterone and testosterone. The peaks corresponding to free testosterone and progesterone are labeled.

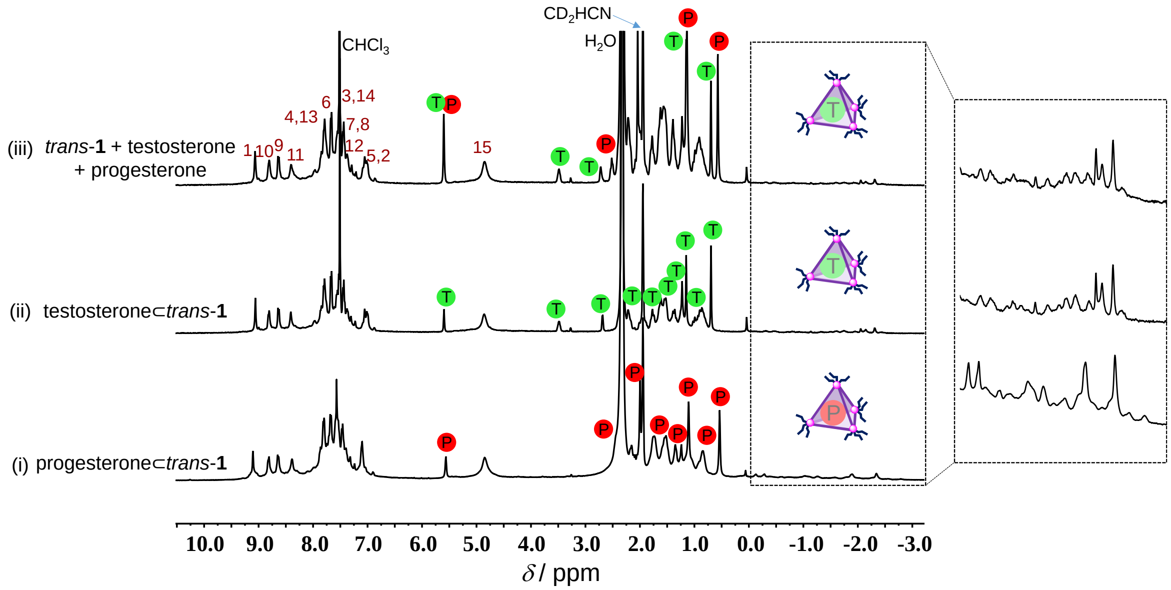

Figure S68. Comparison of ^{1}H NMR spectra (CD_3CN, 400 MHz, 0 °C) of (i) progesterone⊂*trans*-**1**, (ii) testosterone⊂*trans*-**1** and (iii) *trans*-**1** in the presence of progesterone and testosterone. The peaks corresponding to free testosterone and progesterone are labeled. The expanded spectra between 0 and -3 ppm at left indicated selective binding of testosterone with *trans*-**1** in the presence of progesterone.

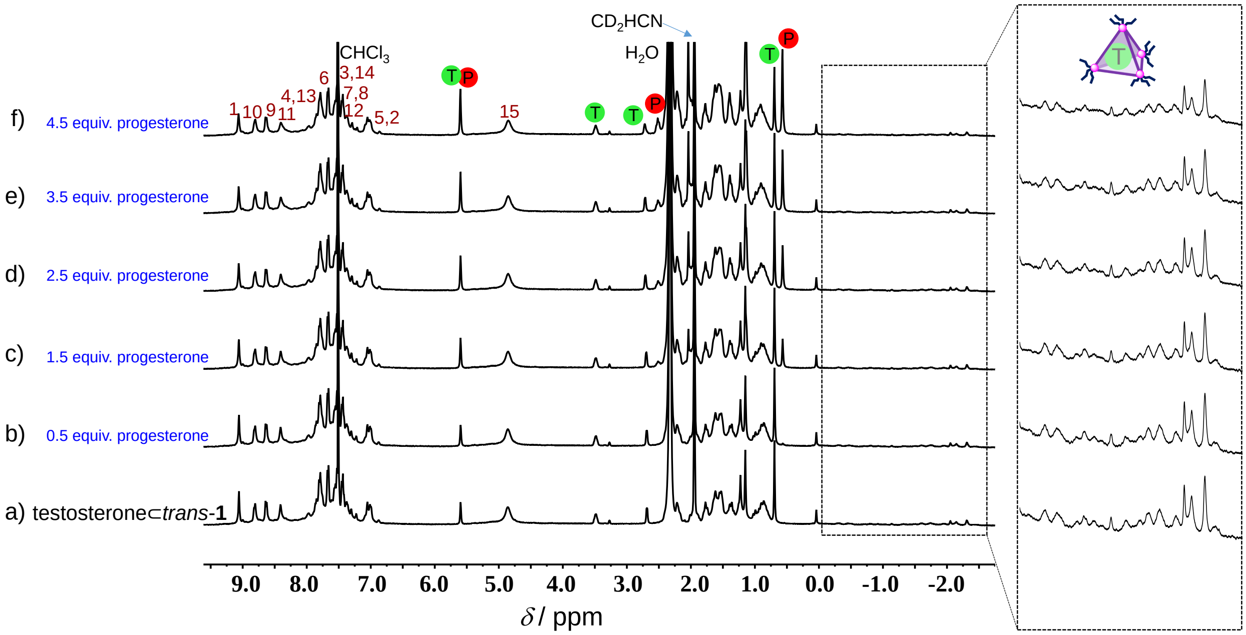

Figure S69. ^{1}H NMR (CD_3CN, 400 MHz, 0 °C) titration of testosterone⊂*trans*-**1** a) with b) 0.5, c) 1.5, d) 2.5, e) 3.5, and f) 4.5 equiv. of progesterone. The peaks corresponding to free testosterone and progesterone are labeled. The ^{1}H NMR spectra did not change with the progressive addition of progesterone, indicating a higher binding affinity for testosterone.

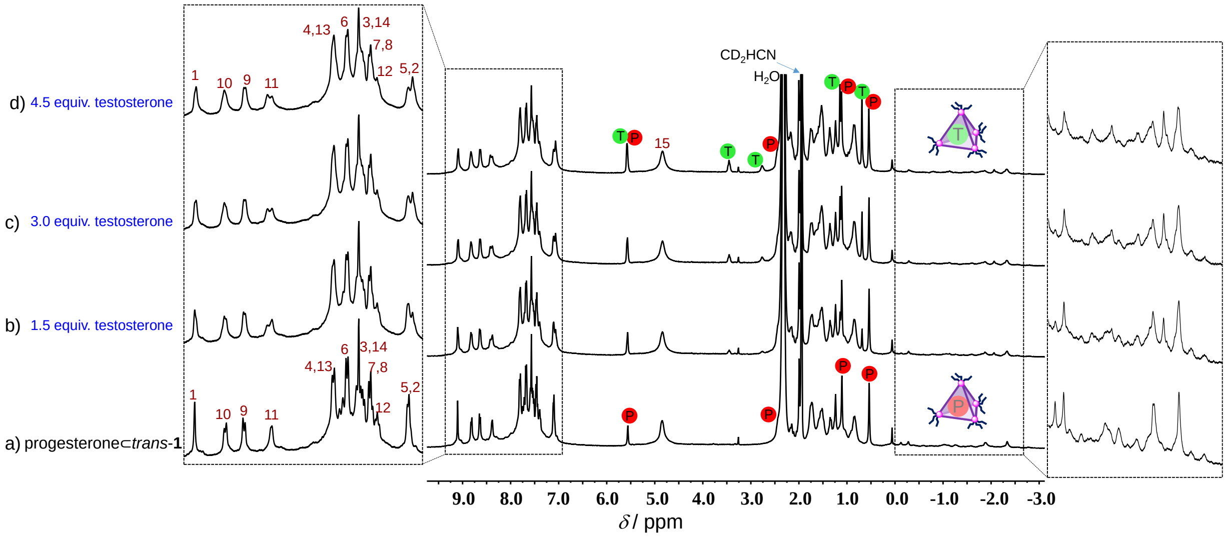

Figure S70. ^{1}H NMR (CD_3CN, 400 MHz, 0 °C) titration of progesterone⊂*trans*-**1** a) with b) 1.5, c) 3.0 and d) 4.5 equiv. of testosterone. The peaks corresponding to free testosterone and progesterone are labeled. ^{1}H NMR indicates displacement of progesterone by testosterone, indicating a higher binding affinity of testosterone than for progesterone.

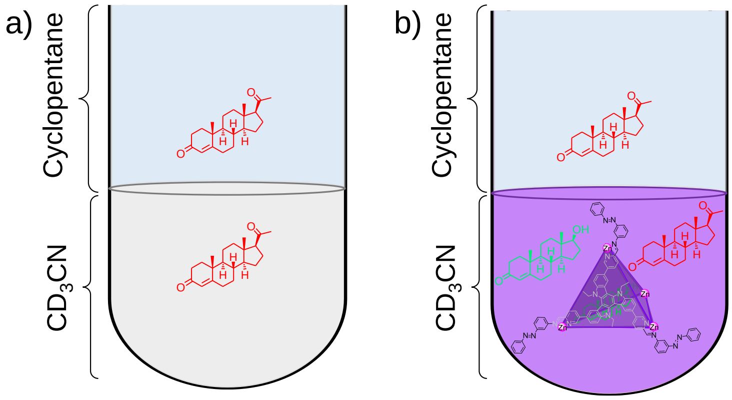

Figure S71. Partition of progesterone between acetonitrile and cyclopentane a) with no cage and b) in the presence of *trans*-**1** and testosterone. The partition coefficient of progesterone was unaltered by the presence of the cage, and determined to be 9.20 (± 0.07).

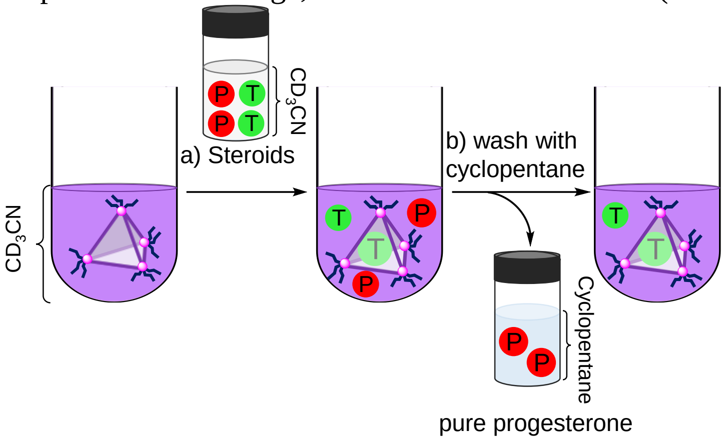

Figure S72. Extraction of progesterone from a mixture of progesterone and testosterone.

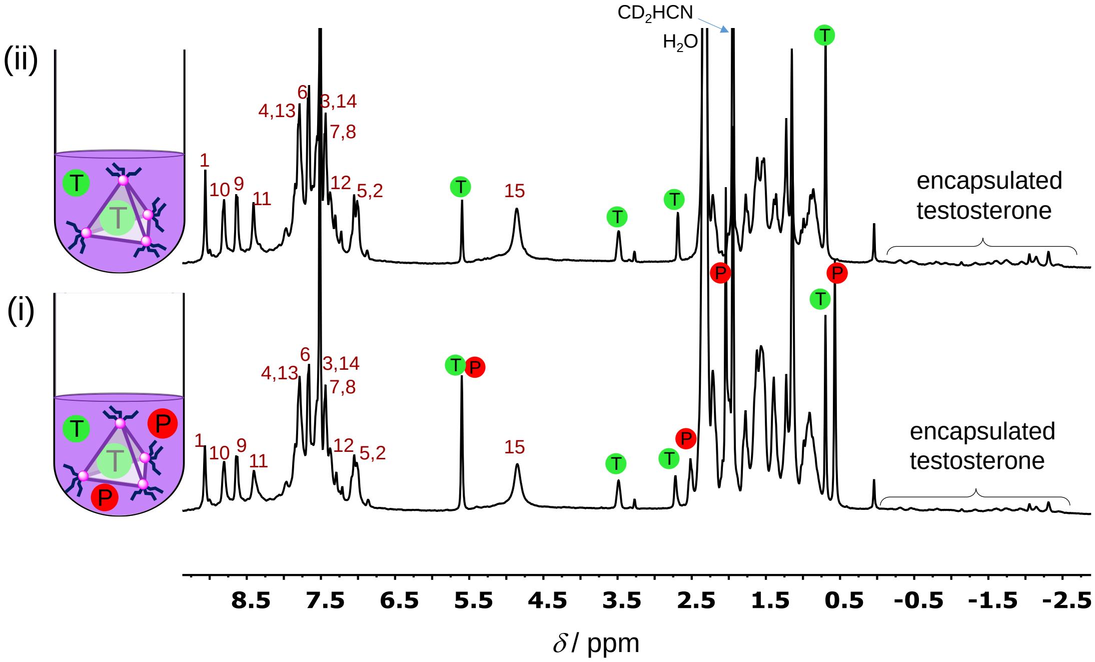

Figure S73. Purification of progesterone from testosterone. ^{1}H NMR spectra (400 MHz, CD_3CN, 0 °C) of (i) cage *trans*-**1** after the addition of 4.5 equiv. of progesterone and testosterone; (ii) after the extraction of progesterone with cyclopentane. The peaks corresponding to free testosterone and progesterone are labeled.

16. References

1. Li, G.; Ronson, T. K.; Lavendomme, R.; Huang, Z.; Fuertes-Espinosa, C.; Zhang, D.; Nitschke, J. R. Enantiopure $Fe^{II}{}_4L_4$ Cages Bind Steroids Stereoselectively. *Chem*. **2023**, *9*, 1–13.

2. Vapaavuori, J.; Goulet-Hanssens, A.; Heikkinen, I. T. S.; Barrett, C. J.; Priimagi, A. Are Two Azo Groups Better than One? Investigating the Photoresponse of Polymer-Bisazobenzene Complexes. *Chem. Mater*. **2014**, *26*, 5089−5096.

3. Grommet, A. B.; Hoffman, J. B.; Percástegui, E. G.; Mosquera, J.; Howe, D. J.; Bolliger, J. L.; Nitschke, J. R. Anion Exchange Drives Reversible Phase Transfer of Coordination Cages and Their Cargoes. *J. Am. Chem. Soc*. **2018**, *140*, 14770–14776.

4. Maglic, J. B.; Lavendomme, R., An Easy-to-Use Program to Calculate Various Volumes and Surface Areas of Chemical Structures and Identify Cavities. *J. Appl. Cryst*. **2022**, *55*, 1033-1044.

5. SCIGRESS software version FJ 2.6 (EU 3.1.9) Build 5996.8255.20141202, Fujitsu Limited, Tokyo, Japan, 2013.
